# Forefront Research of Foaming Strategies on Biodegradable Polymers and Their Composites by Thermal or Melt-Based Processing Technologies: Advances and Perspectives

**DOI:** 10.3390/polym16091286

**Published:** 2024-05-03

**Authors:** Luis F. F. F. Gonçalves, Rui L. Reis, Emanuel M. Fernandes

**Affiliations:** 1 3B’s Research Group, I3Bs–Research Institute on Biomaterials, Biodegradables and Biomimetics, University of Minho, Headquarters of the European Institute of Excellence on Tissue Engineering and Regenerative Medicine, AvePark, Parque de Ciência e Tecnologia, Zona Industrial da Gandra, Barco, 4805-017 Guimarães, Portugal; rgreis@i3bs.uminho.pt; 2 ICVS/3B’s—PT Government Associate Laboratory, Barco, 4805-017 Guimarães, Portugal

**Keywords:** biodegradable polymers, blowing agents, foams, foaming, lightweight, processing, blends, biocomposites

## Abstract

The last few decades have witnessed significant advances in the development of polymeric-based foam materials. These materials find several practical applications in our daily lives due to their characteristic properties such as low density, thermal insulation, and porosity, which are important in packaging, in building construction, and in biomedical applications, respectively. The first foams with practical applications used polymeric materials of petrochemical origin. However, due to growing environmental concerns, considerable efforts have been made to replace some of these materials with biodegradable polymers. Foam processing has evolved greatly in recent years due to improvements in existing techniques, such as the use of supercritical fluids in extrusion foaming and foam injection moulding, as well as the advent or adaptation of existing techniques to produce foams, as in the case of the combination between additive manufacturing and foam technology. The use of supercritical CO_2_ is especially advantageous in the production of porous structures for biomedical applications, as CO_2_ is chemically inert and non-toxic; in addition, it allows for an easy tailoring of the pore structure through processing conditions. Biodegradable polymeric materials, despite their enormous advantages over petroleum-based materials, present some difficulties regarding their potential use in foaming, such as poor melt strength, slow crystallization rate, poor processability, low service temperature, low toughness, and high brittleness, which limits their field of application. Several strategies were developed to improve the melt strength, including the change in monomer composition and the use of chemical modifiers and chain extenders to extend the chain length or create a branched molecular structure, to increase the molecular weight and the viscosity of the polymer. The use of additives or fillers is also commonly used, as fillers can improve crystallization kinetics by acting as crystal-nucleating agents. Alternatively, biodegradable polymers can be blended with other biodegradable polymers to combine certain properties and to counteract certain limitations. This work therefore aims to provide the latest advances regarding the foaming of biodegradable polymers. It covers the main foaming techniques and their advances and reviews the uses of biodegradable polymers in foaming, focusing on the chemical changes of polymers that improve their foaming ability. Finally, the challenges as well as the main opportunities presented reinforce the market potential of the biodegradable polymer foam materials.

## 1. Introduction

In material science, a foam can be understood as a structure composed of spherical voids, normally called cells, which are filled with gas and distributed within a denser polymeric matrix. The polymeric matrix, the number and size distribution of these voids or cells within the matrix, and their dimensional stability dictates the properties of the foam. In other words, polymer foams can be defined by their density, average cell size, cell density (understood as the number of cells per unit volume), and the volume expansion (ratio between the volume occupied by the cells and the volume of the polymer) [1]. Compared to bulky polymeric materials, polymer foams present many advantages depending on the specific application, such as low density, lower cost associated with a lower use of polymeric material, good heat and sound insulation, higher cushioning effect in the packaging of sensitive products, and high specific strength, among others [2,3,4]. Due to these advantages, polymeric foams have increasingly more practical applications in modern day life, namely in thermal insulation [5,6], in protective packaging [7,8,9], in cushioning [7,10,11], or in the health sector, in the development of scaffolds for applications in tissue engineering [12,13]. These applications come from the properties of polymeric foams compared to the properties of bulk polymeric solid materials, namely lower thermal conductivity, lower density without losing mechanical strength, porosity, and interconnected pores, essential in this last case for biocompatibility with living tissues [14,15,16,17,18].

Polymer foams can be prepared using several methods ranging from solvent casting up to melt-based processing methods, such as extrusion [19,20] and injection moulding [21,22]. This review will discuss only materials and foam preparation techniques based on processes involving the effect of temperature. This also includes non-conventional processes using supercritical fluids at low temperatures applied to biodegradable thermoplastic materials. Therefore, this review will not cover foams prepared by solvent casting, sol-gel [23], or salt leaching technologies and foams whose preparation process involves chemical polymerization reactions, as in the case of polyurethane foams [24].

Today, most of the polymeric materials produced worldwide come from non-renewable petrochemical-based sources. These plastics are increasingly used in areas such as packaging, the automotive industry, building materials, and frames for electrical and electronic devices, among others [25]. Another area were plastics find wide application is the area of disposable and single-use materials, which is responsible for the major and growing environmental problem associated with plastics. These materials do not undergo natural degradation and when discarded without proper recycling, they accumulate in landfills and remain there for many years or end up in the oceans, causing serious environmental problems [26].

Precisely the environmental problems associated with the increasing use of single-use and disposable plastic utensils have generated increasing environmental concerns. In this context and in line with some of the United Nations’ (UN’s) Sustainable Development Goals, there has been a growing interest in bioplastic materials in recent years. These so-called bio-based plastic materials are derived from biological sources and formed entirely or partially from renewable feedstock, such as biomass, or by microorganisms, employing less toxic reagents and solvents during the production process, resulting in bio-based or biodegradable polymers or both (i.e., bio-based and biodegradable polymeric grades) [27,28,29].

Equally important has been the focus on the polymer’s biodegradability, that is, its degradation by natural processes after use, normally by the action of naturally occurring microorganisms, avoiding the accumulation of plastics in landfills for many years [30]. Thus, biopolymers provide improved circularity and a lower carbon footprint by using renewable resources and biodegradation as an alternative end-of-life option besides adequate material properties with a view to practical application [28]. Some published review manuscripts discuss the shift in focus from polymers of petrochemical origin to the use of polymers of biological origin and the importance that biodegradability has on the environment [27,31,32].

In this context, biodegradable polymers, those who degrade due to the action of microorganisms present naturally in the environment without releasing any toxic or distinct residue, can be broadly divided in those that originate from renewable resources, such as poly (lactide acid) (PLA) [33] that is chemically synthetized from monomers produced from biomass, and those that are synthetized from monomers produced from petrochemical monomers, such as polycaprolactone (PCL) [34]. The biodegradable polymers have properties (mechanical, thermal, etc.) like those of more conventional petroleum-based polymers, such as polypropylene (PP), polyethylene (PE), polyethylene terephthalate (PET), etc. Currently, biodegradable polymers find increasingly more applications in the food packaging and agricultural sectors [35,36], with the global bioplastics production capacity expected to increase in the next five years from around 2.18 million tonnes in 2023 to approximately 7.43 million tonnes in 2028 [37].

This review provides a comprehensive overview of the recent developments in the foaming of biodegradable thermoplastic polymers, which is an area of material science that can help to reduce the negative impact on the environment that results from the massive use of non-renewable and non-biodegradable plastic materials. The first part provides the basics of the foaming technology, discussing the blowing agents and the foaming mechanisms. The following section is focused on the processes commonly used in the melt-based foaming of thermoplastic polymers. The last section provides the recent developments in thermal or melt-based foaming technologies for each of the main used biodegradable polymers, their blends, and composite materials.

**Figure 1:**
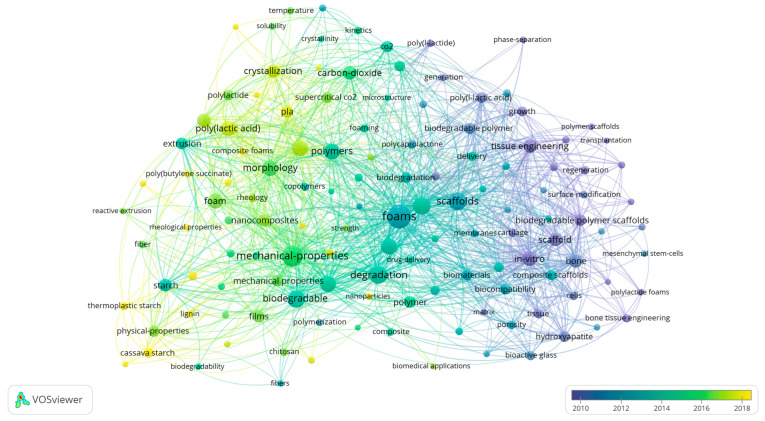
VOSviewer output of keyword co-occurrence relationship in papers published in biodegradable foaming polymers using melt-based or thermal processes, in Web of Science 2004–2023.

Figure 1 shows the graphical map of keywords most relevant to this study using VOSviewer co-occurrence analysis. This provides an overview of the evolution of this area in the last two decades and is based on a literature analysis in Web of Science with the key terms “biodegradable polymer” and “foam” or “foaming” and “melt-based” or “thermal process”. On the VOSviewer algorithm displayed below, lines connect the different terms, and the strength of the circle sizes indicates the number of occurrences of a keyword at least 10 times. After considering the experimental articles and retrieving the review manuscripts, it was observed that the term foams occurred with higher relevance. The software also highlights that it is possible to observe different clusters (purple colour) and that the use of melt-based technologies to produce biodegradable porous structures or scaffolds for tissue engineering applications occurred mainly in the first decade.

In the last decade (green and yellow colours), studies involving foams or foaming have focused on the main biodegradable polymers and their mixtures or composites and their characterization in terms of mechanical properties, morphology, crystallization, rheology, and biodegradability aspects. Reinforcing agents such as cellulose, lignin, fibres, chitosan, and nanoparticles are also considered to improve the foam properties. Moreover, the use of non-conventional technologies, such as supercritical CO_2_ for foaming and reactive extrusion, are also highly mentioned. Regarding the polymer matrices, PLA has a higher prevalence in the more recently published research studies and is followed by starch, poly(butylene succinate) (PBS), PCL, its blends, and its composite foams.

## 2. Basics of Foaming Technology

In thermoplastic polymers, foaming is achieved by adding a blowing agent to the thermoplastic material, whose expansion inside the material will give rise to the desired cellular structure. This expansion of the blowing agent is normally triggered by a sudden disturbance, for example, a drop in pressure or increase in temperature, of the polymer/blowing agent mixture [38]. Typically, the blowing agent could be either of a chemical or physical origin. Physical blowing agents (PBAs) are substances that are mixed or injected into the thermoplastic during the melting process. PBAs can be added to the polymer in liquid, gaseous, or supercritical form. Examples of gaseous PBAs include carbon dioxide (CO_2_) and nitrogen (N_2_) gas [39], while liquid PBAs include hydrocarbons such as isopentane [40]. CO_2_ has much higher solubility and lower diffusivity in molten polymers than N_2_ and is mostly used in the extrusion foaming of low-density foams. In contrast, N_2_ is more employed in manufacturing high-density foams using the foam injection moulding technique. Hydrocarbons, such as pentane, have higher solubility and lower diffusivity in polymers compared to N_2_ or CO_2_, but their use is being discontinued due to environmental issues and side effects such as toxicity and flammability [41]. A supercritical fluid (SCF) is any substance at a temperature and pressure above its critical point but below the pressure required to compress it into a solid. The main supercritical fluid employed as a PBA in polymer foaming is CO_2_, although N_2_, sometimes in conjunction with CO_2_, is also employed [42,43]. In contrast, chemical blowing agents (CBAs) are chemical substances that are dispersed or dissolved within the polymeric matrix and decompose thermally into non-reacting gases, such as CO_2_ and/or N_2_, during the foaming process. Examples of CBAs include azodicarbonamide [44,45,46,47], N, N-Dinitroso pentamethylene tetramine (also known as H foaming agent) [48,49], sodium bicarbonate (NaHCO_3_) [10], and zinc carbonate (ZnCO_3_) [50]. After the decomposition of the CBA, the formation of a separate gas phase can be instantaneous, or it can take some time depending on the solubility of the gas within the polymer. As the gas released by the CBA decomposition reactions is normally N_2_ or CO_2_, which are also used as PBAs either as gases or in the form of supercritical fluids, it is also necessary to trigger a sudden drop in pressure or an increase in temperature to reach a state of supersaturation of the gas and initiate the foaming process. Compared to PBAs, CBAs present some drawbacks such as their mostly exothermic nature that makes it difficult to control the foaming process and the presence of harmful residues in the foams that can cause allergic reactions, limiting, therefore, the application of the foams [38].

Several review papers have addressed the foaming process [6,38,50,51,52], so the topic will be briefly described.

**Figure 2:**
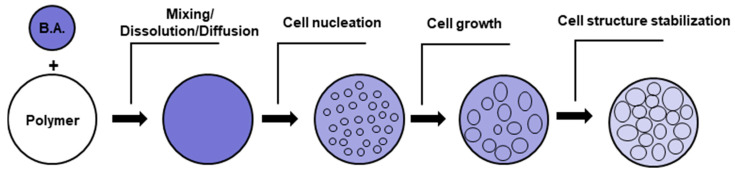
Main steps of polymeric foaming process. BA stands for blowing agent, which could be gas (PBAs) or some compound that could release gas during foaming (CBAs).

In general, the foaming process of thermoplastics consists of the following steps shown in the schematic diagram of Figure 2: (1) Dissolution of the blowing agent within the polymer matrix giving rise to a homogeneous blowing agent/polymer mixture. The blowing agent could be either a gas or supercritical fluid (PBA) or instead it could be some solid compound (CBA) that is mixed with the polymer and subsequently generates gas at elevated temperatures during melting. (2) Formation of small gas bubbles, which are normally called cells, in a process known as cell nucleation, when the mixture becomes super-saturated due to some thermodynamic instability, such as an abrupt pressure drop or an increase in temperature. (3) Growth of the air bubbles or cells. (4) Upon cooling, the cell growth ends due to the vitrification or crystallization of the thermoplastic polymer, which stabilizes the cell morphology. Each step will be explained briefly.

The first step, the formation of the homogeneous polymer/blowing agent mixture, is important in the foaming process. This step depends on the characteristics of both the blowing agent and the polymer. In case the blowing agent is a gas that is added directly to the polymer, its diffusion within the polymer depends on factors such as gas concentration, temperature, pressure, as well as other factors such as the interaction of the gas with the polymer or the polymer free volume [53,54]. The solubility of the gas in the polymer increases with increasing gas concentration, increasing polymer free volume, and increasing pressure, but it decreases with increasing temperature. In the case of melt mixing methods (extrusion or injection moulding) that imply the application of shear rates on the material, the increase in the shear rate promotes a greater alignment of the polymer chains and a consequent decrease in the free volume of the polymer that in turn results in a decrease in the solubility of the gas within the polymer [55].

The second step involves the formation of small nuclei that will give rise to bubbles or cells within the polymer matrix. The formation of these nuclei, or nucleation, is caused by a sudden drop in pressure, or alternatively an increase in temperature, which causes a sudden decrease in the solubility of the blowing agent within the polymer. As the blowing agent is dissolved in appreciable amounts within the polymer, a sudden drop in solubility causes the molten polymer to become supersaturated with gas. To reach a new equilibrium, the gas in excess is forced out of the polymer. Nucleation begins with the formation of small clusters of gas molecules within the polymer [56,57]. The nucleation rate depends on the magnitude of the pressure drop. Larger pressure drops yield larger nucleation rates and cell densities [58].

In the third step, the gas bubbles, or cells, grow as more gas molecules join the clusters of gas molecules that were formed during the cell nucleation process. This process is enhanced by the decrease in the solubility of the gas inside the polymer and is affected by factors that influence the solubility and diffusion of the gas within the polymer, such as temperature and pressure [59]. The viscoelastic properties of the polymer also strongly influence this process as the material is subject to deformations during cell growth [60,61]. The last step involves the stabilization of the foam morphology (cell size and cell distribution), since the continuous cell growth would lead to cell coalescence (fusion of cells) and coarsening (growing of the bigger cells at the expense of the small ones) causing an overall deterioration of the foam properties. Stabilization is carried out by reducing the temperature of the polymer which in turn increases the viscosity of the melt. The viscosity of the melt also increases due to the diffusion of the gas out of the polymer, as the gas acts as a plasticizing agent [62]. Another important factor affecting cell stabilization is the strain hardening. Strain hardening arises from stretching that causes a large-scale alignment of the chain molecules in the stretch direction. Due to strain hardening, the thin sections of the cell walls, created by the stretching of the cells during their growth, become more resistant, which makes their further extension difficult. Thick sections are extended instead, as they were less subject to the phenomenon of strain hardening, giving rise to the phenomenon of self-healing [60,63]. Important polymer properties that affect foam stabilization include the polymer molecular structure, molecular weight distribution, degree of chain branching, and degree of crystallinity [64,65]. Foaming can also be controlled by the inclusion of additive fillers with a high aspect ratio [66]. The main process parameters that affect foaming are the blowing agent concentration, the pressure drop (how much and how it is carried out), and the temperature at which the thermodynamic instability promoting the cell nucleation is triggered [67]. In general, keeping constant the temperature and gas concentration, faster decompression rates yield foams with smaller cell sizes, while foaming at higher temperatures and keeping other factors constant yields foams with larger cell sizes [68].

In addition, some particulate pore-forming substances can act as porogens and can be used to control the nucleation and, in that way, the morphology of the solid foams, by serving as sites for heterogenous nucleation initiation. Many published results demonstrated that nucleation efficiency strongly relies on the particles’ size, shape, and surface properties and their dispersion within the polymer [69,70]. Examples of porogen substances include ammonium bicarbonate [71], sucrose particles [72], hydroxyapatite, nanocellulose and graphene oxide [73], and sodium chloride [74,75], among others.

In polymeric foaming, temperature and pressure conditions must be carefully selected. When using CO_2_ as blowing agent, the polymer should be solid during foaming, to allow for a stable foaming process, but at the same time, the polymer must allow a large solubilization of CO_2_. For this, the saturation of the polymer with CO_2_ normally takes place at temperatures near the glass transition temperature, when considering amorphous polymers, or near the melting point in the case of crystalline polymers. Moreover, when selecting the foaming temperature, it must be considered that a polymer’s glass transition and crystallization temperatures decrease due to the plasticizing effect of CO_2_ [76,77]. The presence of crystals during foaming also affects the final foam structure, as crystal formation not only boosts bubble nucleation but also decreases the CO_2_ amount dissolved in the polymer because CO_2_ can be dissolved only in the amorphous regions [78]. The CO_2_ content also plays an important role in foam expansion, since a low CO_2_ content does not lead to significant foam expansion, while larger CO_2_ contents show intense shrinkage upon ageing. Reignier et al. [79] found a narrow processing window for CO_2_ content, between 7 wt.% and 8.3 wt.%, in PLA foaming. In addition, they found that an increase in pressure enhances cell nucleation, thus leading to foam densities lower than 30 kg/m^3^. Supercritical CO_2_ can also act as a sterilizing agent during the foaming process, which is useful in the preparation of bio-based polymer scaffolds with a controlled pore size for tissue engineering applications [80].

Polymer foams can be characterized by their cell size distribution, expansion ratio (ratio between the unfoamed material density and the foamed material density), cell density (number of cells per unit volume of the foam), and porosity (open cell content). Polymer foams can also be characterized as flexible or rigid foams according to their physical properties and pore morphology. Additionally, and depending on the average cell size, polymer foams can be divided in macrocellular (>100 μm), microcellular (1–100 μm), ultramicrocellular (0.1–1 μm), and nanocellular (0.1–100 nm) [81]. Polymer foams can also be divided in open-cell foams (also known as “strut foams”), interconnected cell foams, and closed-cell foams. In general, open-cell foams show an open-cell content higher than 90% in volume while closed-cell foams have open-cell contents lower than 10% in volume [82]. In open-cell foams, cells do not have walls to confine the air inside them, while in closed-cell foams, the cells are separated from each other and surrounded by cell walls. Cell structure has great repercussions on the mechanical performance and on the thermal and acoustic insulation properties of the foams. In general, open-cell foams offer better absorptive capability and find applications in acoustic, while closed-cell foams possess lower permeability, which provides improved thermal insulation properties [81]. The literature also shows that the thermal insulation of a polymer foam can be improved by changing the foam morphology, particularly by decreasing the foam cell size in the micro-range to restrict air flow within pores and thus reduce the air conductivity [5,83,84]. Decreasing the cell size further to the nano-range can improve thermal radiation conductivity due to thinner cell walls, thus cancelling the effect of reducing the air conductivity by restricting the air flow [5,85]. Mechanical performance can be improved by adjusting the foam morphology. Regarding cell size, some studies have indicated that nanocellular foams tend to show both higher impact resistance and elongation at break, compared to microcellular foams. Regarding cell density, foam polymers with higher cell density usually show higher mechanical performance in terms of tensile strength and Young’s modulus [57].

Several studies have shown that the foam morphology can be adjusted through different strategies, ranging from simply varying the foaming conditions to modifications in the polymer structure, namely in the crystalline structure and in the crystallinity degree of the polymer [86,87,88] and changes in the molecular structure via crosslinking [89], branching [86,90], grafting [89], or chain extending [89,91], all aimed to improve the melt strength. The melt strength is understood as a measure of the extensional viscosity and can be defined as the opposition of the polymer melt to stretching [50]. A higher melt strength improves the cell morphology during the cell growth phase by eliminating cell coalescence and rupture [82]. Some polymers, namely PLA, have low melt strength, which makes the foaming process more difficult due to the mentioned cell coalescence and cell-wall rupture. Besides the above mentioned strategies that can be implemented to increase the melt strength of some polymers and improve their behaviour during the foaming process, several studies showed that the addition of nanofillers promoted a more heterogeneous nucleation process while improving melt strength [92,93,94]. In fact, an increase in melt strength limits the loss of gas during the foaming process, resulting in an increase in the foam density accompanied by a closed-cell structure.

## 3. Foaming Processes

Polymer foams can be obtained by different processing technologies, namely foam extrusion, foam injection moulding, batch processing (i.e., in an autoclave), bead foaming, microwave foaming, or additive manufacturing. Several published review papers have reviewed several polymer foam production processes [39,41,50,51,52,95,96]. This review will provide an overview of these main processes of foaming commonly applied to biodegradable thermoplastic polymers to obtain lightweight parts.

### 3.1. Extrusion Foaming

**Figure 3:**
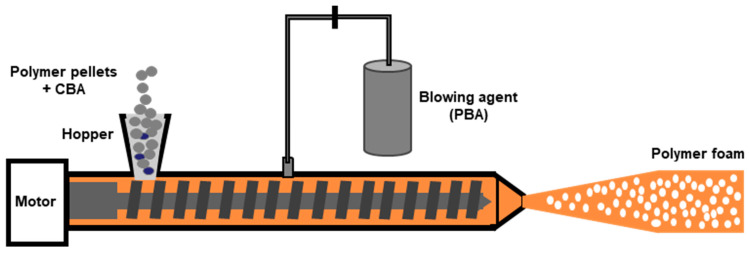
Diagram of extrusion foaming showing the two options for combining the blowing agent and the polymer.

Conventional polymer extrusion is a well-established industrial continuous process used to produce plastics with a defined profile geometry. Extrusion foaming was developed by combining conventional polymer extrusion with a process for dispersing a blowing agent within the melt. The dispersion of a blowing agent within the polymer melt can be achieved by injecting a gas directly into the molten polymer (PBA) or by mixing chemical agents (CBA) that will release the gas during the melting process. However, the use of PBAs (nitrogen or carbon dioxide) is more common [38]. In extrusion foaming, the polymer in the form of pellets is fed into the extruder through the hopper. The blowing agent is either injected at high pressure into the extruder barrel (gaseous PBAs) or is previously mixed with the polymer pellets before the feeding (solid CBAs). The pellets are melted inside the extruder barrel due to the high temperatures and are further mixed with the blowing agent, forming a homogenous mixture of the blowing agent combined with the melted polymer. A representative diagram of the extrusion foaming process is shown in Figure 3.

The melted mixture is conveyed by the screw into the direction of the extruder die. As the polymer/gas melted mixture exits the barrel, the mixture is subjected to ambient conditions and an abrupt pressure drop ensues. Thermodynamic instability ensues due to this pressure drop, which will cause phase separation [97]. Cell nucleation occurs and the formed cells start growing. Foam stabilization soon follows, which depends on the temperature and on the viscoelastic and rheological properties of the polymer [52]. The shape of the extruded foam is given by the geometry of the die and can be further calibrated and cut. The blowing agent concentration, the flow rate, and the die temperature can control the cell density and expansion ratio. Nevertheless, the decompression rate in the extrusion die has been recognized as the key parameter that controls the cell nucleation and determines the cell density, with higher pressure drop rates promoting increased cell densities [98].

An extrusion line that combines two extruders is also common and is called a tandem line. In this process, melting and cooling are performed in separate barrels, which makes it possible to obtain better results [99]. However, tandem lines are more complex and expensive and prone to gas leakage, making the process less attractive [51]. Lee et al. [100] used a tandem foaming extrusion line to manufacture low-density PLA foam sheets. They used the primary extruder to efficiently melt the PLA resins, whereas the secondary extruder was used to effectively cool the melt to maximize the melt strength. The blowing agent iso-butane was injected in the primary extruder at a constant flow rate. Tabatabaei et al. also used tandem extrusion to study the strain-induced crystallization behaviour of PLA during foaming [101]. Wang et al. [102] also used a first extruder to melt the polymer resin and disperse the blowing agent within the melt and a second extruder to further mix and cool of the melt.

In the extrusion foaming of biodegradable polymers, expansion has been achieved mostly with the use of PBAs. CBAs are rarely used for the reasons stated above. Examples of CBAs used and reported in the literature in extrusion foaming include azodicarbonamide, which was used mainly with PLA [103,104], PLA blends with poly(butyleneadipate-co-terephthalate) (PBAT) [105], and PLA composites. Other studies that used CBAs reported a mixture of sodium bicarbonate and citric acid to obtain the foamed material [106,107].

Regarding PBAs, CO_2_ is the most common PBA used. CO_2_ has a plasticizing effect on polymers, which enables the use of lower temperatures to decrease the viscosity and achieve the required melt strength to prevent cell rupture or high open-cell contents [108]. In addition, processing at a lower temperature significantly increases the cell nucleation rate since higher melt viscosity induces higher pressure drop rates [109]. Several studies have demonstrated the dependence of foam density on CO_2_ concentration. Low CO_2_ concentrations resulted in low polymer expansions [110]. On the contrary, higher concentrations of CO_2_ resulted in foams with lower densities [79,111].

The extrusion can largely benefit from the use of supercritical CO_2_. Supercritical CO_2_ can be defined as a fluid state of carbon dioxide where CO_2_ is held at a pressure and temperature above its critical point (critical temperature of 31.0 °C and critical pressure of 7.38 MPa) [95]. Supercritical CO_2_ is very popular in foaming because carbon dioxide is non-toxic, chemically inert, and its supercritical conditions are easily reached. Supercritical CO_2_ acts as a blowing agent during the polymer expansion, which occurs when the polymer exits through the extruder die. Besides the expansion effect, supercritical CO_2_ also acts as a plasticizer agent, improving the extrusion processing by creating conditions that prevent the thermal decomposition of the polymer.

Nofar [92] used extrusion foaming with supercritical CO_2_ as a blowing agent to study the effect of nano-/micro-sized additives in PLA foaming by a single-screw tandem extruder. The supercritical CO_2_ was injected through a syringe pump in the first extruder barrel, which was used to melt the polymer and to deliver a homogeneous polymer–CO_2_ mixture. The second extruder was used to achieve a uniform cooling profile for the mixture before foaming was triggered. Pilla et al. [112] used a single screw extruder to foam a blend of PLA/PBAT with supercritical CO_2_ as a blowing agent. The polymers in pellet form and the filler based on talc were fed into the extruder barrel and were completely melted before reaching the gas injection port, from where the blowing agent was injected into the extrusion barrel. The effect of talc, which was added to promote heterogeneous nucleation, and die temperature on the crystallinity, volume expansion, and cell morphology were studied. They reported that the addition of talc has decreased the average cell size and volume expansion rate and increased the cell density and crystallinity.

### 3.2. Foam Injection Moulding

**Figure 4:**
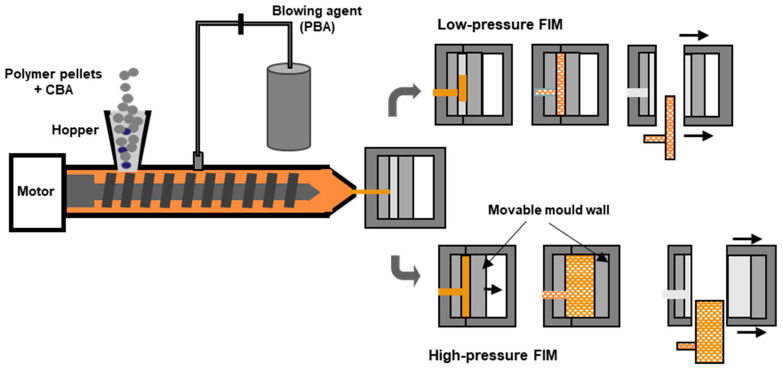
Diagram of the foam injection moulding with the two options for the addition of the blowing agent and the two mould-filling concepts. Adapted with permission from Standau et al. [38].

Like foaming extrusion, foam injection moulding (FIM) is a variant of the conventional injection moulding process. The main differences are the use of a blowing agent and some additional equipment features such as a special nozzle. As in the case of extrusion foaming, the blowing agent can be added jointly with the polymer pellets through the hopper (in the case of CBAs) or directly injected into the molten polymer in the barrel. The screw pushes the gas/polymer mixture along the barrel and injects it into the mould. The foaming process within the mould can occur by two different processes that differ mainly in the pressure inside the mould cavity (see Figure 4). In the case of low-pressure FIM, the mould is just partially filled with the gas/polymer melted mixture, which triggers a sudden drop in the pressure to which the mixture is subjected. This leads to immediate foaming, causing the polymer to expand and occupy the entire mould cavity. In the other variant, so called high-pressure FIM, the mould is first pressurized and then the melt is injected at high pressure into the mould to fill the entire cavity. Afterwards, the mould is partially opened, causing an increase in the mould volume and a drop in the pressure, which triggers, in this way, the foaming and the expansion of the injected molten polymer.

The foaming mechanisms associated with the high-pressure FIM variant have been significantly investigated to understand the mechanism of cell nucleation and growth. The pressure profile inside the mould cavity has been determined as the dominant factor in controlling cell morphology. Using this technique, foams with improved surface quality were obtained mainly due to the elimination of early cell growth at the flow front during the mould-filling step [113]. The rate of mould opening is another process parameter that has been shown to effectively control cell structure [114,115].

The high-pressure FIM enables a better control of the cell nucleation and coalescence, as opposed to low-pressure FIM, in which the control of cell nucleation and growth is more difficult, due to inevitable shearing that arises during the completion of the filling of the mould cavity with foam expansion, yielding less uniform foam structures [116]. The use of a gas counter pressure in the high-pressure FIM prevents the premature foaming during the mould-filling stage, enabling the attainment of higher void fractions and more uniform foam structures [117]. Hou et al. [118] reported a gas-assisted microcellular injection moulding technique by combining microcellular injection moulding with gas-assisted injection moulding. In this technique, due to the high pressure, the amount of polymer melt that is required to completely fill the mould cavity is lower compared with the amount required in the microcellular injection moulding, thus yielding a large reduction in the weight of the foamed part. The high pressure used can also dissolve all the cells that are formed during the mould-filling step. The foaming is triggered by releasing the high pressure, yielding foamed parts with an excellent foam morphology and a compact skin layer.

As previously mentioned, both chemical and physical blowing agents can be used in FIM, although CBAs are seldom used with biopolymers [119]. Few studies were found in the literature regarding the use of CBAs in the injection moulding of biodegradable polymers. The few examples found used Azodicarbonamide [119,120]. The main PBAs used in foam injection moulding include CO_2_ [121,122] and N_2_ [123,124,125,126]. Nitrogen has a higher cell nucleating force, although it shows a lower solubility in polymers compared to carbon dioxide [53]. Supercritical N_2_ can also be used as foaming agent in FIM [127,128]. The microcellular injection moulding process known as Mucell^®^ moulding [129], patented by Martini-Vvedensky et al. [130], is an injection moulding technique that mixes the polymer melt with gas at a supercritical state (known as supercritical fluid inert gas, CO_2_ or N_2_) to generate a single-phase polymer/gas solution and to produce lightweight products [127,129]. In addition, by acting as a plasticising agent, supercritical N_2_ decreases the melt viscosity of the polymer, allowing for lower processing temperatures to be used, which is beneficial when using temperature-sensitive materials such as bio-based polymers [131,132]. In general, using N_2_ as blowing agent results in improved cells when compared to CO_2_ due to its low solubility, high diffusivity, and larger nucleation rate. In addition, using the same amount of gas foaming agent, N_2_ induces a higher cell density and higher expansion ratio when compared to CO_2_. Moreover, the injection-moulded parts foamed with the aid of N_2_ shrink less than those foamed with CO_2_ [133]. Thus, this injection moulding foaming technology requires a higher control of several processing conditions, namely mould temperature and mould cavity pressure, which are the key factors determining the surface quality of the foamed part. They will determine both the solid skin layer thickness and foam core characteristics (e.g., cell size and cell density), which regulate the apparent density, weight reduction, and the mechanical properties of the foamed parts [129].

### 3.3. Batch Foaming

**Figure 5:**
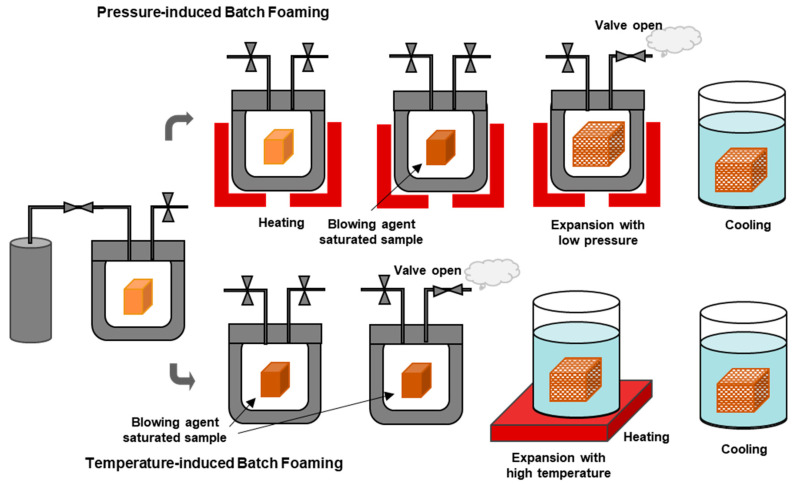
Diagram representing both the pressure-induced batch foaming and the temperature-induced batched foaming processes. Adapted with permission from Standau et al. [38].

Unlike extrusion foaming and foam injection moulding, batch foaming is a discontinuous foam technique where cell nucleation and growth steps are conducted after and separately from the step of the saturation of the polymer with the blowing agent. Another difference is that, unlike extrusion foaming and foam injection moulding, in batch foaming, the polymer to be foamed is in a solid state, contrary to the former two techniques in which the foaming takes place when the polymer is in a molten state. Normally, the cell growth step occurs inside an autoclave, and the foaming process takes several hours to complete. This, combined with the fact that small samples are normally obtained, makes the technique unattractive at an industrial level, with its use being basically reduced to scientific research. This process has two main variants that are distinguished by the way the foaming is triggered, which can be temperature-induced or pressure-induced. In both variants, at first, the polymer sample to be foamed is saturated in a pressurized container or autoclave for a certain period. Afterwards, foaming is induced, depending on the variant, by applying a pressure drop or changing the temperature. Figure 5 shows a schematic comparison between both variants of batch foaming that are discussed next.

#### 3.3.1. Temperature-Induced Batch Foaming

In the case of the temperature-induced variant, the polymer sample is saturated with the blowing agent at high pressure and below its glass transition temperature (Tg). Next, the sample is depressurized and removed from the autoclave vessel. At this stage, the polymer sample does not experience an expansion. The polymer expansion is obtained by immersing the sample in a hot oil [134] or glycerine [135,136] bath at a temperature kept above the T_g_ of the polymer [137]. The foaming is triggered by the huge drop in the solubility of the blowing agent in the polymer due to the increased temperature (above the T_g_), which results in cell nucleation and subsequent cell growth. The cell nucleation and subsequent growth are further induced by the increased mobility of the polymer chains as the polymer becomes softened due to the higher temperature [51]. Increasing the foaming temperature reduces the polymer viscosity, which in turn reduces the resistance to cell growth and results in increased cell size [99]. Finally, the foamed sample is subjected to a cooling step to stabilize its shape.

Temperature-induced batch foaming is seldom used and only a few studies reported the use of this variant. Wang et al. [138] used temperature-induced foaming for PLA following a CO_2_ saturation at 5 MPa for 12 h. The samples were removed from the vessel after a rapid quench of pressure and were quickly transferred to an ultrasonication water bath running at a frequency of 20 kHz, after which the foamed samples were quenched in cold water after foaming. Ultrasonication was introduced at the start of the foaming to achieve cell structure and cell density uniformity in PLA samples with high crystallinity, since high crystallinity usually yields a nonuniform cell nucleation and hampers cell growth when using solid-state foaming. Ultrasonication will be further addressed in Section 3.5. Brütting et al. [139] used temperature-induced batch foaming to produce low-density PLA foamed beads with a density lower than 100 kg/m^3^. The PLA beads were saturated with CO_2_ in an autoclave. After gas sorption, the beads were placed in an oven at 100 °C to induce foaming.

#### 3.3.2. Pressure-Induced Batch Foaming

In the case of the pressure-induced variant, the solid polymer sample is saturated with the blowing agent inside an autoclave vessel at a high pressure and at a defined temperature. Afterwards, the vessel is subjected to a sudden pressure drop, which makes the heated polymer over-saturated with the blowing agent and starts phase separation. In turn, the phase separation triggers cell nucleation and its subsequent growth, which results in the expansion of the polymer sample. In the last step, the sample is cooled so that the stabilization of the foam structure can be achieved [99,140].

Pressure-induced foaming is the most used variant in batch foaming processes. Several processing parameters of the pressure-induced batch foaming process have a strong influence on the final foam properties [141,142]. These include (i) the saturation pressure, at the stage of saturation with the blowing agent, which controls the solubility of the blowing agent; (ii) the temperature, which affects the chain mobility and hence the crystallization behaviour; (iii) the saturation time, which also affects the amount of blowing agent dissolved in the polymer and hence the corresponding plasticizing effect on the polymer; and finally, (iv) the pressure drop, which impacts on the nucleation rate and the overall foaming process [143]. Chen et al. [144] used a constrained foam method to prepare microcellular PLA foams with a wide range of cell structures by tuning saturation pressure in a supercritical CO_2_ solid-state batch foaming process. Their results showed decreased cell wall thickness and increased cell size and cell density with increased saturation pressure. Athanasoulia et al. [145] studied the effect of the depressurization rate on the structure of PLA foams prepared by batch foaming with supercritical CO_2_. Foams with a thin wall pore structure and characterized by anisotropy, with a pore diameter ranging from 100 to 650 μm, were obtained when supercritical CO_2_ was instantaneously depressurized. When a slow depressurization rate was applied instead, foams with highly organized pore structures, with thicker pore walls and pores with diameters raging between 10 and 350 μm, were obtained.

Interestingly, Tammaro et al. [146] developed an innovative pressure vessel to perform batch foaming using multiple conditions in a single foaming experiment. The developed technology consists of a pressure vessel containing several reaction chambers, each one enabling a different set of foaming temperature and pressure drop rates. The pressure vessel was tested using PCL and CO_2_ as a blowing agent in one single test and it confirmed a strong dependency of the foaming process on the foaming temperature. The dependence on the pressure rate was less clear, but a slight decrease in density was observed by increasing the pressure drop rate while fixing the foaming temperature.

Several strategies can be used to improve the properties of polymers during batch foaming. Yan et al. [2] introduced stereo-complex crystallites as an effective modifier to reinforce PLA and improve the cellular morphology, cell uniformity, and increase the expansion ratio at high foaming temperatures. The followed methodology enabled the fabrication of high-expansion PLA foams with improved thermal insulation and compressive performance. Dreier et al. [147] used reactive extrusion on a twin-screw extruder to modify PLA with dicumyl peroxide as a melt strength enhancer and polycarbodiimide as a hydrolysis stabilizer, in order to protect PLA against thermal degradation and hydrolysis and to improve its melt strength during batch foaming. Li et al. [148] introduced long-chain branching structures into PLA by employing soybean oil under the initiation of trace amounts of cyclic peroxide to improve the melt strength of PLA and its crystallization performance during a batch foaming process.

### 3.4. Compression Foaming

**Figure 6:**
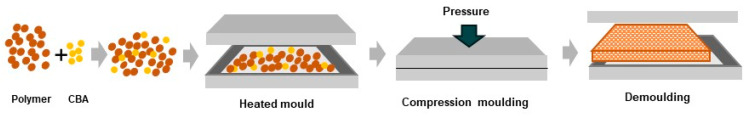
Diagram showing the steps of the compression moulding technique.

Compression moulding foaming can be considered as a batch-type process that is used in the production of thick foams. In this method, a mixture of a polymer and blowing agent (usually a CBA) is initially placed inside a mould. The foaming process is carried out by the decomposition of the blowing agent inside the hot and pressure-closed mould [149]. This process gives rise to a limited expansion rate. Figure 6 shows a representative diagram of this process. To obtain foams with lower densities, a two-step compression moulding process is used. In this two-step process, a pre-expanded foam is first produced with the aid of a CBA. Subsequently, the remainder of the CBA decomposes, leading to foam expansion, roughly 40 times the original size, which completely fills a preheated mould. In the first step, which involves mixing the polymer, additives and blowing agent, a solid sheet is produced, which is then placed inside a mould at a certain temperature and for a certain amount of time to partially decompose the blowing agent. The gas released in the decomposition of the blowing agent dissolves in the polymer due to the high temperature and pressure. Subsequently, after opening the mould, nucleation occurs and the material expands directly out of the mould, giving rise to pre-expanded foam. In the second foaming step, the cell growth step, the pre-expanded foam is placed in a second mould and heated to higher temperatures to complete the decomposition of what remains of the CBA. The foam is then expanded to fill this second mould, which will give the foam its final shape. A rapid cooling is critical to avoid gas escape and subsequent foam collapse [82]. The addition of a nucleation agent, such as talc, can reduced the bulk density of the foam [150].

The foaming process of some aliphatic polyesters is difficult due to their low molecular weight and linear chains, which causes a low melt strength. One way to increase the melt strength of aliphatic polyesters is through crosslinking or branching. Improved foams can be obtained by compression moulding foaming by using crosslinked or branched polymers through use of crosslinking agents and curing co-agents. Using this approach, closed-cell PBS foams with a degradable property were successfully produced using dicumyl peroxide as a crosslinking agent and trimethylolpropane trimethacrylate as a curing co-agent to modify PBS and azodicarbonamide as a blowing agent [151]. The release of azodicarbonamide during the foaming process can result in cell collapse due to heat release. The heat released can be absorbed using endothermic expandable microspheres that prevent cell collapse and enable the tailoring of the cell size of PBS foams [152].

### 3.5. Baking

The bake foaming technique is inspired in the backing technology commonly used in food processing and is specially used in starch-based foams. In this method, a starch–water batter is firstly baked in a heated and closed mould. The water acts both as a plasticizer and as a blowing agent. During baking, the starch granules are gelatinized, yielding a thick paste. The paste expands intensely, as the entrapped water evaporates rapidly due to heating, filling up the mould cavity. The starch foam gradually dries, by releasing the residual water by evaporation, and acquires the shape of the mould. Upon cooling, the foam is demoulded, keeping the shape acquired from the mould [153]. The process is somewhat time consuming, taking between 125 and 300 s to obtain a homogenized and stabilized foam. Furthermore, the amount of material inserted in the mould must be carefully determined in order to obtain an adequate filling of the mould cavity. Bake composition, mould temperature, and baking time are other important process variables. Baked foams typically have a dense outer skin layer with closed-cell and small pore size morphology, due to the rapid loss of water from the foam zone that is most in contact with the hot mould walls, and a less dense and large interior with open-cell and larger pore-size morphology [7,154].

As the composition of starch depends on its origin and type, it is expected that the type of starch used has some kind of influence on the foam properties. Starch foams made of wheat, potato, or tapioca starches have shown superior properties compared to foams made of corn starch [155]. The foam properties can also be affected by the viscosity of the batter, as an increase in the viscosity causes a decrease in the foam expansion. To counteract this effect, a higher amount of batter is needed to ensure the complete mould filling [156]. Fillers, such as fibres, can be added to starch to increase the batter viscosity [153].

### 3.6. Ultrasound-Aided Foaming

The ultrasound foaming technique is not a stand-alone technique of polymer foaming but is rather applied in the context of the batch foaming technique. In this context, the foaming process is enhanced by the application of external ultrasonic vibrations to improve cell interconnection and permeability. This technique can be particularly useful in the field of tissue engineering, namely in the development of scaffolds with open cells without resorting to the use of solvents or leaching methods. These more traditional techniques, such as leaching or freeze-drying, make it possible to control the porosity of the materials but reduce the biocompatibility with living tissues due to the persistence of solvents and leaching residues [157,158]. The batch foaming technique that uses CO_2_ as a blowing agent makes it possible to avoid the use of solvents, but the resulting foams have a closed-cell morphology, which, in view of some biomedical applications, makes them of little use due to their lack of permeability to biological cells or fluids. The application of ultrasound can overcome these issues by breaking the pore walls and creating an open-cell solid-state foam to improve the permeability of the solid-state fabricated foam [159].

**Figure 7:**
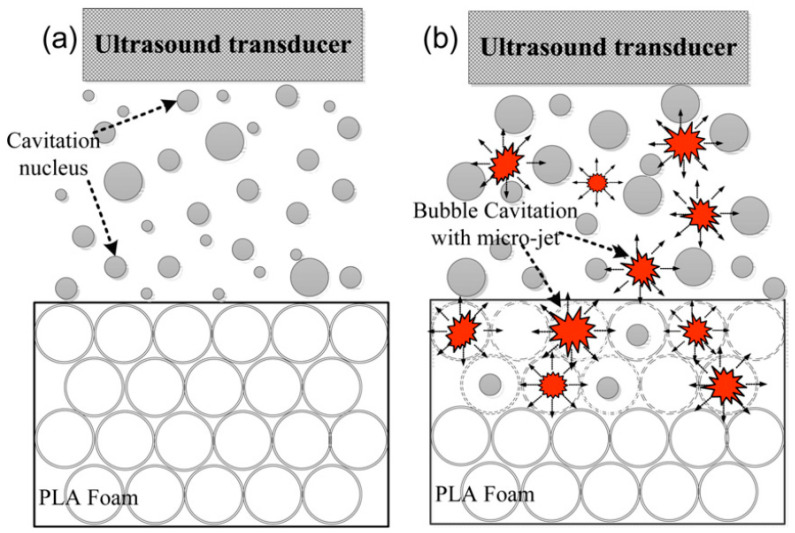
Diagrams of ultrasound-assisted porosity improvement in PLA foams that show the effect of acoustic vibration, micro-jet, and ultrasonic cavitation. (**a**) initial state and (**b**) cell rupture state. Reprinted with permission from Guo et al. [159].

The technique is based on the application of instantaneous energy generated by acoustic vibration, micro-jet, and ultrasonic cavitation to increase the permeability between the closed cells of the polymer foam. The foam is dipped in water and when the foam micro-bubbles are excited by the power ultrasound, they vibrate and can further expand, shrink, or collapse. During the cavitation process, the collapse of the bubbles/cells generates high temperatures and pressures in a very confined space that are sufficient to break the cell walls. This process is enhanced by the micro-jet-induced current shot at high velocity that promotes further cell rupture (see Figure 7) [159]. The ultrasonic step can be applied either at the beginning or after the cell nucleation, and depending on that, cell morphology is greatly affected. If the ultrasonic step is applied at the beginning of the cell nucleation, the ultrasonication leads to the nucleation of a large number of small cells resulting in a higher cell density. On the other hand, if the ultrasonic step takes place after cell nucleation and growth, the ultrasonication results in cell wall rupture, yielding a more open cell structure and interconnected cells [160].

Wang et al. [138] used ultrasonic irradiation on PLA saturated with CO_2_ and showed that ultrasound irradiation when applied at the beginning of the nucleation step results in enhanced cell nucleation, which in turn results in an increased expansion ratio and cell density and improved cell structure uniformity. Commonly used process parameters of the ultrasound foaming technique that affect the foam structure include ultrasound frequency and intensity, ultrasound exposure time, and the temperature of the water bath. The best combination of parameters of the ultrasound treatment to improve the permeability of the cell structure includes high ultrasound power, low ultrasound frequency, and high water temperature [161,162]. The initial foam cell size plays a crucial role in determining the effectiveness of the ultrasound treatment. Previous studies have shown that foams with bigger cell sizes allowed for the obtainment of foams with higher cell interconnectivity after ultrasound treatment [162]. Guo et al. [159] reported the increase in ultrasound power as the main factor to improve cell interconnectivity in PLA foams.

### 3.7. Microwave Foaming

The microwave foaming process can be used to fabricate the foam itself by using water as the blowing agent. Using this approach, Lopez-Gil et al. [163] produced starch-based foams filled with natural reinforcements using water both as the plasticizer and as the blowing agent. Zhao et al. [164] also used water as the blowing agent in PVA foaming by microwave irradiation. By adjusting the microwave intensity and water content, the morphology of the foamed materials could be tailored. Using CO_2_ as a blowing agent instead of water, Sundarram et al. [165] prepared PLA foams by CO_2_ saturation followed by microwave foaming and reported that power and temperature were the main factors affecting the pore morphology in PLA foams.

### 3.8. Foam 3D Printing

The term “additive manufacturing” (AM) in polymer materials refers to a variety of polymer processing technologies, where the parts are built up layer-by-layer from a CAD model. Commercially important techniques for polymer-based materials include powder bed fusion technologies, such as multi-jet fusion (MJF) and selective laser sintering (SLS), where the polymeric material in powder form is deposited as a layer in the printing bed and fused using infrared light (in the case of MJF) or melted/sintered locally using a special laser (in the case of SLS), and material extrusion technologies such as fused deposition modelling (FDM) (also known as fused filament fabrication), where a polymer filament is fed to the printer, melted in a printing head, and deposited on a printing platform [166]. Among these technologies, the extrusion-based technologies are nowadays more used in the production of foamed thermoplastics and will be discussed in more detail.

Briefly, the FDM technique starts with the design of the part to be printed using 3D design software. This digital design is then sliced by a slicing software into a series of layers and transmitted to the printer, which reproduces it layer-by-layer on a printing platform. During printing, a continuous thermoplastic filament is fed to the printer, which is melted in the printing head and deposited as thin lines on the printing platform. The 3D part to be printed is treated as a continuous set of 2D layers, with each one of them being built at a time by moving the printing head horizontally in the x- and y-directions. The movement of the printing platform in the vertical z-direction enables the printing of one layer on the top of the previous one, building up the 3D part layer-by-layer [167]. Based on the geometry of the model part to be fabricated, a support structure may be built up in the same way to support the model material. Just like the model material, the support material is fed into the head of the machine in the form of a filament. Once the model part is completed, the support structures are removed [168]. Printing quality is controlled by printing parameters such as the filament diameter, line width, layer height, infill degree, infill pattern, nozzle temperature, printing speed, cooling speed, and temperature of the printing platform [169]. The FDM technique can also be used to produce polymer foamed structures. The foam structure may be generated during printing, in the same way as extrusion foaming, using variants of the FDM technique, or the FDM technique may be used to fabricate polymer-based multiscale 3D freeform structures that are subsequently gas-foamed in a two-step process. Both methodologies, the in situ foaming of filaments containing a blowing agent and the post-foaming of pre-formed solid structures, will be discussed below.

#### 3.8.1. Foaming during Printing of Filaments Containing Blowing Agent

**Figure 8:**
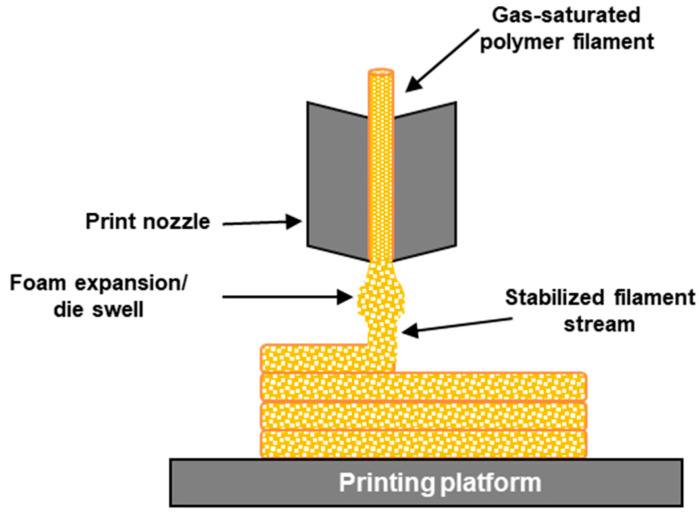
Schematic diagram of print head in foam 3D printing using pre-saturated polymer filaments.

The FDM technique has been used to produce foamed structures using filaments previously saturated with PBAs or incorporating CBAs. In this approach, the foaming takes place during the print stage and is triggered by a pressure drop at elevated temperatures, which leads to thermodynamic instability. The prepared polymer is initially saturated with a blowing agent gas, normally CO_2_, in a high-pressure chamber at a certain temperature and pressure. The gas diffuses into the polymer matrix and a polymer/gas solution equilibrium originates. During printing, this equilibrium is broken by a pressure drop at elevated temperatures and cell nucleation is induced. Further cell growth enables the formation of a foam structure. Foam morphology can be tailored by adjusting certain processing parameters during the gas saturating phase, such as the saturation pressure, and during the printing phase, such as the pressure drop rate, foaming temperature, feed rate, infill ratio, nozzle temperature, and printing speed [170,171,172]. Figure 8 presents a representative diagram of the printing process using CO_2_ pre-saturated polymer filaments. As in extrusion foaming, cell nucleation and growth take place at the nozzle and are prompted by the sudden pressure drop as the filament exits the printing nozzle.

In this AM process, the blowing agent must remain in the polymeric material until the printing stage for the technique to be feasible. Therefore, blowing agents with low solubility in polymers, such as N_2_, present serious difficulties since the gas would be lost even before the filament is heated in the printer. Certain hydrocarbons have high solubility in polymers; however, due to environmental concerns, more environmentally friendly blowing agents have replaced these materials. CO_2_, although not as soluble as some hydrocarbons, presents itself as an alternative to saturate the polymer filaments used in 3D-printed foams. The solubility and permanence until the printing stage of the gas blowing agent within the polymer is influenced by the chemical structure of the polymer [173,174]. For example, the tendency for CO_2_ desorption in PLA could be remedied by increasing PLA’s molecular weight or by increasing the number of branches in the polymer structure. In this way, a material with higher chain entanglements could encapsulate higher amounts of CO_2_ during the saturation phase and gas desorption could be delayed [52,175]. Marascio et al. [176] used foaming during the printing technique by combining AM with supercritical extrusion foaming to manufacture 3D structures with well-controlled porosity. The materials used, PLA as a control, a PLA composite, and a PLA/PCL copolymer, were saturated at high pressure with supercritical CO_2_, which was released during the deposition of the molten filament in the printing platform. The pores’ morphology was tailored by process parameters such as the temperature, nozzle geometry, and printing speed. Higher temperatures enabled a more expanded foam due to increased cell growth after cell nucleation, while the lower temperature tested was shown to be insufficient to enable proper cell growth after cell nucleation. Higher foam expansion was also obtained with increased printing speed due to the induced higher depressurization rate.

Filaments incorporating CBAs were also developed. To improve PLA foamability, Choi et al. [177] employed a reactive extrusion process to increase the molecular weight of PLA by a chain extension reaction in order to improve its rheological properties during extrusion foaming. During extrusion, the PLA was also blended with the CBA azodicarbonamide, yielding a PLA foamable filament filled with the CBA. The filament was subsequently foamed at the CBA decomposition temperature (at 200 °C) by combining extrusion foaming and 3D printing, yielding a dual-pore PLA scaffold with sub-macro-sized pores (10–60 μm) and macro-sized pores (200–300 μm). Process parameters commonly used to tailor foam density and structure include the printing temperature, which is closely connected to the rate of decomposition of the CBA, and flow rate. Studies have shown an increase in the foam density with an increase in the flow rate, while increasing the printing temperature and activating the foaming agent results in an increase in the quantity and size of the bubbles, thus decreasing the foam density [178].

#### 3.8.2. Post-Foaming 3D-Printed Structures

**Figure 9:**
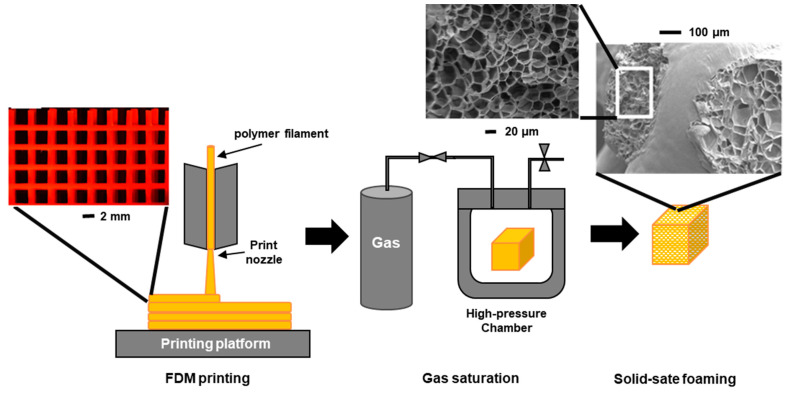
Diagram of the foaming stages using FDM and gas foaming combined technology. Adapted from Hu et al. [179]. Optical image of the 3D-printed sample and the cross-sectional SEM images of the foamed samples adapted with permission from Kakumanu et al. [180].

Foamed materials can also be produced by combining the traditional technique of 3D printing with a later stage of foaming. This last foaming stage involves two steps: a first one in which the printed material is saturated with gas in a high-pressure chamber and a second step, after leaving the high-pressure chamber, in which the material expands due to the sudden pressure drop. Figure 9 displays a schematic diagram of the FDM combined with a later stage of foaming.

Several research groups have combined the FDM and batch foaming techniques to fabricate tissue-engineered scaffolds using PLA [170,181]. Using this approach, Park et al. [182] produced PLA-based multiscale 3D structures through the combination of 3D printing technology with a two-step gas foaming stage to yield foams within the 3D-printed structures for use in thermal insulation. Kakumanu et al. [180] used a combined AM and solid-state foaming approach to obtain a highly porous dual-pore network PLA foam. The first step included the preparation of a polymer template with the appropriate architecture using the additive manufacturing technique. A foam structure with a pore size around 200 µm was obtained in this way. Afterwards, the second porous network with a pore size in the order of tens of microns was obtained by subjecting the obtained template to a solid-state foaming process. Samples were saturated with CO_2_ in a pressure vessel at room temperature. After depressurization, the samples were placed in a glycerine bath at 90 °C to foam, which resulted in reduced spacing between the struts of the first porous network.

Parts formed by the combination of AM and batch foaming generally have a denser skin layer due to the lower concentration of the blowing agent in the vicinity of the filament surface. To remedy this situation, Song et al. [183] used a mixture of PLA and PVA in the production of scaffolds using AM and batch foaming. After foaming, the PVA was removed by solvent etching, yielding a hierarchical macro-/microporous biodegradable scaffold with interconnected pores and without any dense skin layers made of PLA. The use of PVA, as a sacrificial barrier polymeric, combined with PLA can also be used in surface patterning through selective foaming. Using this principle, Loffredo et al. [184] printed a PVA solution as a selective coating on a thin PLA film, which was subsequently exposed to a gas-foaming process. PVA, a well-known gas-barrier polymer, prevented the premature gas loss, thus enabling the effective foaming of the PLA film only on areas where PLA was coated by PVA. After removing the PVA, a bubble-patterned PLA foam was obtained. Sanz-Horta et al. [185] combined multi-material fused deposition modelling with two other complementary techniques, supercritical CO_2_ batch foaming and the breath figures mechanism, to produce well-defined objects with internal and external porosity. The fused deposition modelling was used to make structures that combined PCL and PLA. The supercritical CO_2_ foaming and breath figures mechanism were subsequently used to selectively yield internal (with pore sizes ranging between 80 and 300 µm) and external pores (sizes ranging between 2 and 12 µm) solely in those areas that contained PCL.

Three-dimensional printing enables the production of complex scaffolds with different architecture designs, high fidelity, and different pore size and interconnectivity for high-demand uses, such as the health sector [186,187]. However, the technique has certain limitations with regard to the minimum pore size (a porosity of less than 10 μm is difficult to construct), as the pressure increases sharply as the nozzle diameter decreases, which limits the printing capacity of the printing nozzles [170]. Lepcio et al. [188] employed 3D printing pre-alignment combined with subcritical CO_2_ crystallization towards the growth of highly ordered PLA crystallites, which acted as a template for the development of oriented microporous canals upon foaming in a heated oil bath. The semi-crystalline character of PLA enabled the formation of fine channels, as the amorphous phase allowed for the growth of pores while the crystalline phase served as a template for pore growth.

### 3.9. Bead Foaming

Bead foaming is a foaming technique that is already industrialized on a large scale and is well established in the packaging sector. This technique is mostly suitable for producing low-density foam packages with complex shapes such as the ones used in the packaging of fragile goods during transportation. The parts produced by this technique are basically made of a large number of foamed particles welded together, forming three-dimensional items with densities in the range of 15–120 kg/m^3^ [58]. Normally, these parts have similar properties as the foams obtained by extrusion with comparable densities, such as low thermal conductivity, high acoustical insulation, and good impact energy absorption. The main advantage over foams obtained by extrusion is the production of extremely lightweight and geometrically complex shapes with high dimensional accuracy, which are important features for cushioning fragile items. The most common polymers used in this technique include polystyrene (PS), known as expandable PS (EPS) and used mostly in packaging and building insulation [189], and polypropylene, known as expanded PP (EPP) and used in the automotive sector [190]. Considering the use of biopolymers in bead foaming, PLA has been the subject of particular attention in attempts to replace petroleum-based polymers with bio-based biodegradable materials, with a view of significant sustainability [96,191].

The PLA bead foaming technique is analogous to the one normally used to produce EPS [96]. Bead foaming is usually performed in two steps. The first step of this technique involves the saturation of polymer granules with a blowing agent, normally CO_2_, at a temperature below Tg to make the single foamed beads. In the second step, these single foamed beads are further expanded and subsequently welded or sintered to generate the final part. Usually, this is achieved using a steam chest moulding machine to give the part the desired shape. Foamed beads can be produced using two different methods. In the first, expandable beads are created, which are further pre-expanded. In the second, expanded beads are directly obtained. Expandable beads are polymer particles saturated with a blowing agent (usually pentane) that are expanded in a subsequent step before the sintering or welding process that yields the final foamed product. This enables a more efficient transportation of the unfoamed beads as they occupy less space. Given the fact that the material in a solid state must hold the blowing agent for some long periods of time, only amorphous thermoplastic resins can be employed in this technique. When using semi-crystalline thermoplastics, expanded beads are produced instead, since the storage of a blowing agent within the solid bead is hampered by the existence of crystalline domains [192]. A brief description of the variants of the foamed bead technique follows.

There are several routes to produce the single foamed beads, but only the ones commonly applied to biopolymers will be addressed. As already mentioned, depending on the route followed, expandable (amorphous polymers with Tg higher than room temperature) or already expanded beads (semi-crystalline polymers with Tg lower than room temperature) can be obtained. One route used to obtain expandable beads is the impregnation of micro-granules with the blowing agent in an autoclave [193]. Firstly, the micro-granules are prepared by extrusion. Subsequently, the micro-granules are impregnated with a blowing agent under pressure in a closed vessel, such as an autoclave, at temperatures close to the melting point. In the case of amorphous polymers, the impregnation must be carried out at a temperature below the Tg of the polymer. Other alternatives include charging the particles with the blowing agent inside an autoclave together with a dispersion medium such as water. After the impregnation step, pre-foaming of the expandable beads is achieved in an expansion vessel with hot water or using hot air, yielding low-density expanded beads.

**Figure 10:**
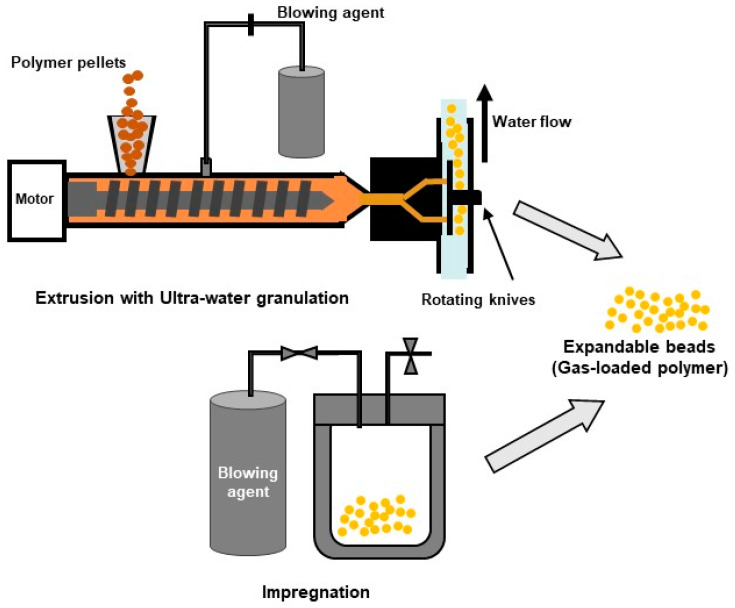
Methods for producing expandable beads.

Another route to obtain expandable beads uses extrusion combined with underwater granulation [194,195]. The melt mixture of the polymer and blowing agent is extruded into a pressurized water chamber and granulated by rotating knives. Foaming during extrusion is prevented by maintaining the water pressure above the vapour pressure of the blowing agent, in this way trapping the blowing agent within the solidifying polymer during cooling. The gas-loaded expandable beads are subsequently pre-foamed at low pressure by hot vapour, triggering cell nucleation and further growth and allowing for the release of the trapped gas, yielding low-density expanded beads at the end. This method presents several advantages compared to the impregnation method such as the dosing of the blowing agent and additives into the melt and the fact that it is a continuous method [38,58]. Figure 10 illustrates the methods mentioned in this review for producing expandable beads.

The parts from bead foams are manufactured by welding together the previous formed expanded beads using a steam chest moulding machine. For this, the surface of the expanded beads is softened or melted, using hot steam at high pressure, inside a specially designed mould. The beads’ cohesion is achieved through the entanglement of polymer chains between neighbouring beads [196]. The mould is responsible for the shape of the final part, enabling the production of geometrically complex parts with very low density. A brief description of the method follows. More details can be found in the review of Raps et al. [58]. The process takes place within a mould, which contains steam nozzles or valves in the walls to enable the steam to enter the foam chamber during the process. With the mould closed, the foam chamber is filled with foamed beads using special injectors.

**Figure 11:**
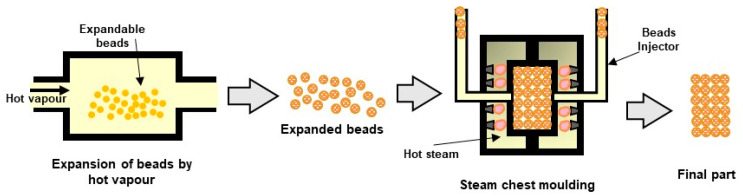
Diagram representing the process for producing foamed parts using expandable beads.

The heating of the mould takes place by flowing hot steam through the mould. The air between the beads is also removed by the steam that flows through the chamber by keeping all valves open to allow for the purging of the air. The hot steam flows through the mould in various directions by opening and closing the valves accordingly to ensure a homogeneous temperature distribution. The steaming step is crucial to enable the creation of a foam skin and guaranty uniform welding throughout the whole part. Afterwards, the mould is cooled down to avoid further expansion of the foam beads once the part is out of the mould. This can be performed by flowing cold water through the mould. Finally, the mould is opened to allow for the exit of the final part. Figure 11 illustrates the process of producing a foamed part using expandable beads.

The production of expanded PLA bead foams presents some challenges compared to expanded polystyrene, especially the sintering step to join the foamed PLA beads. Amorphous PLA grades and semi-crystalline PLA grades with a lower melting point (around 150–160 °C) can be used to produce bead foam parts by sintering, contrary to PLA grades with a high melting point (around 170–180 °C), which can be pre-foamed but the foamed beads cannot be thermally sintered due to the high melting point [197]. Bead foams of semi-crystalline PLA grades are generally more difficult to sinter, compared to bead foams of amorphous PLA grades, because semi-crystalline regions hamper the melting process and consequently hinder the sintering of the foamed beads [197]. Some strategies were developed to improve the sintering of expanded PLA beads, namely the use of expanded PLA beads with a double-crystal melting peak structure (i.e., the crystals with a high melting temperature and crystals with a low melting temperature) [198]. The high-temperature melting peak crystals generated during the isothermal gas-saturation step can be used to preserve the part geometry during sintering in the steam chest moulding step, which uses low-temperature melting peak crystals that melt during the sintering stage. The steam temperature is in between the low-temperature melting peak and the new high-temperature melting peak, which was created during the gas-saturation step. The steam temperature is sufficient to melt the low-temperature melting peak crystals, which is enough to enable the sintering of the PLA beads but not the high-temperature melting peak crystals, which remain unmolten, enabling in this way the preservation of the original geometry of the beads. Using this approach, the effect of temperature, CO_2_ pressure, and saturation time on the double crystal melting peak generation and on the subsequent foaming behaviour was investigated [199]. PLA bead foams with average cell sizes ranging from 350 nm to 15 μm were obtained. The measured tensile properties of the steam chest-moulded foamed parts, namely a tensile strength of nearly 0.5 MPa and Young’s modulus of nearly 27 MPa, were comparable to those of available expandable polypropylene foams.

The blend of PLA with other bio-based polymers has been explored to overcome some drawbacks of PLA, such as brittleness, poor processability and low melt strength, and to improve its final properties. PLA/Polybutylene adipate-co-terephthalate (PBAT) and PLA/Poly(butylene succinate-co-butylene adipate) (PBSA) blends have been recently reported in the context of bead foaming [200]. The blends were previously prepared by an internal mixer and by twin-screw extrusion. In turn, the bead foams were prepared by impregnating the samples with supercritical CO_2_ at a high pressure in an autoclave chamber. After saturation with the blowing agent, the pressure was quickly released and the chamber was afterwards cooled in a water bath. The method showed that different microcellular bead foam morphologies could be obtained by using blends with several PLA grades differing in molecular weight as well as different processing techniques.

The use of PVA in the manufacturing of bead foams by the techniques applied to other polymers presents difficulties due to the high melting point (226 °C) and semi-crystalline nature of PVA. These difficulties encouraged the development of a different approach to achieve the production of PVA bead foams. In this approach, the expandable PVA beads are produced by polar solvent plasticization, followed by a solid-state supercritical CO_2_ impregnation and a surface plasticization by coating with a polar solvent. Foaming is triggered by exposing the PVA beads to microwave radiation, which heats the polar solvent at the surface and in internal sections of the beads [201]. This method enables the expansion and the sintering of the beads in a single step, yielding complex-shaped PVA bead foam objects with excellent adherence between the beads and good elasticity.

## 4. Biodegradable Polymers in Foaming

**Figure 12:**
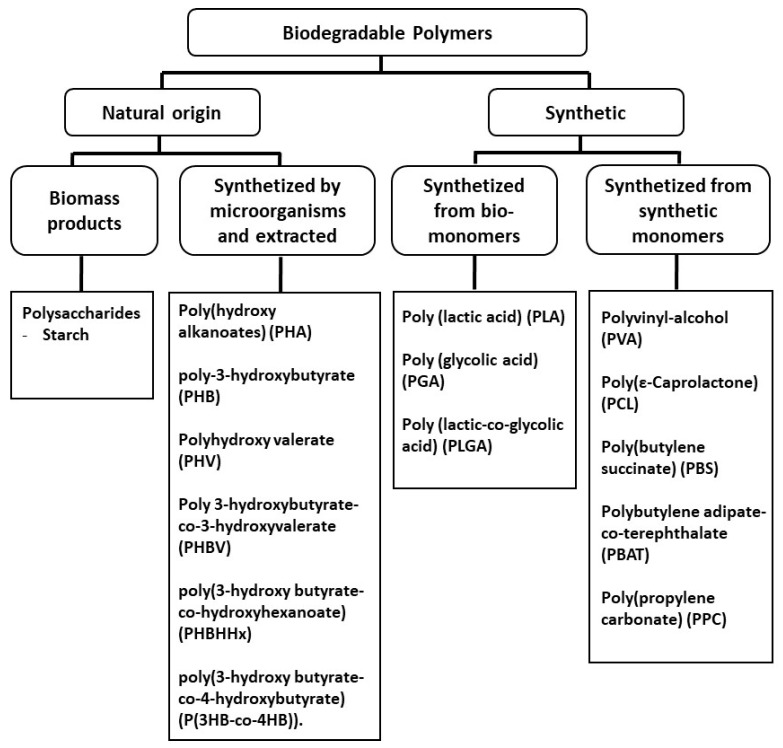
Classification of different biodegradable polymers used as matrix in foaming by thermal or melt-based technologies. Adapted from Gurunathan et al. [203] and Rajeshkumar et al. [204].

According to IUPAC recommendations, biopolymers are defined as macromolecules (including proteins, nucleic acids, and polysaccharides) formed by living organisms. However, the term biopolymer is frequently applied to classify polymers that are either bio-based, biodegradable, or show both properties, which creates some confusion and leads to misrepresentation of the information. The prefix *bio* is normally perceived by laypeople as something that is biodegradable or is completely natural based. However, some polymers made from renewable bio-based raw materials, such as biomass-derived polyethylene (PE), show high resistance to biodegradation. On the other hand, some synthetic polymers derived from petrochemical sources, such as polycaprolactone, are considered biodegradable [202]. Biodegradation is this context is understood as the chemical conversion process of materials into natural substances, such as water, carbon dioxide, and compost, by microorganisms available in the environment without the need of artificial artefacts, such as additives, to accelerate the process [30]. In this review, only the foaming of biodegradable polymers will be addressed. Figure 12 shows the classification of different biodegradable polymers, some of which are bio-based while others are not.

Biodegradable polymers are largely different in terms of raw material source, synthesis and chemical structure [205]. They can be broadly divided between polymers of natural origin and synthetic polymers. Some synthetic polymers use bio-derived monomers, such as PLA, while others, such as PCL, use petrochemical-derived monomers. The first and second groups (Figure 12) are based on polymers synthesized naturally by living organisms such as plants, algae, or microorganisms. It includes polysaccharides, protein-based polymers, and polymers obtained from microorganisms. The polymers are extracted and purified and are further used without modification of its chemical structure, or they are slightly modified to address specific applications, such as the conversion of starch into thermoplastic starch [153]. From the first group, starch is commonly used in foaming. The third group includes polymers synthetized from bio-derived monomers. They are not natural polymers, because they are synthetized and not extracted from plants or microorganisms, although the raw material is bio-based, meaning that the original monomers come from biomass. The last group includes biodegradable polymers synthetized from petrochemical-derived monomers. Of the mentioned polymers, these polymers from the last group are the only ones that are not bio-based, since the raw materials do not come from biomass. Of all these biodegradable polymers, PLA is the most used in foaming [206,207,208,209,210,211]. Other biodegradable polymers that have been applied in foaming include poly(vinyl alcohol) [212,213], poly(propylene carbonate) [214,215], poly(butylene succinate) [216,217], poly(ε-caprolactone) [17,218,219,220,221,222], Poly(butylene adipate-co-terephthalate) [223], poly(ethylene oxide) [224], poly(hydroxyalkanoates) [225,226], poly (lactic-co-glycolic acid) [211,221], and poly(ethylene glycol) [213,227]. In the following sub-sections, a brief description of the uses of some of these biodegradable polymers in foaming will be detailed. Each section will also include the foaming of composite materials, in which the polymer in discussion is used as the matrix. Blends of various polymers, including their composites with various fillers, will be discussed in the polymer blends section.

### 4.1. Poly (Lactic Acid) (PLA)

PLA is a renewable and a biodegradable aliphatic thermoplastic polyester that is synthetized from lactic acid (2-hydroxypropanoic acid). This monomer exists in two enantiomeric forms, L-lactic acid and D-lactic acid, that are mainly obtained by the bacterial fermentation of starch-rich plants, such as cassava, sugarcane, corn, and sugar beet. PLA can be synthetized using several polymerization routes including the ring opening polymerization of lactide (the cyclic dimer of the basic repeating unit), polycondensation of lactic acid, and enzymatic polymerization. Detailed information on the synthesis of PLA can be found in the review papers from Van Wouwe et al. [228] and Campos de França et al. [33]. Due to the existence of the two enantiomeric forms, three stereoisomers (poly(L-lactic acid) (PLLA), poly(D-lactic acid) (PDLA), and poly (DL-lactic acid or Meso-lactide)) can be obtained. Poly(DL-lactic acid) can be obtained also from the reaction of racemic lactide. PLA’s overall properties depend on the proportion of the enantiomers, especially the crystallinity, the melting temperature, and the glass transition temperature [229]. PLA’s melting and glass transition temperatures decrease when the PLLA content in the PLA decreases [230]. When D-lactic acid content is above 10%, PLA becomes fully amorphous, while PLA with L-lactic acid content higher than 90% tends to be crystalline [231]. In addition, some studies have demonstrated the influence of the D-lactide content in the compostability of PLA foams, with foams with higher D-lactide content showing faster compost rates [232]. PLA has properties comparable to those of petrochemical-derived polymers, such as polypropylene (PP), polyethylene (PE), and polystyrene (PS). These include good mechanical strength, modulus, stiffness, stability, and transparency [229]. PLA can be processed using conventional technologies such as extrusion, injection moulding, blown film thermoforming, and fibre spinning and has found a wide field of applications, namely in packaging, cutlery, textiles, and agriculture [229]. In addition, its foams can be produced by conventional manufacturing methods, such as extrusion foaming and foam injection moulding. Several additives can be added to PLA to improve the flame retardancy properties of PLA foams and broaden its fields of application [233,234,235,236]. Milovanovic et al. [237] published a concise overview of the main developments in the processing of neat PLA using CO_2_ under elevated temperatures that covers the last decade. Table 1 shows some usages of PLA in foaming processing.

**Figure 13:**
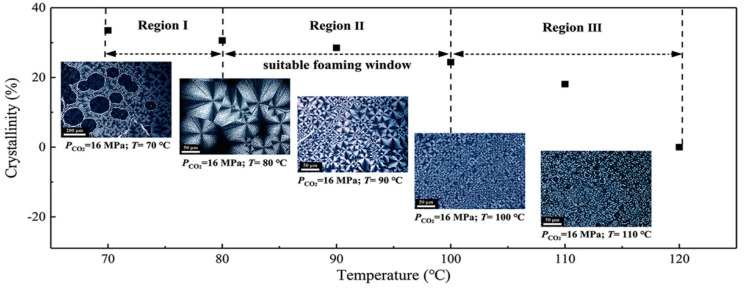
Foaming windows according to the crystallinity and crystal structure dependence on temperature. The inserts show polarized micrographs of CO_2_-induced isothermal crystallization in PLA samples using different annealing temperatures. Region I, first micrograph scale bar 200 µm and Region II and Region III micrographs scale bars at 50 µm. Adapted with permission from Yang et al. [209].

It has been demonstrated that the foaming conditions, such as foaming temperature and time, blowing agent concentration, and saturation time, greatly affect the crystallinity, density, and dimensional stability of PLA foams [238]. Particularly, the dissolved gas used in foaming has great influence on the crystallization and melting behaviours of PLA [239,240]. Among the gases commonly used in PLA foaming, CO_2_ is much more soluble in PLA compared to N_2_ [39,76]. Dissolved CO_2_ increases PLA’s crystallization rate, while N_2_ has a neutral impact on PLA’s crystallization rate due to its low solubility in PLA [117]. In addition, the dissolved gas has a plasticizing effect, influencing the crystallization, the melting, and the glass transition temperatures, lowering them with increasing CO_2_ pressure [76,77,238]. Figure 13 illustrates the relationship between crystal morphology and temperature and the corresponding suitable foaming window. The plot includes polarized optical micrographs of PLA samples annealed at different temperatures under 16 MPa for 30 min. At temperatures below 100 °C, the photographs show spherulites with concentric bands and a pattern in the form of a Maltese cross. Under the effect of CO_2_ plasticization and high temperature, a disordered crystal morphology is observed due to increased chain mobility, which hampers the regular arrangement of the molecular chains, which in turn results in a disordered crystal morphology at 110 °C and no polarizing optical effect at 120 °C. Region II represents the suitable foaming window (foaming temperature between 80 °C and 100 °C), with crystallinity between 30% and 25%. The formed ring-banded spherulites became more packed at higher temperatures and the interface between the crystalline and amorphous regions may act as heterogeneous nucleating sites during depressurization, enabling higher volume expansion ratios and the formation of a uniform cell morphology [209].

Despite the potential of PLA in the preparation of foams, the use of this material presents some difficulties. The low melt strength and the crystallization behaviour of PLA are regarded as the main drawbacks in the preparation of foams with a large expansion ratio and uniform cell diameter due to cell coalescence and cell-wall rupture, resulting in poor foamability [241,242]. In addition, low melt strength is also responsible for the loss of gas during foam expansion, resulting in foam shrinkage. Several strategies were developed to improve the PLA melt strength, including the use of chain extenders to create a branched molecular structure [107,243,244,245,246], monomer composition [247,248,249], the blending with other polymers [250,251], and the addition of additives or fillers [92,252,253]. Hou et al. [254] published a feature article that reviews the recent advances to improve the performance and the cell structure of PLA foams.

The improvement in crystallization kinetics during foaming can also overcome the PLA low melt strength and increase the expansion capacity of PLA by reducing the loss of gas and cell coalescence, although the excess in crystallinity can hamper foam expansion due to higher stiffness and a lower ability to dissolve gas [255,256,257]. The formed crystalline zones can also improve the melt strength by acting as physical entanglement locations that strengthen the support of the cell walls and enhance the modulus and strength of the foams [258]. In addition, the presence of crystals within the material has the same effect as additives or fillers by serving as nucleating agents. The crystallization kinetics of PLA can be improved by using additives that can act as crystal-nucleating agents. Crystal-nucleating agents have the effect of increasing the nucleation density and decreasing the crystals’ size while improving the crystallinity of the material and accelerating its crystallization kinetics [259]. Commonly used additives for that purpose, including talc [139,259], phenylphosphonic acid zinc salt [260], zinc citrate [261], D-Mannitol [262], cellulose nanocrystals [72,263,264], cellulose nanofibers [265], lignocellulosic fibre or particles [266,267,268], rice husk [269], biochar [270], clay platelets [271], organoclay [8], and graphene oxide [272,273], have been shown to be effective in improving foaming, by reducing average cell size and increasing cell density.

Chen et al. [260] developed a methodology to prepare high-volume expansion ratio microcellular PLA foams using phenylphosphonic acid zinc salt as a crystallization nucleating agent. The results showed that the addition of the nucleating agent improved the crystallinity and crystallization temperature of unfoamed PLA. In addition, the nucleating agent played an important role in improving the cellular morphology of PLA foams, yielding a cell density of 1.0 × 10^11^ cells/cm^3^ with a cell size around 1.9 μm. Haham et al. [270] studied the ability of biochar in assisting the PLA foaming through a supercritical CO_2_ batch process. The source and concentration of biochar has been shown to have an effect on the foam morphology, with 0.25 wt.% of biochar particles proving to be an effective nucleating agent by increasing the cell density up to four orders of magnitude and reducing the average cell size compared to the neat PLA foams 10-fold. Higher amounts of biochar resulted in particle aggregation, which in turn resulted in heterogeneous foam densities. Tang et al. [274] prepared low-density PLLA microcellular foams by using dibenzoyl hydrazide sebacate to induce crystallization. The nucleating agent enhanced the crystallinity by providing a high number of crystallization nucleation sites and in this way improved the melt strength of PLLA during foaming. The large number of cell nucleation sites, provided by the large number of crystallization sites, also restricted the growth of PLLA cells. The density of PLLA microcellular foam with 1 wt.% of the nucleating agent was improved by two orders of magnitude, while the foaming expansion ratio was improved ten-fold compared to neat PLLA foam.

Although PLA has mechanical properties comparable to those of the most common petroleum-derived polyolefins, it nevertheless presents some drawbacks, such as low toughness and impact strength, high brittleness, and poor performance at high temperature and humidity, that limit its applicability in certain industrial areas. One strategy that has been adopted to improve some of these negative aspects of PLA is the reinforcement of PLA with additives, especially additives of natural origin and that are biodegradable, such as natural fibres, so as not to lose the biodegradable characteristic of PLA. In addition, the use of these low-cost additives makes it possible to reduce the cost besides altering the properties of PLA foams in the attempt to expand the use of biodegradable PLA in the packaging, cushioning, and construction (thermal and sound insulation) industries. Mort et al. [275] explored the impact of ground coffee chaff and rice hulls on the physical properties of PLA foams. The PLA composites were prepared by extrusion and then foamed by extrusion with the aid of a CBA or supercritical CO_2_. They reported that the addition of 10 wt.% rice hull or 5 wt.% ground coffee chaff did not produce a significant improvement in density compared to the neat PLA foam. In the same study, the filler/PLA composite foams showed remarkable mechanical properties with only 5 wt.% content resulting in the doubling of the compressive modulus, compared to the neat PLA foam. Hassan et al. [276] produced kenaf fibre-reinforced PLA foams by extrusion using azodicarbonamide as a foaming agent. The PLA/kenaf (80/20 wt.%) composite foams exhibit the highest tensile strength (9.3 MPa) and the lowest density (1.11 g/cm^3^) when the lowest blowing agent content was used. Zhang et al. [22] studied the effects of short shot size, cotton fibres, and lubricant on the crystallinity and foaming behaviour of PLA. They reported that the overall crystallinity of the injection-moulded unfoamed and foamed samples was relatively low due to the fast processing cycle and the low crystallization rate of PLA, although with increasing amounts of cotton fibre, the crystallinity slightly increased. In the same study, the foam morphologies are improved when a lower short shot filling condition was employed compared to the fully filled samples, although with the continuous decrease of the shot size and the increase in the amount of cotton fibre, the cell uniformity worsened, which deteriorated the overall mechanical properties.

Certain additives may be included in the PLA matrix to provide certain special properties such as antimicrobial activity [211,277] or provide electromagnetic interference (EMI) shielding [3,278,279]. Table 2 shows some usages of additives in PLA foaming. Regarding the EMI shielding effect, Wu et al. [278] prepared lightweight and biodegradable PLLA/PDLA/carbon nanotube (CNT) composites with an efficient EMI shielding effect through a melt blending method that were subsequently foamed using a batch foaming process aided by supercritical CO_2_. They reported that the shielding of the foams was dominated by absorption phenomena due to the microcellular morphology of the foams and that as the foam expansion ratio increased, the EMI shielding effect gradually decreased due to a reduced amount of CNT per unit volume. Kuang et al. [279] developed an oriented segregated structure under pressure-driven flow in PLA/tetramethylenedicarboxylic dibenzoylhydrazide (TMC-306)@Ni-CNT composites, with PLA/TMC-306 as the polymer matrix and Ni-CNT as the conductive segregated filler. They reported increased complex viscosity of composites with increasing Ni-CNT content, which affected the flow orientation during the pressure-driven flow and the pore growth during the ensuing supercritical CO_2_ foaming process. The composite foams exhibited a high electrical conductivity of 7.58 ×10^−2^ S/cm and a high EMI shielding effectiveness of 25.2 dB at a low Ni-CNTs content of 0.805 vol%. Regarding antimicrobial activity, certain plant extracts can be incorporated in the PLA matrix to provide antimicrobial activity aiming at applications in food packaging. Rojas et al. [277] studied the application of supercritical CO_2_ foaming technology in view of the development of PLA nanocomposite foams functionalized with cinnamaldehyde as an antimicrobial agent. In a two-step approach, they first foamed the PLA filled with organo-modified montmorillonite cloisite nanoclay using a batch foaming process assisted by supercritical CO_2_. The second step involved the impregnation of the obtained PLA nanocomposite foams with the antimicrobial agent cinnamaldehyde also assisted by supercritical CO_2_. They reported PLA foam samples with homogenous microcellular closed-cell morphology with average cell size ranging between 20.1 and 23.4 µm. The presence of the nanoclay in PLA and the inclusion of the antimicrobial agent increased the foam’s cell density and slightly decreased the average cell size. The impregnation of PLA with certain compounds using supercritical CO_2_ takes advantage of the affinities of this solvent for non-polar and lyophilic compounds. Moreover, supercritical CO_2_ shows high diffusion in polymers, acting as a molecular lubricant and enhancing its chain mobility. By inducing polymer swelling, the increased free volume of the polymer matrix enables the incorporation of compounds that are soluble in supercritical CO_2_ [211].

The addition of fillers or other additives to PLA not only harms its compatibility with other polymer matrices but also changes the purity of the polymer, jeopardizing the biodegradation and recycling possibilities of the material. Other strategies than the addition of external nucleation agents could be used to boost cell nucleation. Long et al. [78] explored the viability of homogeneous crystal nuclei to improve and control cell nucleation and growth in PLLA foams without the addition of external nucleating agents. Amorphous PLLA was annealed at temperatures close to its glass transition temperature to favour the local alignment of polymer chains into nanosized aggregates, which they called homogeneous crystal nuclei. The results showed that these homogeneous crystal nuclei strongly affected the foam morphology by acting as heterogeneous nucleation sites for cell nucleation and growth.

The linear molecular structure of PLA together with its weak crystallization ability and low molecular weight are disadvantages that hinder the application of PLA in the foaming field. Chain extension and branching are commonly used methods to increase PLA’s molecular weight and improve PLA’s foaming behaviour [280,281]. The chain extension reactions through the formation of ramifications and/or crosslinking structures makes it possible to increase the molecular weight of the polymer and thus also its viscosity, allowing for the improvement in the melt strength in order to reduce the loss of the blowing agent gas and prevent cell coalescence during foaming. Furthermore, chain extension has the additional objective of modifying the crystallization behaviour of PLA, allowing for the development of crystalline regions, whose interfaces with amorphous regions will serve as heterogeneous nucleation sites [282]. The combined effects of higher nucleation and higher melt strength allow for the formation of foams with a higher volume expansion rate and higher cell density. Venkatesan et al. [283] studied the efficacy in improving the elongational viscosity and foamability of PLA of two food-grade multifunctional epoxies’ chain extenders with low and high epoxy equivalent weights. Both chain extenders were shown to be effective in branching PLA and in increasing its viscosity and molecular weight and decreasing its crystallinity with increasing chain extender amount, due to chain entanglements. The PLA branched foams showed an eight-fold expansion ratio with a high void fraction (up to approximately 85%) and a more uniform cell morphology compared to unfoamed PLA. Li et al. [284] investigated the rheological, crystallization, and foaming properties of a long-chain branched PLA produced by the insertion of cyclic organic peroxide into linear L-PLA through reactive extrusion. They found that the foaming performance of the modified PLA was largely improved due to the enhanced crystallization properties and melt strength. The addition of 0.5 wt.% of cyclic organic peroxide increased the nucleation efficiency to 74.5% and raised both the crystallinity by 15 times and the expansion ratio by 50 times. Li et al. [285] developed a strategy for the production of open-cell PLA-based foams by using a stereocomplexation mechanism between PLLA and a newly synthesized star-shaped PDLA. The addition of the star-shaped PDLA with eight arms to PLLA prompted the formation of stereocomplex PLA, which improved the crystallization and the melt strength. The microcellular foaming of PLLA/star-shaped PDLA yielded open-cell foams with high expansion ratios and high porosity.

The positions of the functional groups on the chain extenders and branching co-reagents determine the distribution of the branched chains along the polymer backbone. If the segments between neighbouring branched chains are long enough to be stretched, there is a slight increase in viscosity and an improvement in melt strength due to the entanglement of the PLA chains. However, if the segments between neighbouring branches in polymer chains are too short to be stretched, an excessive and non-wanted increase in the viscoelasticity is observed, leading to increased difficulties in the processing of such long-chain branched PLA grades. Dual-functional 4-vinylbenzyl glycidyl ether (VBGE) can be used as a branching co-reagent with cyclic peroxide to prepare long-chain branched PLA with tailored structures [286]. VBGE can be grafted onto the PLA chain through the vinyl group, or its terminal epoxy group can react with the carboxyl groups of the PLA chain, meaning that each VBGE molecule can only introduce a branched chain of the same length as the original PLA in the PLA chain. The crystallization behaviour and the melt strength are greatly improved while the viscosity increases only slightly, leading to both improved processing flowability and foamability.

**Table 1:** Processing conditions and main outcomes of studies that have been reported for PLA-based foamed materials.

Objectives	Foaming Conditions	Key Features	Ref.
Investigate the influence of foaming temperature and foaming time on the crystallization behaviour and cell morphology of PLLA and Poly(L-lactic acid)/Poly(D-lactic acid) (PLLA/PDLA) foams.	The solid-state foaming using CO_2_ was carried out in a high-pressure vessel. After saturation, the saturated specimens were transferred to a glycerol oil bath to obtain microcellular foams. The foamed structure was fixed by quenching the foams in cold water.	PLLA and PLLA/PDLA blends were totally amorphous. CO_2_ saturation promoted the formation of the mesomorphic structure in PLLA and PLLA/PDLA blends. The foaming process induced the formation in situ of PLA homocrystal (HC) and PLA stereocomplex crystallites (SCs) in PLLA and PLLA/PDLA. The in situ formed PLA SC could stabilize cell structure and suppress cell coalescence, which facilitated the volume expansion of PLLA/PDLA foams.	[257]
Effects of compressed CO_2_ and cotton fibres on the crystallization and foaming behaviours of PLA.	Batch foaming using CO_2_ as foaming agent carried out in a high-pressure vessel. After the saturation, the vessel pressure was quickly released to trigger foaming and the chamber with the sample was immediately dipped into a cold-water bath to freeze the foam structures.	CO_2_ saturation pressure, temperature, and fibre content significantly affected the crystallinity and foaming behaviours of PLA. A low CO_2_ pressure generated nonuniform foam and a large unfoamed area due to too high crystallinity with a close-packed structure. An intermediate pressure generated a fine cell structure due to the occurrence of numerous less closely packed crystals that served as cell nucleating agents. A high CO_2_ pressure also led to a uniform cell structure but with larger cell sizes due to cell deterioration. The morphology of cells was improved by the addition of low contents of cotton fibres due to transcrystals surrounding the fibres.	[256]
Improve the hydrophilicity and foaming behaviour of PLA by blending with poly (ethylene glycol) (PEG).	Batch foaming using CO_2_ as foaming agent in a high-pressure autoclave. After gas saturation, the autoclave was quickly depressurized, which triggered foaming. After foaming, the specimens were cooled in ice water.	The introduction of PEG improved the foaming behaviour of PLA and promoted the formation of open cells through the reduction in the PLA matrix strength. The obtained PLA/PEG scaffolds exhibited a high expansion ratio, high open-cell content, and super-hydrophilicity.	[287]
Fabricate oriented microcellular PLLA materials using solid hot drawing technology. Investigate the influence of orientation and foaming process on melting and crystallization behaviour and cellular morphology of PLLA foams.	Foaming is performed at a temperature slightly higher than the glass transition temperature to prevent the damage of the oriented structure. PLLA was saturated with CO_2_ using high pressure in an autoclave at 80 °C. Foam was triggered by depressurization to ambient pressure. The foam was then cooled to room temperature.	Highly ordered microfibrils are arranged in the stretching direction, inducing the formation of a dense and aligned shish-kebab-like structure, which enhanced crystallization and provided more sites for cell nucleation. The low-temperature supercritical CO_2_ foaming process induced the oriented PLLA to form a shish-kebab-like crystal structure, improving the overall mechanical properties.	[288]
Improve the heat deflection temperature of PLA foams by annealing.	Single-screw extrusion foaming. Isopentane encapsulated in expandable microspheres and azodicarbonamide as foaming agents. Foam samples were annealed in a hot air oven.	D-lactide content affected the crystallinity of the foam structures. Annealing was shown to be effective in inducing cold crystallization. Lower D-lactide content resulted in a higher degree of crystallinity.	[289]
Study the effect of CO_2_ on crystalline nucleation and spherulite growth of PLA crystals.	CO_2_ saturation in a high-pressure chamber. Isothermal crystallization of PLA underwent for four hours. After that, the sample underwent pressure quenching and was cooled down.	The crystalline nucleation at high temperature and spherulite growth rate at low temperature controlled the crystallization behaviour of PLA under CO_2_. The crystallization kinetics and crystallization morphology of PLA were influenced by the increased chain mobility, the decreased molecular chain density, and the weakened interchain interaction due to dissolved CO_2_.	[290]
Modify the molecular weight, molecular chain structure, the crystallization, and rheological behaviours of PLA using an epoxy-based chain extension method to improve the foamability of PLA.	Batch foaming in a high-pressure vessel using supercritical CO_2_ as a blowing agent. CO_2_ saturation at high temperature. Foaming triggered by pressure drop.	The changes in crystallization and in the rheological properties showed an influence on the foaming behaviour. Cell density of modified PLA increased by nearly 4-fold with increasing chain extension.	[272]
Addition of chemical modifiers (dicumyl peroxide and multifunctional epoxide) to change the rheological behaviour of PLA and improve its foamability in a foam extrusion process.	Foam extrusion using a tandem extrusion line; 8 wt.% CO_2_ was injected as the blowing agent in the twin-screw extruder.	The modifications in PLA structure led to an increase in melt strength that resulted in a more uniform cell morphology and an improved compression strength. Peroxide-modified PLA showed the highest expansion with a foam density of 32 kg/m3. The foamed peroxide-modified PLA doubled the compression strength compared to neat PLA foam even at a density 30% lower.	[20]
Study the long-chain branched PLA structure prepared by UV-induced reaction extrusion with trimethylolpropane triacrylate on the cell morphologies of PLA foams.	The foams were produced by batch foaming in a high-pressure vessel using CO_2_ as a blowing agent. Samples were saturated at high pressure in the heated vessel. After saturation, foaming was triggered by a sudden pressure drop, which was followed by cooling to room temperature.	The modified PLA displayed higher complex viscosity and a higher melting point under super critical CO_2_. Crystal nucleation also improved with the long-chain branching structure. Long-chain branched PLA possesses better foaming behaviour at a high temperature and high pressure with improved cell morphology and reduced coalescence, no collapse, and uniform cell distribution originating in nanocells, while other samples showed microcells.	[243]
Effect of back pressure on the morphology and on the mechanical properties of PLA foams.	Foam injection moulding.	By increasing the back pressure, the percentage of the blowing agent inside the injection chamber is smaller, and therefore foaming is less effective.	[291]
Study the operating conditions of extrusion foaming assisted by supercritical CO_2_ in the production of PLA foams.	Foam extrusion using supercritical CO_2_ as a blowing agent.	The temperatures before and inside the die were the most important parameters that influenced the foam properties. Die temperature between 109 and 112 °C induces low crystallinity and promotes large and open cells. Die temperature below 107 °C induces higher levels of crystallinity resulting in closed cells.	[292]

**Table 2:** Principal fillers or reinforcing agents that have been applied to improve the foamability of PLA or the overall properties of PLA-based foams and main outcomes.

Filler	Objectives/Applications	Foaming Conditions	Main Achievements	Ref.
Graphite	Improve the electromagnetic interference (EMI) shielding effect	Foam injection moulding using N_2_ as blowing agent.	Foaming led to nanographite reorientation, which dramatically improved the electrical conductivity (by six orders of magnitude) of the microcellular PLA/graphite nanocomposite foam compared to the unfoamed material. A microcellular PLA/graphite foam, with a thickness of 2 mm and a density of 0.7 g.cm^3^ a, shows a total EMI shielding effectiveness of up to 45 dB.	[3]
Carbon black (CB) and carbon nanotubes (CNTs)	Study of the synergistic effect of carbonaceous fillers on the electrical conductivity of PLA foams.	Foaming in an autoclave with supercritical CO_2_ as a blowing agent. Saturation with CO_2_ at 0 °C for 12 h. Samples were foamed by immersion in a water bath at 75 °C for 30 s. Finally, the foamed samples were quenched in a 0 °C water bath.	CB particles and CNTs are loosely entangled with each other in the PLA matrix, with no synergistic effect. The electrical conductivity of the CB/CNT/PLA composite is in between those of the CB/PLA and CNT/PLA composites. After foaming, the CB/CNT/PLA composite foam exhibits the synergistic effect of fillers due to the formation of PLA cells with an unbroken wall structure, which is favourable for the establishment of conductive filler networks with fewer defects, resulting in better electrical conductivity than both the CB/PLA and CNT/PLA composite foams.	[293]
Wood Flour	Study the effect of a chain extender on the crystallization behaviour of the PLA/wood flour composites and on the cell morphology of the composite foams.	Batch foaming process in a high-pressure vessel. Samples saturated with CO_2_ at 180 °C and high pressure. Foaming was triggered by sudden pressure drop.	Incorporation of the chain extender improved the melt elasticity and decreased the crystallization rate and final crystallinity of the PLA/wood flour composites. A finer and more uniform cell structure and a much higher expansion ratio was observed in composite foams with increasing chain extender content.	[294]
Cellulosic fibre	Study the crystallization behaviour of PLA/cellulosic fibre composite foams produced using foam injection moulding.	Foam injection moulding with N_2_ as blowing agent.	Cellulosic fibres acted as crystal-nucleating agents, increasing the crystallization temperature and the crystallinity. A finer and more uniform cell morphology was achieved in the cellulosic fibre composite foams compared to neat PLA foams.	[295]
Pulp fibre	Investigate the effect of chain extension, fibre reinforcement, and blowing agent type on the viscosity behaviour and foam morphology of pulp fibre-reinforced PLA composites.	Extrusion foaming using CO_2_ and isobutane as blowing agents.	Isobutane produces foams with a smoother surface and better dimensional stability compared to CO_2_. Isobutane yielded a narrower cell size distribution compared to CO_2_. The addition of fibres reduced the viscosity of the chain-extended PLA.	[19]
Carbon nanotubes (CNTs)	Use CNTs to increase the melt viscoelasticity and foamability of PLA and prepare PLA-based nanocomposite foams.	Batch foaming using supercritical CO_2_ in a high-pressure autoclave. Composite samples saturated at 170 °C and 15 MPa for 2 h. After cooling, foaming was triggered by sudden pressure drop.	The incorporation of CNTs in PLA had a distinct reinforcement influence on melt viscoelasticity. Biodegradable PLA/CNT nanocomposite foam showed a high volume expansion ratio of 49.6 times.	[296]
Calcium phosphate-based glass particulate	Fabrication and characterization of highly porous (up to 91%) composite foams for bone tissue engineering.	Solid-state foaming using high-pressure CO_2_ in an autoclave. CO_2_ saturation during 3 days at 2.4 MPa. Foaming was triggered by an abrupt temperature rise in an oven at 80 °C.	The porous composite systems showed improved elastic modulus and compressive strength as well as well-interconnected macropores (~ 78% open pores at 30 vol.% of filler) compared to neat PLA foam. The pore size of the composite foams decreased with increasing filler content from an average of 920 µm for neat PLA foam to 190 µm for PLA with 30 wt% of filler.	[297]
Cellulose nanofibers (CNFs)	Addition of CNFs and study the processing conditions of foaming extrusion to accelerate the kinetic crystallization of nanocomposites.	Twin-screw extrusion with CO_2_ as a blowing agent. A static mixer was used before the die to improve the dissolution of the gas.	The extrusion induced the reduction in the molecular weight of PLA in the range from 20 to 28% due to the hydrolysis of the ester bond. Samples containing 1.5 wt.% of CNFs exhibited the highest foam expansion, while samples containing 2.0 wt.% of CNFs exhibited the most uniform cell distribution.	[298]
Expanded graphite (EG) nanoplatelets	Improve the foamability of PLA melt through a twin-screw extrusion process by using different aspect ratios and loadings of EG.	Twin-screw extrusion with CFA azodicarbonamide as a blowing agent.	EG improved the melt strength and elasticity and prevented the diffusion of gas molecules from the matrix. The addition of EG yielded microcellular foams with higher void content and cell density and a higher uniformity in cell distribution within the matrix.	[69]

### 4.2. Poly(ε-Caprolactone) (PCL)

PCL is a semi-crystalline, petrochemical-based, and completely biodegradable aliphatic polyester composed of hexanoate repeating units [34]. PCL is obtained by the ring opening polymerization of ε-caprolactone with the aid of a catalyst. A detailed description of the synthesis methods and catalysts used can be found in the review by Labet et al. [34]. PCL has a low melting point (around 65 °C) and a very low glass transition temperature (about −60 °C). It is easy to process by extrusion, injection moulding, film blowing, and melt-spinning. In addition, it shows good biocompatibility, hence the significant interest of its use in tissue engineering. However, its rather low mechanical strength and poor thermal properties narrow its potential in some specific uses for the health sector [299]. Table 3 shows some usages of PCL in foaming processing. PCL foams have potential applications in tissue engineering due to the possibility of the manufacture of foams with interconnected pores to promote cell proliferation and vascularization. These foams or scaffolds with potential applications in tissue engineering can be formed using several techniques, although 3D printing and supercritical foaming have become more interesting due to the absence of organic solvents, whose residues may remain in the foamed material and hinder its use in tissue engineering applications. Foaming with supercritical CO_2_ has attracted a special interest due to a combination of the easy tailoring of the pore structure, through processing conditions, and high compatibility with biological tissues as CO_2_ is non-toxic and chemically inert. This method produces low-density foams (lower limit about 0.2 g/cm^3^, corresponding to a porosity of 80%) when using a temperature range from 30 to 65 °C and a pressure between 10 and 20 MPa [300]. The combination of supercritical CO_2_ foaming with the use of leaching methods with particulate porogens and sacrificial polymers can produce scaffolds with more open and well-defined porosity with narrow pore size distribution, although an extra step is required to remove the porogen by solvent (water) or thermal leaching [71,74]. Polymer leaching is basically based on blending a water-soluble polymer, such as poly(ethylene oxide) (PEO) or poly(vinyl alcohol) (PVA) that will serve as the sacrificial polymer, with the scaffold building material, in this case PCL. The porous structure is then formed by removing the sacrificial polymer with water in a process known as leaching. This technique was applied by Huang et al. [301] in the fabrication of 3D-fibrillated interconnected porous PCL scaffolds by the microcellular injection moulding of PCL/PEO blends and using the water-soluble PEO as the sacrificial polymer. During foam injection moulding, the incorporated PEO smoothed the processing of PCL by decreasing its viscosity, and after leaching, the porosity and interconnectivity of the PCL scaffolds was significantly improved. A similar polymer leaching step to remove sacrificial PEO was used by Hou et al. [302] after the batch foaming of PCL/PEO blends by supercritical CO_2_. They reported an increase in pore density and a decrease in the pore size as PEO content increased, and after the polymer leaching process, highly interconnected pore morphologies were obtained. Li et al. [303] combined supercritical CO_2_ foaming with particle leaching to produce PCL/cellulose nanofiber composite foams with highly interconnected pores. NaHCO_3_, used as a chemical blowing agent, as a heterogeneous nucleating agent, and as a porogen, provided more CO_2_ for cell nucleation and growth. The presence of cellulose nanofibers, acting as a heterogeneous nucleating agent, enhanced the nucleation efficiency but reduced the cell growth rate due to hydrogen bonding and the mechanical entanglement among individual nanofibers that resulted in a rigid network. The highly interconnected porous structure obtained had an open-cell content of 82%, as well as a cell size of 15.8 μm and cell density of 6.3 × 10^7^ cells/cm^3^. Although CO_2_ is generally used under supercritical conditions, Duarte et al. [14] evaluated the possibility of using CO_2_ under subcritical conditions (5.0 MPa and 45 °C) to prepare 3D PCL scaffolds for bone regeneration. The reported foams showed 73–99% porosity with a pore size ranging between 164 and 882 μm and 79–99% pore interconnectivity.

Bone tissue engineering seeks to produce structures that mimic the natural structure of bone in order to try to reproduce the microenvironment of the extracellular matrix of natural bones. Porosity should be at least 65% but ideally around 90% [18,304]. Studies have shown that large pores with a diameter greater than 100 μm are useful for stem cell adhesion, proliferation, and migration, whereas small pores with a diameter ranging from 1 to 50 μm are useful for transporting nutrients and waste within the scaffold [219]. In addition, an interconnected porosity to allow for the vascularization and diffusion of nutrients, besides an adequate pore size to promote cell adhesion, is an essential characteristic when considering tissue engineering applications [18,305]. The pore size range most suitable for cell culture depends on the material and the type of cell, but for the case of bone generation, the suitable pore size is between 100 and 400 μm [306]. A two-step depressurization rate can be used to increase the range of pore sizes in the scaffolds, where a slower depressurization step at a long foaming time that enables the formation of large pores is followed by a fast depressurization step that triggers the development of new small pores and the additional growth of those that previously existed [307]. This two-step depressurization foaming process using supercritical CO_2_ was used by Chen et al. [219] in the production of PCL scaffolds that yielded large pores (over 100 μm), that formed in the slow depressurization step and coalesced during the holding stage, and small pores (below 40 μm) nucleated in the fast depressurization step.

Generally, foams with poor pore interconnectivity are obtained with supercritical CO_2_ foaming due to the fact that the force of the expanding gas is not sufficient to surpass the force of the polymer matrix [303]. Pore interconnectivity can be further improved by advanced plasticizers such as Eugenol, which has interesting bioactive properties such as antioxidant, antimicrobial, and anti-inflammatory activities that can be useful in tissue engineering applications [308]. In an effort to modulate the porous structure of PCL scaffolds, Song et al. [309] have studied the relationship between PCL rheological and crystallization properties and their scaffold final structure and showed that an early and fast crystallization facilitates the solidification of the porous structure by eliminating the growth and rupture of pores, while on the other hand, the decrease in viscosity is advantageous for the deformation of pores during their growth and rupture.

Supercritical CO_2_ foaming results in foams formed by a porous inner core surrounded by a non-porous skin layer. The non-porous skin layer is the result of the fast diffusion of the dissolved gas out of the sample boundaries. However, an entirely porous structure can be achieved by combining the supercritical CO_2_ foaming with the breath figures technique [310]. This technique consists in the immersion of the foam samples in an organic solvent in the presence of a humid atmosphere. In the first stage, the interfacial temperature decreases due to the evaporation of the thin layer of solvent deposited on the polymer surface, which in turn leads to the condensation of water droplets on that polymer surface. The subsequent evaporation of these water droplets from the surface of the polymer is what gives rise to the formation of porosity in the outer layer.

Interesting is the use of supercritical CO_2_ not only to generate the foams but also to impregnate them with certain agents with a view of potential applications. Garcia-Casas et al. [218] reported the foaming and impregnation of PCL with quercetin using a supercritical CO_2_ batch foaming technique. PCL was previously physically mixed with Quercetin before being placed inside the foaming apparatus where it was saturated with CO_2_, at a maximum pressure of 30 MPa and using temperatures ranging between 35 and 60 °C, and then foamed under batch conditions by subsequent depressurization. The obtained foams showed a heterogeneous porosity with a nonuniform dispersion of quercetin throughout the sample, and higher pore densities and smaller pore sizes were obtained with a lower temperature and a higher pressure and depressurization rate (35 °C, 300 bar, and 20 MPa min^−1^). Campardelli et al. [311] developed PCL porous patches for drug delivery using CO_2_ to foam and impregnate the polymer with Nimesulide, a well-known anti-inflammatory medicine with good solubility in supercritical CO_2_. The foaming of PCL and its impregnation with Nimesulide were carried out simultaneously in a one-step process inside an autoclave. Foams impregnated with 35 wt.%. of Nimesulide with circular and uniform pores were generated at 17.0 MPa and at 35 °C. Using higher foaming temperatures did not yield foaming. Kravanja et al. [312] prepared PCL-based biodegradable scaffolds incorporating bioactive calcium phosphate ceramic hydroxyapatite and different amounts of Chitosan as a biocompatible and antimicrobial agent by using supercritical CO_2_ batch foaming. Foaming was triggered by a pressure drop of the high-pressurized autoclave, yielding scaffolds with improved mechanical properties and high porosity. Other studies using the same procedure of combining foaming and impregnation were reported for mesoglycan [313], thymol [314], 5-fluorouracil, nicotinamide and triflusal [315], Ketoprofen [71], Simvastatin [316], natural compounds extracted from *Patagonian usnea* lichen [317], Copaiba oleoresin as a promising bioactive agent to control Aedes aegypti proliferation [318], and an antibacterial agent based on Usnea lethariiformis extract [319].

**Table 3:** Processing conditions and main outcomes of studies that have been reported for PCL foams.

Objectives	Foaming Conditions	Key Features	Ref.
Produce PCL-based porous scaffolds with improved osteoconductive and osteoinductive properties using PCL, silk fibroin, and nano-hydroxyapatite (nHA).	PCL, fibroin, and nHA were mixed and inserted in a high-pressure stainless-steel vessel and pressurized with CO_2_ at supercritical conditions. Foaming was triggered by depressurizing to ambient pressure.	Obtention of solid scaffolds with 67–70% porosity. The incorporation of fibroin and nHA in the scaffolds increased the compressive modulus, cellular adhesion, and calcium deposition. The implanted constructs induced endochondral bone formation and revealed the synergistic effect of silk fibroin and nHA on the bone repair extent.	[320]
Evaluate the effect of soaking time on the preparation of PCL scaffolds by supercritical CO_2_ foaming.	PCL was pressurized with CO_2_ in a stainless-steel autoclave at 39 °C using different soaking periods. Foaming was triggered by depressurization of the system to atmospheric pressure.	Longer soaking times enabled higher quantities of CO_2_ to be dissolved in the polymeric matrix, resulting in more homogeneous scaffolds with a higher density of pores with lower sizes and a higher degree of pore interconnection.	[16]
Evaluate the effects of foaming conditions, namely foaming temperature, pressure, soaking time, and depressurization rate, on the pore structure of PCL foams.	Batch foaming using supercritical CO_2_ in a stainless-steel high-pressure autoclave	Increasing soaking time decreases the pore size distribution. Astride the melting point (Tm) at a given CO_2_ pressure, the PCL shifted from solid-state to melt-state, which led to a broad pore size distribution above Tm and a dense and small pore morphology with narrow pore size distribution below Tm. Lowering the depressurization rate induces pores with higher and broader size distribution.	[321]
Investigate the effect of processing conditions such as CO_2_ pressure, ratios of the PCL polymers with different molecular weight, and amount of added hydroxyapatite (HA) nanoparticles as filler on the scaffold properties.	Supercritical CO_2_ batch foaming in a high-pressure tank.	Porosity increased and average pore size decreased with increasing saturation pressure. Adding HA nanoparticles reinforced the mechanical properties but decreased both the porosity and the average pore size. Incrementing the ratio of the lower-molecular-weight PCL to the higher-molecular-weight PCL in the scaffolds resulted in less uniform and larger pores.	[322]
Investigate the influence of foaming conditions such as saturation pressure, temperature, and time on the resulting foam’s properties. Investigate the application of various pore-forming substances, such as cellulose, carboxymethylcellulose, hydroxyapatite, and graphene oxide in the properties of foams.	Batch foaming process using supercritical CO_2_ in a high-pressure autoclave.	Decreasing the saturation pressure, temperature, and time results in structures with higher crystallinity. Decreasing the saturation time and pressure leads to a narrow pore distribution. The addition of a porogen unit results in an increase in the density of the nucleation sites and degree of crystallinity and a decrease in the pore size compared to the foam made of neat PCL. An increase in concentration of Hydroxyapatite grains at the microscale results in an increase in the pore diameter and a decrease in the pore density, while the opposite effect was obtained with Hydroxyapatite grains at the nanoscale.	[70]
Study the effects of hydroxyapatite (HA) and halloysite nanotubes (HNTs) on the rheological behaviour, mechanical properties, and microstructure of PCL composite scaffolds.	Extrusion foaming using supercritical N_2_ and poly(ethylene oxide) (PEO) as a sacrificial material followed by water leaching of PEO.	PCL/HNT scaffolds showed lower average pore size compared to PCL/HA scaffolds due to higher viscosity and stronger nucleation effect caused by the smaller size and higher aspect ratio of HNTs. Mechanical performance of PCL/HNT scaffolds was higher compared to PCL/HA scaffolds with the same filler content.	[323]
Fabricate interconnected porous PCL tissue engineering scaffolds by microcellular injection moulding.	Microcellular injection moulding, combining supercritical CO_2_ as PBA and sodium bicarbonate as CBA, followed by particulate leaching.	Sodium bicarbonate used both as a CBA and as a porogen improved pore interconnectivity. Scaffolds with higher porosity showed lower mechanical properties.	[324]
Preparation of 3D PCL-based foam scaffolds combined with beta-tricalcium phosphate and dexamethasone as bioactive agents.	The 3D foams were obtained at 5 MPa and 45 °C and dense CO_2_ was used as the foaming agent without using supercritical conditions.	Foams showed a pore size range of 164–882 µm, 73–99% porosity, and 79–99% interconnectivity. Feasibility of using dense CO_2_ to produce in one step a porous matrix loaded with active agents aiming at new injectable systems for in situ foaming.	[14]

### 4.3. Poly(Butylene Succinate) (PBS)

PBS is a biodegradable aliphatic polyester that is synthetized mostly by the polycondensation of 1,4-butanediol with succinic acid, both currently obtained from renewable feedstocks. There are several grades of PBS, differing in the monomeric unit, that are produced by using different diols and diacids and hence show different properties and degradation rates. PBS is a semi-crystalline polymer that has a glass transition temperature of −45 to −10 °C and a melting point ranging between 90 and 120 °C [11,325,326]. In addition, PBS is easy to process, has good chemical and thermal resistance, and has mechanical properties similar to those of polypropylene and polyethylene [202,326,327]. Different techniques can be used for processing thermoplastic PBS, such as extrusion, injection moulding, blow moulding, and fibre spinning, resulting in applications that include electronics, cutlery, packaging, and civil applications. However, the foaming of PBS is rather limited by its linear molecular chains and low molecular weight that results in poor melt strength [328]. Table 4 shows some usages of PBS in foaming processing. The melt viscosity and the melt strength of PBS can be improved by adding Carboxyl-ended polyester and solid epoxy to PBS through crosslinking reactions using crosslinking agent dicumyl peroxide and crosslinking co-agent trimethylolpropane trimethacrylate, enabling the production of foams [329]. Another strategy to increase the melt strength of PBS consists in the incorporation of ionic groups into polymer chains that enable the formation of physical crosslinking sites. The reversible physical crosslinkages are formed when ionic groups aggregate, restricting the chain mobility and consequently rising the viscosity [330,331]. Ru et al. [330] synthetized PBS ionomers containing different contents of aromatic cationic groups by the condensation polymerization of succinic acid and 1,4-butanediol in the presence of Kalium salt of 10H-phenoxaphosphine-2,8-dicarboxylic acid,10-hydroxy-,2,8-dihydroxyethyl ester,10-oxide, a rigid ionic group. The PBS phosphorus-containing ionomers showed a significant increase in the melt strength due to physical cross linking that was caused by the phosphorus-containing ionic group aggregation, and their foams, prepared via a supercritical CO_2_ batch foaming method, showed about a 100% closed-cell structure and high orientation of cells.

Following another strategy, Zhou et al. [332] improved the melt strength of PBS by melt-blending it with vinyltriethyl silane as graft material and benzyl peroxide as an initiator. The obtained silane graft-crosslinked PBS copolyester materials showed improved tensile strength, elongation at break, and melt strength compared to linear PBS. PBS with a ramified or semi-reticulated molecular structure will generate a finer cell structure than PBS with a linear molecular structure due to higher viscosity combined with strain hardening behaviour, which will limit cell coalescence and will generate foams with a homogenous cell structure [333]. Other reported strategies to prepare chain-extended PBS include use of a chain extender having multiple epoxy groups based on a styrene-acrylic oligomer and 2-ethyl-4-methylimidazole as an accelerant [4,334,335] and epoxy groups based on ethylene-glycidyl methacrylate copolymer [336], triglycidyl isocyanurate [335] and dicumyl peroxide as a crosslinking agent, and an aliphatic polyisocyanate as a branching agent [44].

**Figure 14:**
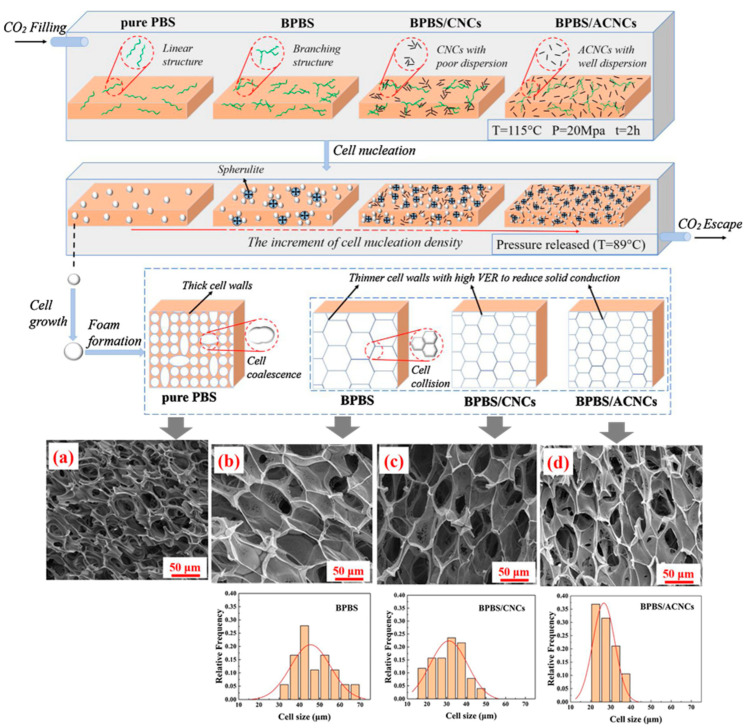
Diagram showing the foaming sequence, SEM images of obtained foams, and cell size distribution of several PBS systems. (**a**) neat PBS; (**b**) BPBS; (**c**) BPBS/CNCs; (**d**) BPBS/ACNCs. Adapted with permission from Yin et al. [4].

Besides chain extension and branching, several additives, such as organic montmorillonite (OMMT) [216], cellulose nanocrystals (CNC) [4,15], carbon black (CB) [217], halloysite nanotubes [337], carbon nanotubes [338], carbon fibres [339], or chitin nanocrystals [340], can also be employed to improve the melt strength and at the same time serve as nucleating agents. Zhou et al. [216] employed OMMT to improve the crystallization behaviour of chain-extended PBS and at the same time act as a nucleating agent during foaming. The crystallization temperature and crystallinity of PBS increased by 4 °C and about 2%, respectively, due to the added filler. Chen et al. [217] fabricated high-strength, lightweight, and electrically conductive PBS/CB nanocomposite foams with densities ranging between 0.107 and 0.344 g/cm^3^ by a solid-state supercritical CO_2_ foaming process. The addition of CB enhanced the rate of crystallization, improved the thermal stability, and increased the viscosity of PBS/CB nanocomposites. In addition, the foamed composites showed a higher cell density, a lower average cell size, and a narrower cell size distribution as compared to neat PBS foams. Fu et al. [340] added chitin nanocrystals (ChNCs) treated with sulfuric acid to PBS. The PBS/ChNC nanocomposites showed improved melt viscoelasticity, crystallization behaviour, and thermal stability. They also reported changes at the level of the pore structure, with the pore strut changing from thin to thick and finally into a wall with increasing ChNC content. Yin et al. [4] fabricated branched PBS/cellulose nanocrystal nanocomposite foams for thermal insulation applications using supercritical CO_2_ as a blowing agent. PBS was first modified by an epoxy-based chain extender to improve the crystallization behaviour, the rheological properties, and the foamability of PBS. In addition, to improve the compatibility between the CNC and PBS, the surface of the CNC was modified by acetylation yielding acetylated cellulose nanocrystals (ACNCs). The added cellulose nanocrystals showed a heterogeneous nucleation effect by improving the cell density. Figure 14 shows a diagram that depicts the foaming sequence of the several PBS systems used.

Using branched PBS, heterogeneous cell nucleation occurred at the interface between the spherulite and amorphous phases. The inclusion of nanocellulose nanocrystals generated additional heterogeneous cell nucleation sites at the interface between branched PBS (BPBS) and the cellulose nanocrystals. The SEM images show poor cell morphology for neat PBS due to its low melt strength that caused cell rupture and coalescence. The branching of PBS improved the melt behaviour and thus reduced the cell rupture and yielded cells with thin walls and a polygonal cell morphology. The cellulose nanocrystals promoted the formation of cells with lower cell sizes and lower cell size distribution, especially the acetylated cellulose nanocrystals.

PBS foams with complex cellular structure can be generated using supercritical CO_2_ batch foaming, by following a two-step depressurization method [15,326]. The two-step depressurization method is used to create a bimodal cell structure by allowing cells nucleated and stabilized in the first step to continue to grow during the second depressurization step, during which new cells are formed. The cells formed in the second step will have less time to grow and therefore will have a smaller size.

PBS can be used to improve the foamability of other biodegradable thermoplastic polymers by blending. The blend composition must be chosen considering improvements in crystallinity, in cell nucleation, or in the solubility and in the diffusivity of the blowing agent. For this purpose, PBS was blended with thermoplastic gelatine (TPG) by melt mixing and the blend was subsequently batch foamed with supercritical CO_2_ [341]. PBS decreased the melt viscosity and increased not only the CO_2_ diffusivity but also the thermal stability of TPG, yielding blend foams with a higher cell density and lower average cell size compared to the neat TPG foam due to the higher gas diffusivity and lower melt viscosity of the blend.

**Table 4:** Representative studies that have been reported for PBS foams.

Objectives	Foaming Conditions	Main Results	Ref.
Investigate the foaming ability of PBS grades by single-screw extrusion using CBA.	Single-screw extrusion using an endothermic CBA (sodium bicarbonate) on an industrial extrusion line.	The influence of melt rheology on foam structure was established and cell sizes/density were efficiently adjusted by the melt viscosity. Low-viscosity polymers tend to produce foams with low cell density and higher average cell size, whereas the opposite effects are observed in high-viscosity polymers. Branched polymer structures with strain-hardening effects in extensional flows should be preferred over linear polymers as they promote the higher stabilization of the cell growth. PBS was shown to have high sensitivity to the residence time within the extruder due to interfacial tension between CO_2_ and the molten polymer.	[98]
Study the effect of dicumyl peroxide on the crosslinking neat PBS foaming materials.	PBS, crosslinking agent dicumyl peroxide, and CBA azodicarbonamide were melt mixed in an intensive mixer. Foams were obtained by compression moulding at 120 °C.	Crosslinking degree, viscosity, and storage modulus of PBS increased with increasing content of crosslinking agent. PBS foams with an expansion ratio of 7.03, average cell size of 200 μm, and cell-closed porosity percentage of about 94% were obtained with 6 wt.% of crosslinking, 1 wt.% of CBA, and 160 °C as the foaming temperature.	[45]
Fabricate microcellular PBS foams using chain extender (ethylene-glycidyl methacrylate).	Batch foaming in an autoclave at a saturation pressure of 20 MPa and temperature of 115 °C. Foaming triggered by sudden pressure drop.	Small spherocrystals were formed in modified PBS, which were positive to increase the cell density and decrease the average cell size. With increasing chain extender content, the average cell size and volume expansion ratio decreased, and the cell density increased due to enhanced melt strength and viscosity caused by the chain extension.	[336]
Improvement in the conductivity of PBS/carbon nanotube (CNT) conductive polymer composites by supercritical CO_2_ foaming.	Batch solid-state foaming with supercritical CO_2_ in a high-pressure autoclave. Foaming was triggered by rapid depressurization.	Adding CNTs significantly improved the thermal and electrical conductivity as well as the crystallization, viscoelasticity, and mechanical properties. Foaming increased the electrical conductivity of the nanocomposite (foamed PBS with 5 wt.% CNTs with cell size of 15.6 μm and cell density of 1.03 × 10^7^ cells/cm^3^) by 104% compared to solid PBS/CNT nanocomposite.	[338]
Fabricate a porous PBS/cellulose nanocrystal (CNC) composite scaffold with a bimodal open-pore interconnected structure.	Two-step depressurization in a supercritical CO_2_ foaming process in a high-pressure autoclave.	Bimodal open-porous PBS scaffold with well-defined bimodal open-pore structure composed of small pores (around 11 μm in diameter) and large pores (about 68.9 μm in diameter), with high open porosity (approximately 95%). Scaffolds showed good biocompatibility, hydrophilicity, in vitro degradation rate, and good mechanical compressive properties (compressive strength of 2.8 MPa at 50% strain).	[15]
Fabricate conductive polymer composites based on PBS and CNTs using different processing conditions.	Melt mixing with hot pressing (145 °C, 10 MPa). Solution mixing with hot pressing (100 °C, 60 MPa).	Composites prepared by solution mixing and hot pressing showed improved mechanical properties, electrical conductivity, and thermal conductivity compared to the composites prepared by melt mixing and hot pressing.	[342]

### 4.4. Polyvinyl-Alcohol (PVA)

PVA is a water-soluble synthetic biodegradable and non-toxic polymer consisting of 1,3-diol units or 1,2-diol units, depending on the conditions used in the polymerization. PVA has a semi-crystalline structure containing mostly amorphous phases with only a small portion of crystallinity [343]. It shows excellent biocompatibility and chemical stability, and it is broadly used in medicine, such as in the manufacturing of medicine cachets, biological carriers, in food packaging, coatings, and detergents, among other uses [35]. The properties of PVA are strongly related to its molecular weight, crystallization behaviour, and crystal structure [35]. In addition, PVA is a highly hydrophilic polymer due to the large number of hydroxyl groups in the polymer chain, which makes it moisture-sensitive.

The foaming of PVA is strongly influenced by its high melt strength that results from a high degree of hydrogen bonding. In addition, the melting and degradation temperatures of PVA are very close; therefore, plasticizers are used to disturb the crystalline regions of the polymer and lower its melting temperature, in addition to increasing its fluidity and thermal stability. Following this approach, PVA is usually blended with Poly(ethylene glycol) (PEG), which acts as a plasticizer and nucleating agent. Yin et al. [212] blended PVA with PEG at different concentrations and supercritical CO_2_ as foaming agent, which lowered the melting point and enlarged the processing window of PVA. The plasticizer affected the crystallization and the melt strength of PVA, thus improving the foaming process of PVA. The results showed an increase in the cell size and in the expansion rate of the foam with increasing plasticizer content. Liu et al. [213] also blended PVA with PEG to prepare polymer scaffolds by thermal processing and subsequent bimodal supercritical CO_2_ foaming and obtained a bimodal interconnected open-cell morphology. The larger pores were generated from the nucleation sites in the PVA phase during the rapid depressurization step, while smaller pores were formed in the PEG phase and were incorporated in the larger pores from the PVA phase. Zhao et al. [164] developed a method that used microwave irradiation and water as a blowing agent to trigger foaming and produce PVA foams. After mixing PVA with water, the mixture is allowed to rest for some time so that water can diffuse within the PVA sample. Due to the absorption of microwave power, the temperature rises and both the free energy barrier for bubble nucleation and the surface tension between the gas and liquid phase decrease, triggering cell nucleation and further growth. Nucleating agents can be added to PVA to improve the efficiency of foaming by increasing the number of nucleation sites and decrease the nucleation barrier, although the induced polymer crystallization might hamper the diffusion of a gaseous blowing agent. Li et al. [344] prepared PVA/montmorillonite nanocomposites by melt intercalation and studied their foaming behaviour by melt-extrusion using azodicarbonamide as a CBA. Montmorillonite acted as a nucleating agent and instigated the crystallization of PVA. Improved melt strength and increased complex viscosity were achieved due to the strong interaction between PVA and montmorillonite, which limited the motion of PVA molecules. The results showed a decrease in cell coalescence and the rupture of the composite foams, thinner cell walls, and lower cell size distribution with increasing montmorillonite content. Xiang et al. [345] investigated the effect of several amines (formamide, ethylenediamine, polyamide dendrimer, and polyhedral oligomeric silsesquioxane) in the foaming behaviour of PVA. Results showed that formamide and polyhedral oligomeric silsesquioxane had some plasticization effects on PVA and that formamide and ethylenediamine had a nucleating effect that improved the foaming behaviour of PVA. Song et al. [346] used starch as a pore-forming agent and formaldehyde as a crosslinking agent to produce porous PVA foams reinforced with cellulose nanocrystals. During the process, starch granules swelled at higher temperatures and under acidic conditions, prompting the hydrolysation of starch and filling a space within the PVA matrix. Upon the complete acetalization of PVA and aldehyde, the hydrolysed starch is leached with water, leading to the formation of the pores. The results showed an increase in the foam density and a decrease in the average pore size, as well as improved mechanical performance, with the addition of cellulose nanocrystals. Azimi et al. [347] investigated numerically and experimentally the pore nucleation and growth in PVA during foaming while using supercritical CO_2_ as a blowing agent. They reported an increase in the average of pore density with an increasing depressurization rate during foaming. Moreover, pore growth was controlled by the PVA viscosity and CO_2_ diffusion.

### 4.5. Poly-Hydroxyalkanoates (PHAs)

PHAs are natural aliphatic polyesters produced and gathered within the cytoplasm of the cells by microorganisms via the fermentation of renewable and bio-based feedstocks, which are then extracted [348]. PHAs are sustainable polymers with great potential to replace synthetic petrol-based polymers. PHAs with tailored chemical structures and molecular weights can be processed using conventional technologies such as extrusion, injection moulding, film blowing, film casting, and fibre spinning and have been used in a wide range of applications, such as packaging (foams and bags), automotive, disposable cutlery, office and personal utensils (pens and toothbrush and razor handles), and agriculture mulch films, among others [348]. Among PHAs, poly-3-hydroxybutyrate (PHB) has been the target of special attention given the similarity of its properties to those of Polypropylene. P3HB has a linear chain made of 3-hydroxybutyric acid residues linked by ester bonds [349]. Other poly-hydroxybutyrates include poly 4-hydroxybutyrate (P4HB), polyhydroxyvalerate (PHV), and their copolymers. P3HB has a crystallinity of more than 50%, with a melting temperature of 180 °C and a glass transition temperature of 55 °C [11]. P3HB is quite unstable at temperatures above its melting temperature and even at temperatures slightly below its melting temperature, this polymer can undergo a reduction in its chain length. In addition, the low impact strength, stiffness, and excessive brittleness of P3HB limits the practical applications of this material. To circumvent these situations, P3HB is normally copolymerized with other alkanoates or blended with other polymers [11]. An improvement in PHB was the development of Poly 3-hydroxybutyrate-co-3-hydroxyvalerate (PHBV). PHBV is a semi-crystalline copolymer that is mainly produced from the bacterial fermentation of several bio-based feedstocks, such as plant oils and sugars, and consists in the introduction of the monomer hydroxy valeric acid in the molecular structure of P2HB through copolymerization [350]. PHBV is biologically compatible and presents higher flexibility, ductility, and toughness than P3HB [351]. Moreover, PHBV shows barrier properties to water and air, making it a potential candidate for disposable packages and other uses [352].

PHBV has a narrow processing window (between 145 and 170 °C), which prevents the efficient control of its viscosity through temperature control, and it is processed at temperatures close to its degradation temperature, which increases the possibility of the occurrence of both hydrolysis in the presence of water and thermally induced chain scission [353]. The proximity of melting and degradation temperatures makes the continuous processing of PHBV by extrusion and foaming a challenge. In addition to the vulnerability of PHBV to thermal degradation at temperatures close to its melting point, the use of this polymer in foaming is further hampered by its low melt viscosity and slow crystallization rate. Szegda et al. [354] investigated the extrusion foaming of PHBV using sodium bicarbonate and citric acid as CBAs and calcium carbonate as a nucleation agent. They used a negative gradient temperature profile, to minimize the thermal degradation and improve the melt strength, to achieve the stabilization of the pore morphology and obtained a density reduction near 60%. However, the melt viscosity decreased with higher amounts of the CBA due to increased hydrolytic degradation in connection with water release during the decomposition of the sodium bicarbonate. The difficulties associated with continuous extrusion and foaming of PHBV can be circumvented by the addition of a post-extrusion step, to both cool the foams after exiting from the die and to favour crystallization as a reinforcement mechanism to increase the melt strength and prevent cell coalescence and yield a more uniform pore structure [353]. In addition, CBAs such as azodicarbonamide can be used instead of sodium carbonate to avoid the release of water during the thermal decomposition of the CBA [46]. Xu et al. [355] conducted an investigation on PHBV foaming using supercritical CO_2_ as a PBA. They reported microcellular structures with cell densities ranging from 10^8^ to 1.2 × 10^9^ cells/cm^3^ and average cell sizes ranging from 6 to 22 μm by selecting suitable temperatures (145 to 165 °C) and different CO_2_ saturation pressures (10 to 29 MPa) during foaming. The CO_2_ affected the crystallization behaviour of PHBV foams, as the crystallization of foamed PHBV occurred during cell growth. Moreover, stretching during cell growth enhanced the crystal nucleation rate and the formed crystals further accelerated the crystallization rate. The average cell size increased with increasing temperature, while the cell density and relative density decreased, as the solubility of CO_2_ in the PHBV is lower at higher foaming temperatures. Oprica et al. [356] fabricated PHBV-based foams using expandable microspheres as a blowing agent and bacterial cellulose nanofibers to improve biocompatibility and also as a reinforcing agent. The obtained PHBV foams showed a well-organized porous structure with a porosity of 65% and the presence of both small and large pores. The presence of expandable microspheres and the cellulose nanofibers in the composites showed the opposite effect both in the degradation temperature and in the stiffness of the composite, with the expandable microspheres decreasing the degradation temperature and lowering the stiffness.

**Figure 15:**
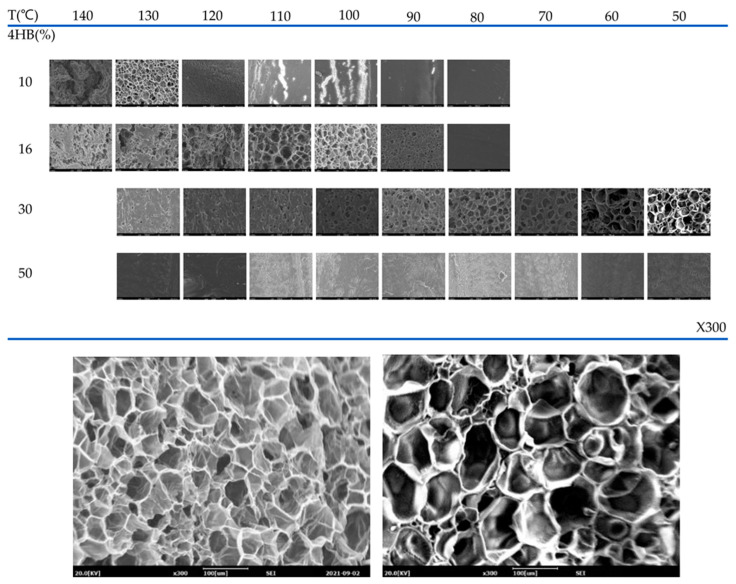
The temperature at which foaming is triggered depends on the 4HB content. In the case of P(3HB-co-4HB) with 10% of P4HB, foaming develops when processed at 130 °C, while when using 16% of P4HB (lower relative crystallinity), foaming develops when processed at 100–110 °C. Using 30% P4HB, the copolymer is non-crystalline, and foaming is triggered at a lower temperature (50–80 °C). When using 5% P4HB, foaming is not achieved in the temperature range studied. Reproduced with permission from Zhang et al. [358].

Other PHA copolymers, other than PHBV, can be made by bacterial synthesis from several monomers, such as hydroxyhexanoate to prepare poly(3-hydroxybutyrate-co-hydroxyhexanoate) (PHBHHx) and 4-hydroxybutyrate to prepare poly(3-hydroxybutyrate-co-4-hydroxybutyrate) (P(3HB-co-4HB)). PHBHHx has a higher amorphous content that could be useful to achieve higher expansion and can be made with higher molecular weight, and thus higher viscosity, which could offer improved melt strength. However, its crystallization time tends to be longer, which could negatively affect the foaming process. To circumvent the higher crystallization time, PHBHHx could be blended with PHBV to take advantage of PHBV, as a nucleating agent, in promoting solidification and avoiding cell coalescence in PHBHHx [357]. The foaming of P(3HB-co-4HB) has been studied by Zhang et al. [358] using supercritical CO_2_. The effect of monomer composition on foaming was investigated and it was reported that an increase in the amount of P4HB decreases the crystallinity of P(3HB-co-4HB) needed for the cell growth, thus requiring a lower temperature for foaming, with less crystallinity and showing improved foamability and more uniform cells (Figure 15).

As already mentioned for other polymers, branched structures can improve the melt strength and, consequently, the foamability of PHA. Ventura et al. [225] used an epoxy-functionalized chain extender to increase the molecular weight, and in this way the melt viscosity and the melt strength, of poly(3-hydroxybutyrate-co-4-hydroxybutyrate) (P3HB4HB); however, the viscosity decreased and coarser cellular structures were obtained, although the addition of the chain extender prevented the polymer degradation. Fillers have also been used in PHA to improve its overall properties and the foamability. Panaitescu et al. [226] used nanocellulose and expandable microspheres containing a blowing agent to obtain PHBV/foams with uniform cells, high energy absorption, and good deformability. The mixing of the polymer, nanocellulose, and expandable microspheres containing low-boiling-point isopentane as a blowing agent was performed in an intensive mixer and the subsequent foaming was carried out by compression moulding. They reported foams with near-perfect pores and a closed-cell structure with a density 2.5–2.7 times lower than that of neat PHBV. Nanocellulose contributed to a more uniform cell size distribution and the stabilization of the cell structure. Besides improving the cell morphology, fillers can be added to PHA to achieve specific properties such as electromagnetic interference (EMI) shielding. In this capacity, graphene nanoplates (GNPs) were added to PHBV and foamed with supercritical CO_2_, yielding a lower volume expansion rate and pore size with increasing GNP content [359]. Table 5 shows some other usages of PHA in foaming processing.

### 4.6. Polybutylene Adipate-Co-Terephthalate (PBAT)

PBAT is a petrol-based biodegradable aliphatic-aromatic co-polyester, synthetized by the polycondensation of 1,4-butanediol with a mixture of adipic and terephthalic acids. The melting temperature of PBAT is 120 °C and the glass transition temperature is about −35 °C. PBAT shows excellent properties, such as high flexibility and toughness, and at a terephthalic acid content of more than 35% mol, it shows good biodegradability. However, the biodegradation rate decreases for terephthalic acid concentrations above 55% [10]. PBAT applications extend across many areas, including bottles, films, and moulded products. However, the expansion in the use of PBAT is hindered by its high production cost and low thermomechanical resistance [362]. The mechanical properties of PBAT are also closely related to the structure and to the molecular weight of the polymer. With increasing terephthalate content, elongation at break decreases, while Young’s modulus increases [363,364]. In addition, elongation at break decreases and tensile strength increases with the increasing molecular weight of the polymer [289]. The mechanical and thermal properties of PBAT can be improved by blending with starch, cellulose, and other biodegradable polymers that show complementary characteristics to those of PBAT to widen its range of applications [11]. For example, PBAT shows a mild stiffness, but blending PBAT with PLA, a material that shows high stiffness and low flexibility, offers a possibility of obtaining a material with better mechanical performance and which is still biodegradable [365].

**Figure 16:**
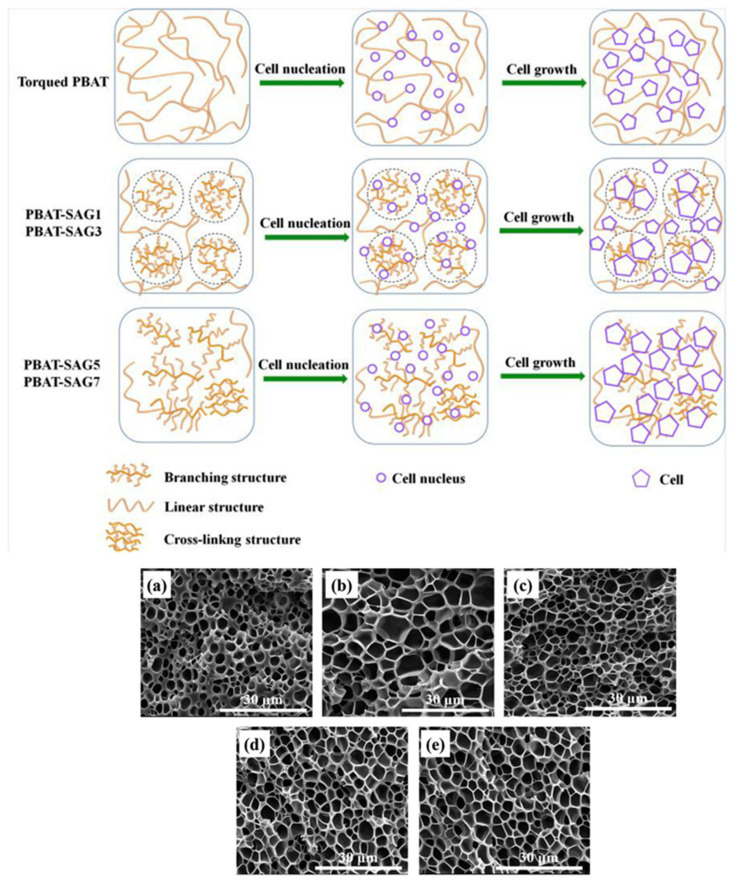
Microcellular morphology of several PBAT foams at the foaming temperature of 80 °C: (**a**) PBAT foam, (**b**) PBAT-SAG1, (**c**) PBAT-SAG3, (**d**) PBAT-SAG5, (**e**) PBAT-SAG7. Adapted with permission from Song et al. [367].

The preparation of PBAT foams presents some difficulties that are related to its low molecular weight, the linear structure of its polymeric chain, its poor melt strength, and its relatively fast crystallization rate. Some strategies can be applied to address these difficulties. Like other polymers, chain extension and branching have also been attempted with PBAT. Song et al. [366] modified PBAT by chain extension using an epoxy-based chain extender to improve the rheological properties, the crystallization behaviour, and the foamability of PBAT. The introduction of the chain extender increased the average molecular weight and improved the viscosity of the polymer, and the crystallization temperature increased from 74.2 to 86.9 °C. Supercritical CO_2_ foaming of the chain-extended PBAT yielded microcellular foams with cell density larger than 10^10^ cells/cm^3^ and average cell size lower than 4 μm. With higher amounts of the chain extender, both the cell density and the volume expansion ratio increased, from 3.4 × 10^10^ to 8.7 × 10^10^ cells/cm^3^ and from 1.5 to 2.0 times, respectively. The same research team studied the effect of chain extension with styrene-acrylonitrile-glycidyl methacrylate terpolymer (SAG) on PBAT foams prepared by solid-phase batch foaming in the presence of supercritical CO_2_ as a blowing agent [367]. Results showed an increase in the branching degree and in the intrinsic viscosity of modified PBAT, as well as an increase in the crystallization temperature and crystallinity, which enabled the production of PBAT foams with complex cellular structures, as observed in Figure 16. The cell walls of PBAT foam were thicker compared to those of PBAT-SAG foams due to the low melt strength of PBAT that was not able to support the cell growth, yielding a small cell size. The introduction of the chain extender (SAG) led to the formation of a complex cellular structure. The average cell size increased with increasing SAG content. However, with SAG contents of 5 and 7 wt.%, the complex cellular structure was not formed, and the cell structure became more homogeneous again due to the saturation of the branching structures.

Besides the use of epoxy-based chain extenders to enhance the melt strength and viscoelasticity of PBAT, Cui et al. [368] added ACNCs to further improve the crystallization behaviour and rheological properties of chain-extended PBAT/ACNC nanocomposites. The introduction of ACNC nanoparticles led to an increase in cell density and a decrease in both the average cell size and the cell size distribution due to the heterogeneous cell nucleation effect of ACNC nanoparticles. An approach for manufacturing PBAT foams with bimodal cellular structure was also reported by Cui et al. [369], which used an Ethylene-glycidyl methacrylate copolymer chain extender and batch supercritical CO_2_ foaming technology. The chain extender improved the melt strength, the viscoelasticity, and the crystallization properties of PBAT. By varying the content of the chain extender and the foaming temperature, it was possible to tune the bimodal cellular structure of the PBAT foams.

The application of electron radiation has been attempted in the foaming of PBAT on the basis that the irradiation may increase viscosity and may positively influence the formation of an appropriate cellular morphology [370]. It was reported that irradiation of PBAT improved its average molecular weight and, therefore, its viscosity. In addition, the foaming of irradiated PBAT yielded foams with a homogeneous cellular morphology and with lower density compared to the foaming of non-irradiated PBAT. Cai et al. [371] reported one of very few studies on the preparation of PBAT bead foams via the supercritical fluid foaming technology using CO_2_ as the blowing agent. The cell density of PBAT bead foams ranged between 5.0 × 10^7^ and 3.0 × 10^8^ cells/cm^3^ depending on temperature and pressure conditions. In addition, the incorporation of PLA improved the mechanical performance of the bead foams. Using optimal foaming conditions, the cell density of the PBAT/PLA bead foam was 4.08 × 10^8^ cells/cm^3^ and the expansion ratio reached 13.44. Wang et al. [223] reported the microcellular foaming of PBAT with mixed blowing agents (N_2_ and CO_2_) to address foam-shrinkage issues related to PBAT microcellular foams. They reported PBAT foams with a high expansion ratio (almost 15-fold) and with limited shrinkage (less than 6%). The use of N_2_ and CO_2_ as co-blowing agents effectively stabilized the foam morphology and limited the shrinkage of cell walls.

Nanofillers, such as carbon nanotubes (CNTs) or graphene nanosheets [372], peach palm tree fibre [373], lignin [374], basalt fibre [375], and nanoclay [376], have been reported as reinforcements of PBAT foams. Additives are commonly employed to improve the mechanical properties, EMI shielding effect, barrier properties, and thermal stability, among others, of polymers and, in the case of polymer foams, to induce foaming by acting as nucleating agents. The use of fully degradable fillers, such as natural lignocellulosic fibres, are of special interest as they combine the environmental benefit of biodegradability, which is important when using biodegradable polymers, with lightweight, low cost, improved mechanical performance [373,377], and antimicrobial properties [378]. The use of peach palm tree fibre in PBAT composites, compared to neat PBAT foams, yielded foams with a more heterogeneous pore size distribution and a reduced average pore size, with the pores being formed at the interface between the matrix and the fibre [373]. Hong and Hwang [374] fabricated a fully biodegradable foam of a PBAT composite by the addition of lignin up to 50 wt. %, towards the development of a low cost, biodegradable, and sustainable foam to be used in packaging applications. Foaming was achieved by using azodicarbonamide as the CBA and an epoxy-based chain extender, combined with crosslinking agent dicumyl peroxide, which was used to improve the foaming properties of PBAT. They reported that the cell structures of the lignin-reinforced PBAT composites collapsed when the lignin content, without the addition of the chain extender, exceeded 20 wt.%, but the use of the chain extender enabled them to obtain a foam with good quality with a lignin content as high as 30 wt.%. The effects of CNT and graphene nanoplatelet (GNP) content on the crystallization, melting behaviour, conductivity, and foaming behaviour under supercritical CO_2_ of PBAT-based nanocomposites were studied by Wei et al. [372]. The addition of CNTs and GNPs improved the rheological properties and cellular structures of PBAT. The fillers improved the melt viscoelasticity of PBAT and with increasing amounts of filler, a bimodal pore structure was gradually formed in the PBAT composite foams while at the same time the average pore size decreased. The larger pores in the PBAT composite foams were possibly triggered by the CNTs while the GNPs induced the formation of the smaller pores.

### 4.7. Starch

**Figure 17:**
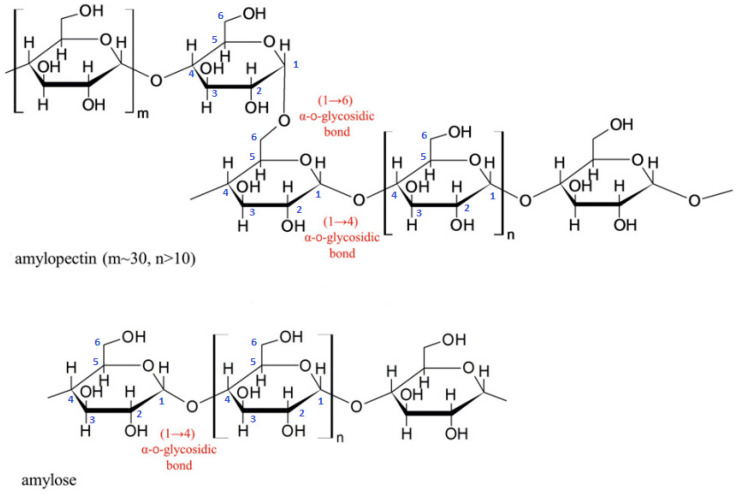
Molecular structures of amylopectin and amylose. Adapted with permission from Wiercigroch et al. [388].

Starch is a polymeric carbohydrate composed of numerous glucose units linked by glycosidic bonds that is used by plants to store carbohydrates. It is composed of a mixture of amylose, a linear and helical polysaccharide, and amylopectin, a highly branched and high-molecular-weight polysaccharide (Figure 17). Amylose is made of α-1–4 linked glucopyranosyl units, with a molecular weight in the order of 104 to 105 and a degree of polymerization between 250 and 1000 of D-glucose units [379]. Amylopectin is made of (1–4)-linked α-D-glucopyranosyl units in chains linked by (1–6) linkages with a molecular weight in the order of 106 to 108 and has a degree of polymerization of about 5000–50,000 D-glucose units [153]. Depending on the plant, such as wheat, corn, potato, and rice, starch generally contains 18 to 28% amylose and 72 to 82% amylopectin by weight [379,380]. The relative composition between amylose and amylopectin has a major influence on the overall properties of starch [381,382]. Starch-based films with higher rates of amylose tend to show both a higher Young’s modulus and tensile strength and lower elongation at break compared to those with low amylose rates [383]. Starch has some drawbacks that prevent its direct usage as a material capable of replacing basic petrol-based polymers, such as its highly hydrophilic nature and its overall poor mechanical properties, which limits its usage in foaming or in other possible applications such as in packaging [36]. Alternatively, starch is frequently used as plasticized starch or thermoplastic starch (TPS). Starch is plasticized by the combined effect of shear and heat with the aid of plasticizers such as water or glycerol, the most used plasticizers, or other molecules capable of forming hydrogen bonds with the hydroxyl groups of starch, such as sorbitol, sucrose, glucose, and fructose [380,384,385]. The plasticizer improves chain mobility by decreasing the inter-molecular bonds between polymer chains [386]. The TPS can then be melted at elevated temperatures and flow like any other thermoplastic to make films, moulded articles, and foams by conventional techniques, such as extrusion or injection moulding. However, TPS plasticized with water has poor dimensional stability and exhibits poor mechanical performance, in addition to being hygroscopic. With moisture, the properties of TPS tend to deteriorate further, while with a loss of water, TPS becomes brittle. These problems can be overcome by mixing TPS with other polymers or fillers using high-temperature extrusion [36,387].

**Figure 18:**
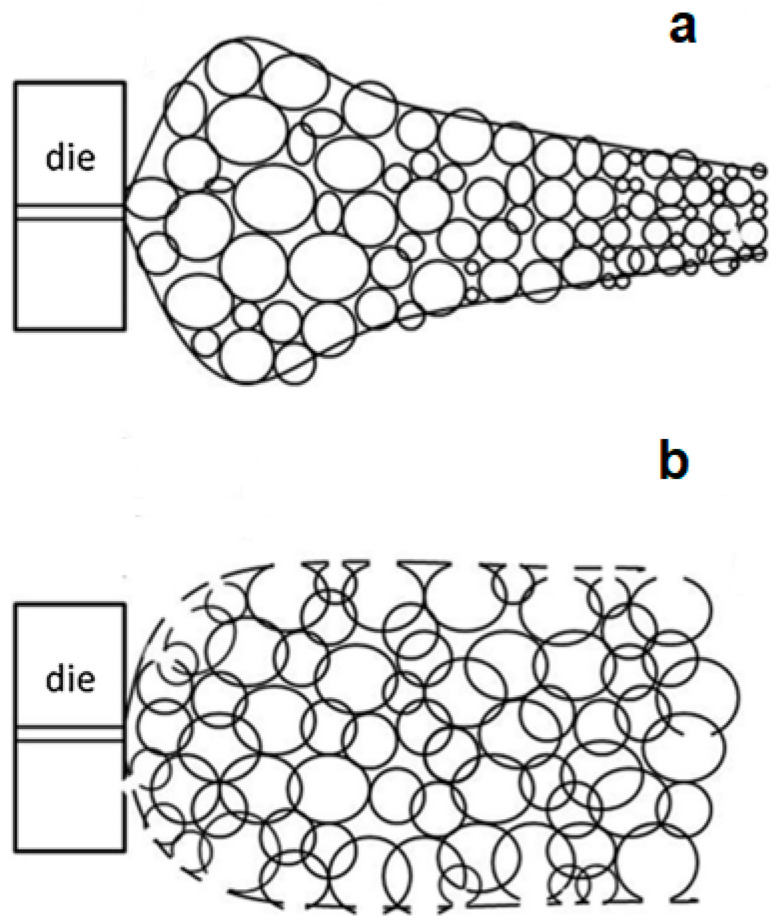
Diagram representing the exit of the starch-based material from the die using steam: (**a**) sample containing higher level of moisture that yields foams with closed-cell structure and shrinking; (**b**) sample containing low levels of moisture that yields foams with open-cell structure and solidified bubbles. Reproduced with permission from Meng et al. [384].

A commonly used technique to produce starch-based foams is the baking technique [153]. In this system, a batter of starch and water is baked in a closed and heated mould for a few minutes. During the process, the starch granules gelatinize, resulting in the formation of a thick paste as the retained water evaporates rapidly, which leads to a large expansion of the paste. Residual water evaporates further as the starch thick paste fills up the mould cavity and eventually the dry paste takes the shape of the mould [7]. Starch-based foam properties can be tailored by paste composition (starch source and ratio with water), baking temperature (in the range of 180–250 °C), and baking time (usually between 125 and 300 s). Starch-based foams can also be processed by extrusion. Extrusion has some advantages compared to baking as it is a continuous process, which can lower the production costs, besides producing a homogeneous mixing with lower residence times [389]. The extrusion of a mixture of starch and water at temperatures above 100 °C originates pores due to water evaporation and steam expansion as the material exits the die. The foam eventually takes the shape given by the profile of the die. Foam properties can be tailored by starch source, starch–water ratio, and extrusion processing parameters, such as the temperature profile, screw speed, feed ratio, and cooling rate [7]. Aguilar et al. [390] studied the influence of the ratio between cassava starch and avocado seed, which contains 41% of starch content, in the properties of starch trays produced by thermocompression. The inclusion of avocado seed yielded thicker and denser trays but with lower porosity compared to trays based only on cassava starch. In addition, the composite trays showed higher stiffness, higher hydrophobicity, and more water resistance due to the presence of proteins, lipids, and fibres and due to the higher amylose content in the avocado seed. Meng et al. [384] reported a critical point in the amount of water (between 16 and 18%) at which the expansion ratio changes significantly and as the pore morphology changes from open to closed. With lower amounts of water, the melt temperature of starch increased and melt strength decreased, which results in foams with a stable and open-cell morphology. On the contrary, higher amounts of water and lower melting temperature yielded increased melt strength, a closed-cell morphology, and foam shrinkage. This effect can be observed in the diagram of Figure 18.

Several studies have focused on various strategies to overcome the limitations of starch foams, including their overall poor mechanical performance and high-water absorption. These strategies include modifications of starch by oxidation with hydrogen peroxide [391], by acetylation [392], by esterification [392], silylation [154,392], by citric acid [393,394], and by the use of alkyl ketene dimer (AKD) as a sizing agent [395]. Rostamabadi et al. [396] published an interesting review paper that addresses the strategies for modifying starch through its combination with other molecules.

Bergel et al. [397] developed a solution to circumvent the problem of the high water absorption of starch by coating the TPS foam with PLA, as a hydrophobic material, to prevent the contact of TPS with water. The starch foams were made by mixing potato starch, water, and glycerol as a plasticizer at 70 °C until gelatinization and homogenization, and subsequent compression moulding took place at 180 °C with 2.5 tons of pressure for 240 s. The PLA coating was produced by immersing the starch foam in a PLA solution. Results of the PLA-coated starch foams showed a reduction of 225% in water absorption when compared to TPS foams. Reis et al. [398] coated a PLA/starch foam with a beeswax emulsion to produce biodegradable trays with reduced water vapor permeability. Results have shown a sharp decrease in water vapor permeability with higher beeswax content. In another reported study, starch was modified with silanes (3-chloropropyl trimethoxysilane and methyltrimethoxysilane) to exchange starch hydroxyl groups for the less polar silane groups and make starch less water sensitive [154]. Results from water absorption and mechanical tests have shown that these silane-modified starch foams absorb less water and become more resistant. Iriani et al. [395] modified the surface of cassava starch-based foam, using alkyl ketene dimer (AKD) as a sizing agent to improve the hydrophobic characteristics of starch foam, and reported a reduction in the water absorption of AKD-coated starch by an average of 83.26% compared to the foam without the AKD coating.

Other works have focused on modifying starch through the addition of fillers to improve the overall properties of starch foams. The used fillers include fish scale powder [399], coconut residue fibre [400], cellulose fibres [394], cellulose nanofibers [401], cellulose microfibres [402], sesame cake [403], sugarcane bagasse fibres [404,405], grape stalks [406,407], peanut skin [408], barley straw fibres [163], sisal fibres [409], silica or nano-silica particles [410,411], aluminium hydroxide [412], calcified green macroalga [413], egg shell and shrimp shell [414], natural oat fibres [415], sunflower oil press cake [416], water hyacinth fibres [417], Khlum fibres [418], and cotton fibres [419]. Kaewtatip et al. [414] reported starch/egg shell composite foams prepared by a baking process with lower density (0.2056 g/cm^3^), a narrower pore size distribution, and higher impact strength compared to neat starch foams and attributed the improvement of the foam properties to the steady growth of steam bubbles due to the nucleating effect of the egg shell which helped to prevent the bubbles from collapsing during the baking process. Versino et al. [416] investigated the use of urea as a plasticizer and crosslinking agent and of sunflower oil press cake as a reinforcing agent in starch foams obtained by thermocompression. They also investigated the contribution of urea to foaming, as urea decomposes at 150 °C by releasing NH_3_ and CO_2_, which can act as gaseous blowing agents and contribute to the foaming process. The inclusion of the sunflower oil press cake changed the structure of the starch foams, yielding denser and harder materials with improved water uptake capacity. Moreover, urea addition yielded foams that were more flexible to flexural deformation. Nugroho et al. [417] reported that the addition of 10% water hyacinth fibre enhanced the physical and mechanical properties of starch foams; however, it increased the amount of filler and showed some degradation of the foam properties, such as a lower compression strength, increased water absorption, and foams with a darker appearance.

The moisture barrier of starch/cellulose composite foams can be improved by blocking some of the hydroxyl groups of starch and cellulose through crosslinking using citric acid as crosslinking agent. These starch/cellulose composite foams crosslinked by citric acid and fabricated by the compression moulding technique have shown increased thermal stability, tensile strength, and flexural strength and decreased hydrophobicity and water absorption capacity [394].

Blending starch with other polymers is another alternative to improve the moisture resistance of starch-based foams. PVA is a good alternative to be used in blending with starch due to its biodegradability, which makes the starch/PVA still biodegradable, and due to its polar nature and its good compatibility with starch, which results in a single-phase mixture [420]. Kahvand et al. [421] studied the effects of PVA and glycerol/water plasticizer contents on the cellular morphology of starch foams prepared by melt extrusion. Blending starch with 20% PVA enabled them to obtain foams with lower density (decreased about 70%), higher cell density (increased up to 60-fold), lower water absorption (decreased about 60%), and a more uniform cell structure compared to starch foams. Table 6 shows some other representative studies that have been reported for starch foam materials.

### 4.8. Poly (Propylene Carbonate) (PPC)

PPC is a biodegradable amorphous aliphatic thermoplastic copolymer of carbon dioxide and propylene oxide that shows some promising properties such as good oxygen barrier properties and ease of processability. In this context, it is being increasingly used in films for agricultural mulching, packaging [424,425], and is considered in electromagnetic interference (EMI) shielding and sensor systems [426]. However, the widespread use of PPC is limited by performance limitations, such a low glass transition temperature (35 °C), low mechanical strength, poor dimensional stability, and low thermal degradation temperature. To overcome some of these problems, various approaches have been tried, including increasing the molecular weight of the polymer or mixing it with other polymers [427]. Regarding foaming, PPC shows poor intrinsic melt strength, which, combined with the low glass transition temperature, is a major hindrance to prepare PPC polymeric foams with good quality [428]. However, PPC is a polymer with high affinity towards CO_2_, capable of absorbing 23.1 wt.% of CO_2_ [215].

Manavitehrani et al. [214] studied the processing parameters of gas foaming to optimize the porosity, pore size, and pore interconnectivity of PPC scaffolds for tissue engineering applications. They found that by using slower depressurization rates, both the average pore size and the porosity increased, given that with a slow depressurization rate, the cells had more time to grow into large pores, while a quick depressurization rate generated higher supersaturation and a nucleation rate that yielded small pores and a lower overall porosity. In addition, they reported that the pore size increased significantly with increasing temperature while keeping the pressure constant due to decreased polymer viscosity, which facilitated CO_2_ solubilization within the polymer matrix and led to larger pores.

Most studies of the foaming of PPC reported in the literature use PPC blended with other polymers. Kuang et al. [428] used PPC blended with PBS and polytetrafluoroethylene (PTFE) to significantly improve the melt strength and foaming ability of PPC without compromising their excellent biodegradability. During extrusion, the synergistic effect between the PBS domains and the PTFE nano-fibrils network improved the foaming behaviour of PPC and yielded higher cell densities (by two orders of magnitude) and a higher compressive modulus (30-fold). Liu et al. [215] blended PPC with PBAT using an epoxy-based chain extender as a compatibilizer in an effort to improve the compatibility between PPC and PBAT. The blend was saturated with CO_2_ at 90 °C and high pressure after which foaming was triggered by the release of pressure, yielding uniform and resilient biodegradable foams with a density of 0.083 g/cm^3^ and average cell size of 15 µm.

Many efforts have been carried out to circumvent the poor mechanical and thermal properties of PPC. A common strategy, also used in other polymers, resorts to the use of reinforcements or fillers, such as graphene oxide [429], CB [430], carbon nanostructures [426], and calcium carbonate (CaCO_3_) [431,432], among others. Among these fillers, nano-CaCO_3_ shows special interest in foaming due to its ability to increase the melt viscosity of the matrix and at the same time act as a nucleating agent. Yu et al. [432] prepared and foamed PPC/nano-CaCO_3_ composites using supercritical CO_2_ as a blowing agent. They reported an increase in the glass transition temperature with the addition of nano-CaCO_3_. In addition, the homogeneous dispersion of the fillers, achieved with 3 wt.%, yielded the finest cell structure with a narrow cell size distribution. Higher amounts of nano-CaCO_3_ yielded larger pores due to the aggregation of the fillers. Cui et al. [430] fabricated conductive chlorinated PPC/CB foams with a well-defined closed-cell structure by a two-step method combining melt blending and subsequent chemical foaming using azodicarbonamide as the foaming agent. They reported a decrease in the electrical percolation threshold from 2.48 vol% (solid composites) to 0.14 vol% after the foaming process. Moreover, the foams showed lower resistance with increasing temperature due to the thinning of cell walls induced by the gas expansion and the subsequent lower distance between adjacent CB particles.

Yang et al. [429] added graphene oxide into PPC to improve the thermal stability and the mechanical strength of PPC and reported a 10 °C increase in its glass transition temperature after incorporation of the filler. The obtained PPC/graphene oxide composites were subsequently foamed using supercritical CO_2_ with the aim of evaluating the effect of the saturation pressure, saturation temperature, and saturation time in the preparation of scaffolds for tissue engineering applications. With an increase in temperature, an increase in pore size was observed due to lower viscosity of the polymer matrix that facilitated the foaming. An increase in the saturation pressure also yielded an increase in pore size. At last, with increasing saturation time, larger pore size and lower pore density were obtained. It is believed that longer saturation time resulted in more sorption of CO_2_. The increase in both saturation pressure and saturation time led to the increased adsorption of CO_2_, which decreased the viscosity of the nanocomposite and increased the period of pore growth, resulting in larger pores.

### 4.9. Biodegradable Polymer Blends

Most biodegradable or bio-based polymers have some interesting mechanical properties to add to the sustainability factor and can be considered as adequate replacements for petroleum-based polymers in some applications. However, these biodegradable polymers have some substantial setbacks such as low melt strength, slow crystallization rate, poor processability, low service temperature, low toughness, and high brittleness, which limit their field of application. To surpass these limitations, bio-based or biodegradable polymers can be reinforced with fillers with the intention of improving certain properties. Alternatively, they can be blended with other polymers to combine certain properties of the individual components of the blend to counteract certain limitations. For instance, PLA is one of the most used biodegradable polymers because of its good overall mechanical properties; however, its poor melt strength and low crystallization rate pose great processing challenges regarding foaming. To overcome these limitations and to improve its properties, PLA has been blended with various biodegradable polymers such as PCL, PBAT, PBS, and Novatein.

PCL has a high elongation at break and toughness and excellent crystallization properties, although it has low tensile strength, so blending PCL with PLA can decrease the brittleness and improve the ductility and toughness of PLA and at the same time improve the tensile strength of PCL. Considering their complementary properties, blending PLA with PCL becomes a good alternative to improve the properties of PLA without compromising its biodegradability [133,250,433,434]. Zhao et al. [435] blended PCL with PLA to manufacture a vascular scaffold foam with open-cell morphology by one step supercritical CO_2_ batch foaming. Differential scanning calorimetry measurements showed that PCL and PLA were immiscible. The open-cell morphology of the scaffold was achieved at 40 °C and a sodium hydroxide solution was subsequently used to hydrolyse off the PLA phase from the scaffold surface and improve the porosity and hydrophily in order to improve the mechanical properties and promote the cell culture.

The relative difficulty in preparing PLA foams with open-cell morphology via batch foaming is well known and PCL can be added to PLA to promote the formation of foams with open-cell morphology. In this context, Wang et al. [436] blended PLA and PCL to fabricate open porous PCL/PLA scaffolds for tissue engineering applications. They studied the relationship between, on the one hand, the blend ratio and foaming processing parameters, including the temperature, pressure, and CO_2_ saturation time, and on the other hand, both the foam morphology and the mechanical performance. They reported increased cell size with increasing temperature and time and increased tensile strength and elastic modulus with the increase in the average cell size when higher PCL content was used. When higher PLA content was used, the cell size also increased with increasing temperature and time, but the tensile strength and stiffness decreased with the increase in the average cell size. The nucleation took place at the phase interface between the PCL matrix (soft phase with low melt strength) and PLA (disperse phase with higher melt strength) due to the low interfacial energy. Further cell growth and collision took place, in which the cell walls became thinner and eventually broke, and an open structure was formed.

Sun et al. [434] fabricated high-strength biodegradable and highly interconnected porous polymeric scaffolds of PCL/PLA blends by twin-screw extrusion followed by solid-state supercritical CO_2_ batch foaming, which was controlled by foaming temperature, pressure, and time. They reported a substantial increase in the average cell size and a gradual decrease in the cell size uniformity with increasing temperature, while the opposite occurred with the increase in the foaming pressure. In addition, high porosity and an open-cell content greater than 90% was also obtained. Lv et al. [437] fabricated PCL/PLA (70 wt.% of PCL and 30 wt.% of PLA) foam blends containing fibrils with open-cell morphology and high porosity via a one-step batch foaming process. The interconnected cells with flexible PCL fibrils were generated due to the high tensile stress experienced during cell expansion.

The foaming of thermoplastic blends can be improved using the in situ fibrillation technique [438]. In this technique, in the first stage, a blend of polymers is prepared by extrusion. One of the polymers acts as a matrix while the other, with a melting temperature 40 °C higher than the melting temperature of the polymer used as a matrix, acts as a dispersed phase. The processing temperature must be higher than the melting temperature of both polymers and the melt that exits from the die is either hot-stretched or cold-pulled by a rotating roller. During pulling, the dispersed phase undergoes from spherical to fibrillar domains due to plastic deformation. Finally, fibrillar composites are moulded using a processing temperature that is between the melting temperatures of the two polymers to preserve the nano- or microfibrils of the dispersed phase. Blends with fibrils show improved crystallization kinetics and improved foaming ability. Using this technique, microfibrillated PCL/PLA composites were easily created by melt-blending PLA and PCL using a twin-screw extruder, followed by hot-stretching [438]. The samples before stretching showed spherical PLA domains, which transformed into fibrillar structures after stretching. During batch foaming, the fibrillated samples induced foams with an improved open-cell morphology compared to foams from neat PCL and non-stretched PCL/PLA samples.

Poly(L-lactide-co-ε-caprolactone) (PLCL) is a copolymer from PLA and PCL. PLCL and PCL are completely compatible without phase separation when less than 30 wt.% of PLCL is used. PLCL enables the enhancement of the melt strength of PCL, which allows for an improvement in cell nucleation and growth during supercritical CO_2_ foaming [439]. Using PCL/PLCL blends with a PLCL content of 30 wt.%, foams with 77% open-cell content, pore size of 24.9 μm, and cell density of 1.23 × 10^9^ cells/cm^3^ can be obtained [439]. PLCL can also be used as a compatibilizer between PLLA and PCL phases and chain entanglements that results in an improvement in the stretch ability of the polymer blend [440].

As mentioned before, PBS is a biodegradable aliphatic polyester with properties like those of polypropylene, namely high flexibility, high impact strength, good thermal and chemical resistance, and they are easy to process. These properties of PBS can complement the properties of PLA, by blending PBS with PLA in order to improve the mechanical properties of PLA foams [441]. Several studies report immiscibility between PBS and PLA, although good compatibility and excellent mechanical properties can be attained by blending PLA with less than 20 wt% of PBS [231]. Blending PLA with PBS is a common strategy to produce high-expansion PLA/PBS open-cell foams through the supercritical CO_2_ foaming process. The blending of immiscible polymers enables the dispersion of a soft phase in a hard matrix or the dispersion of a hard phase in a soft matrix, thus providing a heterogeneous melt structure that facilitates cell ruptures. Using this approach, Li et al. [442] prepared highly interconnected PLA/PBS foams with an open-cell structure using supercritical CO_2_ foaming, by using PLA as the matrix and PBS as the cell opener. They reported PLA/PBS foams with an expansion ratio of 43.6 and a porosity of 97.7%. A similar strategy was followed by Sun et al. [443], who reported PLA/PBS foams with an open-cell structure and cell growth at the interface between PBS and PLA phases, and Yu et al. [444], who reported an immiscible PBS phase dispersed as large domains or tiny spheres within the PLA matrix that yielded open-cell morphology with high rates of open cells (97%). Vorawongsagul et al. [10] added cellulose fibres to PLA/PBS blends to prepare PLA/PBS/cellulose fibre composite foams by extrusion foaming using sodium bicarbonate as the blowing agent. The addition of cellulose fibres to the PLA/PBS blend decreased the viscosity of the blend and yielded a higher number of closed cells and a more uniform cell distribution. Shi et al. [137] fabricated microcellular PLA/PBS foams by the batch foaming process using supercritical CO_2_. PLA and PBS were shown to be immiscible and the PBS phase formed separated domains within the PLA matrix. PBS decreased the gas solubility of CO_2_, which promoted a larger average cell size and smaller cell density in addition to a more uniform size distribution and improved the crystallization behaviour of PLA. Cells were nucleated around the interface between PLA and PBS, resulting in an open-cell morphology compared to the closed-cell morphology of neat PLA foam. Tian et al. [445] prepared biodegradable PLA/PBS/multi-walled carbon nanotube (MWCNT) nanocomposites with segregated structures by melt blending. They reported that the MWCNTs were mainly dispersed in the PBS phase, which resulted in an ultralow percolation value of 0.071 vol%. With a MWCNT content of 0.499 vol%, and the nanocomposites with segregated structures exhibited an electrical conductivity of 7.15 × 10^−3^ S/m, a value that is six orders of magnitude higher than that found in nanocomposites with normal structures.

The compatibility between PLA and PBS can be improved by compounding the PLA/PBS blend in the presence of dicumyl peroxide. Dicumyl peroxide can be used as an in situ compatibilizer and chain extender for biopolymers through the formation of crosslinked or branched structures between both PLA and PBS polymers, creating improved interfacial adhesion between the two polymers. Campuzano et al. [446] prepared PLA/PBS blends using decumyl peroxide as a compatibilizer and chain extender in an effort to enhance the melt crystallization rate, the viscosity, and the melt strength of the blend. The foams produced by injection moulding using azodicarbonamide as the CBA showed a closed-cell structure, narrow cell size distribution, higher cell density (1.8 × 10^5^ cells/cm^3^), and foam density of 0.85 g/cm^3^. Chen et al. [447] used poly(ethylene glycol) (PEG) to improve the compatibility between PLA and PBS in order to fabricate PLA-based scaffolds with high porosity through supercritical CO_2_ foaming. The PLA/PBS/PEG blend (composition of 90/10/10) foamed under 100 °C showed a volume expansion ratio of 13.98 and open cell rate of 95.9%.

**Figure 19:**
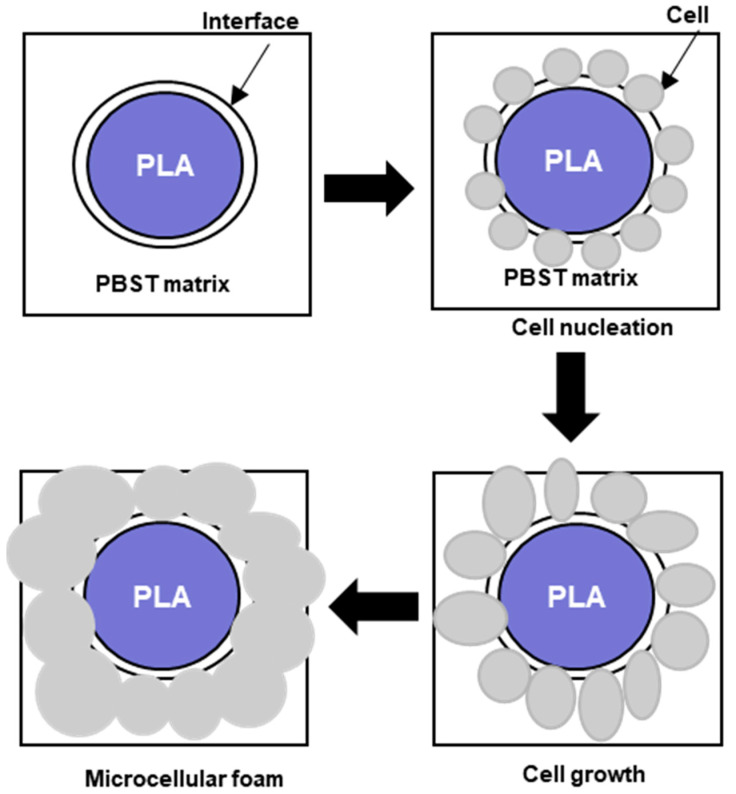
Diagram showing the cell morphology evolution of the PBST/PLA foams. Cell nucleation took place at the interface between the PLA dispersed phase and the PBST matrix. As the foaming temperature was close to the melting point of PBST, the interfacial tension of the PBST phase was much lower than that of the PLA phase and so the nucleated cells tended to grow toward the PBST matrix, creating a flower-like cell structure in which the cells grew around the PLA phase. Adapted with permission from Chen et al. [448].

PLA was also blended with other PBS copolymers, such as poly (butylene succinate-co-adipate) (PBSA) [8,127] and poly(butylene succinate-butylene terephthalate) (PBST) [448] in an effort to enhance the crystallization rate and the thermal properties of PLA. Pradeep et al. [127] studied the foaming of blends of PLA/PBSA compatibilized by coupling agent triphenyl phosphite via injection moulding using supercritical N_2_ as the blowing agent. The compatibilized foamed blends showed an improvement in crystallinity compared to their unfoamed blend counterparts. In addition, compatibilized foamed blends showed higher storage moduli compared to the non-compatibilized foams. Kim et al. [21] investigated the effect of blend morphology on the cell structure of PLA/PBSA foams using several blend ratios that were fabricated by a core-back foam injection moulding process. They reported a millefeuille-like cellular structure for PLA/PBSA (50/50) blends, which was created by synergistic effects between foaming conditions, blend morphology, and viscosity differences between PLA and PBSA. Chen et al. [448] fabricated PBST/PLA microcellular foams by using supercritical CO_2_ as the blowing agent. PLA improved the rheological properties and increased the crystallization temperature of PBST, while at the same time it controlled the solubility and diffusion of CO_2_ in the blends and served as a heterogeneous nucleation centre during foaming, enabling the formation of foams with open-cell morphology. The PLA dispersed droplets acted as the heterogeneous nucleation sites and the cells first nucleated and then grew around them, as shown in Figure 19.

To overcome the brittleness and low toughness of PLA, PBAT can also be selected as a blending option due to its flexibility and toughness. PLA and PBAT generate immiscible blends without the use of any compatibilization agent. Epoxy chain extenders, besides being primarily used as a chain extender of the linear structure of the polymers, can also be used as a compatibilizer to promote the compatibility between PLA and PBAT and subsequently improve the foamability of the polymer blend [449].

PBAT significantly affects the overall mechanical performance and the crystallization behaviour of PLA, as the toughness and ductility of the blend, as well as the crystallization rate of PLA, increase with the addition of PBAT although the modulus and the tensile strength show a decrease [231,450]. Moreover, the addition of PBAT has been shown to improve the rheological behaviour and both the crystallization and the crystal morphology of PLA, which affect the foaming behaviour by preventing the escape of foaming gases, producing changes in the cellular morphology of the PLA foams [451]. The effect of the addition of a second phase of PBAT on PLA foaming has a great influence on the cell morphology, with PLA/PBAT foams showing a more uniform cell distribution with an open-cell structure due to the interface between PLA and the soft immiscible PBAT phase as a separated domain [452]. Zhang et al. [105] prepared PLA/PBAT blend foams using a single-screw extruder and azodicarbonamide as the CBA and reported the improved melt strength, crystallization, and viscosity of the blend due to PBAT. The PLA/PBAT foams showed a more homogenous distribution of the cells with a larger average cell size and more uniform shapes compared to neat PLA foams. Pilla et al. [112] studied the effect of compatibilization in PLA/PBAT blends foamed by the microcellular extrusion process using CO_2_ as a blowing agent and showed that the improved compatibilization between PLA and PBAT generated an increase in the cell density and a decrease in both the average cell size and volume expansion.

Polymer blending was also used as a technique to improve some of the properties of PHVB that are important in foaming, such as low melt viscosity and high crystallinity. Examples include blending PHBV with PCL [352,453], which is a semi-crystalline polymer with high miscibility in CO_2_, with PBAT [376], and with PLA [128,251]. Jenkins et al. [453] showed that PHBV and PCL are immiscible when blended using mechanical means, but the same blends were miscible when produced using supercritical methods. Oluwabunmi et al. [352] blended PHBV with PCL to foam PHBV by a batch foaming process using subcritical CO_2_. A two-stage depressurization was used to increase the time for cell growth and assist the cell nucleation and growth in this way. Foams obtained from neat PCL showed the highest expansion ratio and porosity, and as PHBV was added, the porosity gradually decreased. Zhao et al. [251] blended PHBV with PLA to improve the microcellular foam morphology. The blends were foamed by microcellular injection moulding using supercritical N_2_ as a blowing agent. They found that the PLA/PHBV blends were only miscible when using low PHBV contents, as with a PHBV content higher than 30%, the PLA/PHBV blends were immiscible. In addition, the increase in PHBV content decreased the glass transition temperature in the PLA/PHBV blends, increased the cell density, and decreased the average cell size of the microcellular foams. Brütting et al. [454] also reported that PHBV and PLA are immiscible and that the increase in PHBV content in the blend yields foams with slightly higher foam density and lower cell size due to an increased cell nucleation rate.

Novatein^®^ Thermoplastic Protein (NTP) is a semicrystalline patented formulation of bloodmeal with chemical additives that can be extruded and injection-moulded like any other thermoplastic. However, NTP has poor mechanical properties due to its hydrophilic nature and its tendency to lose plasticizer during use [455]. In addition, neat NTP cannot yield a cellular structure. To overcome these problems, NTP has been blended with other biopolymers such as PLA [455,456]. To improve compatibility between NTP and PLA, the compatibilizer itaconic anhydride was grafted onto PLA using dicumyl peroxide as the radical initiator. Walallavita et al. [456] used this approach to produce PLA/NTP blend foams by a batch foaming method. The obtained PLA/NTP blend (50/50 wt.%) foams showed a cell density of 8.44 × 10^21^ cells/cm^3^ and cell sizes of 3.36 μm, which are a smaller cell size and a higher cell density than those obtained for neat PLA.

PBAT foams normally face shrinkage issues, related to the collapsing and merging of cells, when supercritical CO_2_ is used as the blowing agent. Blending PBAT with other polymers, such as PBS [457,458], PVA [459], PLA, and PPC [460], can be an approach to avoid these issues. Hu et al. [457] studied the foaming of PBAT/PBS blends using supercritical CO_2_ in an effort to provide an anti-shrinkage strategy in order to produce PBAT foams. They reported an improvement in the mechanical performance and in the crystallization behaviour of the blend, although accompanied by a deterioration of melt strength after the inclusion of PBS. In addition, during foaming, PBS improved the cell nucleation and decreased the cell wall thickness, enabling the creation of foams with an open-cell morphology. Li et al. [458] also blended PBS with PBAT to improve the foamability of PBAT by supercritical CO_2_. The regularity of the molecular chain of PBS improved the crystallization behaviour of the blend and ensured higher strength to the foams, while the micro-phase separation structure in the PBAT/PBS blend improved the cell growth and yielded a higher expansion ratio. Tian et al. [460] prepared foams of PBAT/PPC blends by extrusion foaming using Azodicarbonamide as the blowing agent. PBAT was previously modified through the bis(tert-butyl dioxy isopropyl) benzene chain extender. They reported that an amount of 30 wt.% of PPC in the blend yielded foams with a cell density of 2.35 × 10^5^ cells/cm^3^. However, with higher amounts of PPC, the cells collapsed and the cell density of the foams decreased. Instead, PBAT can be used to improve the foam properties of PBS foams. Studies on PBS/PBAT blend foaming have revealed significant improvements in the toughness and flexibility of PBS/PBAT blend foams after adding PBAT to PBS, although the addition of PBAT enhanced the viscosity of the PBS/PBAT blend very slightly and produced only small changes in the average cell size of PBS/PBAT foams compared to PBS foams [461].

PPC presents some drawbacks when it comes to foaming due to poor melt strength. Blending with other biodegradable polymers is a possibility to improve the foaming ability of PPC without sacrificing its excellent biodegradability. Using this approach, PPC was blended with PBS and PTFE particles. The high shear stress during the extrusion process enabled the fibrillation of the PTFE particles into networks within the PPC polymer matrix, and the synergistic effect between this PTFE nano-fibrils network and that of the PBS domains on the foaming behaviour yielded higher cell densities and improved compressive performance of the foamed blends [428]. Blending PPC with PBAT is difficult due to the lack of compatibility, and so a compatibilizer agent can be used to improve compatibility. Using an epoxy-based chain extender and compatibilizing agent, foams of PPC/PBAT blends with a cell size of 15 μm and foam density of 0.083 g/cm^3^ were successfully prepared under high CO_2_ pressure foaming [215].

PCL is a semi-crystalline polyester with good toughness and ductileness and low toxicity to cell growth and proliferation. However, PCL has a low melt strength, which is not capable of sustaining the pore morphology during the microcellular foaming process. Therefore, PCL can be blended with other biodegradable polymers, such as PLA [133,462] or poly (lactic-co-glycolic acid) (PLGA) [80,463], in an effort to improve its melt strength. In this regard, Guo et al. [463] studied the foaming behaviour of PCL/PLGA blends and the effect of blend composition on the porous morphology, using solid-state batch foaming with supercritical CO_2_ as the blowing agent. They reported increased viscosity, which facilitates the foaming of PCL, decreased pore size, and both increased open-cell content and cell density with increased PLGA content. Xu et al. [462] prepared PLA/PCL/rice straw composite foams for wall insulation applications using azodicarbonamide as a blowing agent. They reported that the addition of 40 wt.% PCL improved both the impact and the compression strength. The water resistance of the foams was also improved by more than 800% and all foams showed an excellent insulation performance of about 0.040 w/(m·K).

As mentioned before, TPS shows two big drawbacks that undermine potential applications: it has poor mechanical properties and is strongly hydrophilic. Additionally, when using supercritical CO_2_ as the blowing agent, its poor melt strength makes it difficult for the biopolymer to hold the blowing agent within it, leading to the rapid diffusion of the gas out of the biopolymer, which generates starch foams with poor pore morphology. To overcome these issues, TPS is often blended with other biopolymers. PLA is an obvious candidate to complement the weaknesses of TPS due to its relatively good strength and modulus, thermal stability, and biodegradability, although PLA is hydrophobic, which may pose some compatibility issues. With this objective in mind, PLA has been blended with TPS [402,464,465]. Chauvet et al. [465] prepared PLA/TPS blends by melt extrusion and subjected the blends to extrusion foaming using supercritical CO_2_ as the blowing agent. They reported evenly foamed samples obtained from the blend made of 80 wt.% PLA and 20 wt% TPS, that showed high porosity (up to 96%) and a similar behaviour to neat PLA in terms of expansion and the type of porosity. On the other hand, samples obtained from the 50/50 (in wt.%) blend performed badly in terms of foaming, showing lower porosity and high cell coarsening due to the strong incompatibility between the two biopolymers. These results have shown that higher amounts of TPS in the blend would require the addition of a compatibilizing agent to improve the compatibility between the two biopolymers. Compatibilizing agents used to modify TPS and improve the interfacial adhesion between TPS and PLA include maleic anhydride. Studies have demonstrated that maleated TPS showed lower crystallinity and polarity and enhanced interaction with PLA, which resulted in improved barrier properties and improved mechanical performance of the PLA/TPS blends [466].

Other biopolymers, including PBAT [467], PCL [468], and PVA [421], can be used to improve TPS mechanical properties and circumvent poor foamability issues. Chang et al. [467] blended PBAT with TPS to improve the foamability of TPS by supercritical CO_2_ as a blowing agent. The TPS surface was modified using a compatibilizer, in the form of a silane with an epoxy group, to improve compatibility between TPS and PBAT. In addition, the use of the compatibilizing agent improved the melt strength of TPS and promoted the formation of intermolecular entanglement between PBAT and TPS that enabled the blend to withstand the cell morphology during the cell growth process. The reported results showed that the modified TPS/PBAT foams had a lower foam density (0.16 g/cm^3^) and better tensile properties compared to unmodified TPS/PBAT foams (foam density: 0.349 g/cm^3^). Torrejon et al. [469] blended TPS from different sources (tapioca and corn) to produce foams by mechanical foaming with gas injection. They reported foams with densities of nearly 45–50 kg/m^3^ and compression properties comparable to expanded polystyrene foams. PVA is a non-toxic, polar, and water-soluble synthetic polymer that is compatible with starch, which means that blending PVA and TPS produces a miscible one-phase blend. Kahvand et al. [421] investigated the effect of PVA and glycerol/water plasticizer contents on the mechanical properties and on the pore morphology of TPS/PVA blend foams produced by extrusion foaming. They reported a lower foam density, higher pore density, and a more uniform pore structure in the TPS/PVA blended foams compared to the TPS foam.

## 5. Circular Economy and Market Aspects of Biodegradable Foams’ Production

Plastics are unique useful materials, playing a key role in the global economy; however the continuing paradigm of extracting natural resources to support society’s increasing demands via global linear production and consumption is becoming an impossibility to sustain [470]. Currently, most parts of the recycled plastics are not going to the recycling facilities and bioplastics are expected to follow suit as well. According to statistics, 79% of all plastic waste ends up in landfills, promoting microplastic and marine pollution, where the burning of plastic garbage also has a negative effect on both the environment and human health [471]. The global industrialization and high dependence on non-renewable energy sources have led to an increase in solid waste and climate change, demanding for strategies to implement a circular economy in all sectors to reduce carbon emissions in 45% by 2030 and to achieve carbon neutrality by 2050 [472,473,474,475]. The Circular Economy Action Plan presented by the European Commission (EC) in 2020 [476] points to the directions towards which the economic model is being developed. Briefly, the products should be designed to be reusable and recyclable or more durable. Packaging materials should be reduced, restricted to certain applications, and designed to be recyclable. Restrictions on the creation of single-use items are also necessary. To encourage circularity in industry, the document also suggests giving the bio-based sector additional support. However, the report and other studies also note that there are emerging challenges regarding the sourcing, labelling, and use of bio-based, biodegradable, and compostable plastics [29,476]. The EC also suggests that the policy will support, via financial and regulatory incentives, the growth of the bioplastics industry, as one way to move towards a low-carbon economy [476]. Thus, there is a clear increasing concern regarding the environmental impact of plastic waste and related emissions of greenhouse gases motivating the transition to a more effective circular plastic economy [28,470,473]. Furthermore, in the framework of a circular economy model, the use of non-renewable resources and waste production can be minimized through the careful design of new products and the use of new industrial process, while reuse and recycling dominate the life cycles of materials. Bio-based, compostable, and biodegradable plastics are examples of alternative polymers that could be a more environmentally friendly option than fossil fuel-derived, non-biodegradable plastics. When compared to standard foams derived from fossil fuels, bio-degradable foam materials based on biopolymer matrices exhibit comparable physical properties and are more environmentally friendly with a lower carbon footprint, contributing to a sustainable development [28,477].

Increasing awareness about environmental conservation and the negative effects of non-biodegradable waste leads to an increase in demand for bio-based and biodegradable products. Innovations in biodegradable foam materials as shown in this review have led to the development of high-quality biodegradable foams with properties similar to the fossil-based polymers. Currently, biodegradable materials such as polylactic acid (PLA) or starch-based polymers offer a sustainable alternative to conventional foam products, accelerating the market growth [478]. Despite all the benefits of bio-based and biodegradable products, they also have trade-offs and sustainability issues of their own that need to be properly evaluated [478,479,480].

The main obstacles are primarily related to the economic aspects of bio-based foams’ production costs, since biodegradable foams often have higher production costs as compared to commodity foam materials, being more expensive for end users. Another barrier is the limitation in terms of their physical properties, since those polymers may have lower strength, reduced heat resistance, or limited durability for some specific uses, compromising the adoption in industries where specific performance requirements must be accomplished. The availability of raw materials for biodegradable foams is also mentioned as a constraint, since manufacturers may find it difficult to secure a sustainable supply of raw materials because of factors including seasonal variations, land use rivalry, and fluctuations in agricultural productivity. Considering recycling and waste management, a proper disposal or composting infrastructure is also needed for collecting, sorting, and processing biodegradable foam waste as it already exists for commodity plastics. Therefore, insufficient recycling and waste management systems may restrict biodegradable foams’ end-of-life options and impede their sustainable disposal [470,478]. On the consumer and plastic converters side, “greenwashing” information on environmental and sustainability issues related to biopolymers is being passed around, leading to inconsistent labelling and contradicting life cycle assessment that can be overpassed with the establishment of consistent information and global identification standards [28]. Regarding the need of sustainable products, it is also critical to dissipate any unfavourable perceptions about polymers that would discourage engineers, researchers, and designers from employing commodity plastics; in certain circumstances, though, those polymers might be preferable in terms of establishing a closed-loop system for certain applications. Thus, biodegradable and non-biodegradable plastic wastes can contribute to a circular plastic economy, if used properly.

Considering the economic aspect, the global biodegradable foam market was valued at USD 906.1 Mn in 2023 and it is expected to exhibit a CAGR of 10.5% over the forecast period of 2023 to 2030, as highlighted in the report published by Coherent Market Insights [478]. The North of America represents a market share of 29%, followed by Europe with 23% and Asia Pacific with 16%. The market-drivers for the growing demand in those regions are mostly related to the increasing awareness about the environmental impact of non-biodegradable foams, growing demand of producers for biodegradable food and beverage packaging solutions from natural resources, and the increasing focus on sustainability, leading to government regulations under those product solutions. Globally, it can be stated that the worldwide foam market is prepared to provide cost-effective and sustainable solutions that are compostable and biodegradable alternatives to the traditional polymer foams.

## 6. Conclusions and Future Perspectives

The increase in environmental awareness has served as a motivation to change the plastics industry paradigm to become more sustainable. Intensive research has been carried out to replace non-biodegradable petrochemical-based plastic materials with biodegradable materials from renewable sources. Polymeric foams are lightweight materials with improved properties such as low density, good thermal and sound insulation, electromagnetic shielding, and high specific strength compared to fully solid polymeric materials. Foams have increasingly found practical applications such as in insulation, packaging, medicine, construction, automotive industry, and aeronautics, among others.

Many developments in foaming research are carried out using batch foaming on a small scale. However, from the point of view of practical application and production on an industrial scale, many of the outcomes of that research would gain immensely if they were converted to a continuous process, such as extrusion foaming or foam injection moulding. It is therefore necessary to develop technologies and processes that allow for the transfer of knowledge from batch foaming to continuous foaming processes. This is more important considering that the practical application of these foams based on biodegradable polymers depends on the collaboration with industry. Moreover, athe industry will only be interested in materials and technologies that can be used in industrial continuous processes.

In situ foam 3D printing initially involved printing previously saturated filaments. Using this approach, the main issue is the ability of the polymer to retain the dissolved expansion gas (PBA) without loss until the printing stage, given that in the case of biodegradable polymers, especially in view of biomedical applications, the use of CBAs is not interesting due to toxicity problems. As the retention of the expansion gas in various polymers depends to a large extent on their molecular structure, the application of in situ 3D printing will be strongly linked to research in polymer chain extension, crosslinking, and branching methods in those materials where the technique is feasible. The other 3D printing technique reviewed, the direct printing of a saturated polymer/gas mixture, depends greatly on the modification of existing machinery used in common 3D printing to enable foaming in the die nozzle, similar to what occurs now in extrusion foaming. The expansion gas would be injected into the extruder barrel of the printer, and so it would not be necessary for the polymer to have a large capacity in retaining the expansion agent, thus enabling a great expansion of the range of materials that could be used in this technique.

The control of the porosity structure in foams has advanced recently with the development of the multi-step foaming process, which has been applied successfully in the production of bimodal foams for biomedical applications. This technology relies on a two-step pressurization/depressurization process that allows for controlling the pore nucleation and growth processes and thus providing foams with tailored morphology. This multi-step process has also been combined with a heating/cooling step, by increasing or decreasing the temperature during CO_2_ saturation at high pressure to control polymer recrystallization, to improve the mechanical properties of the foams.

The application of ultrasonication proved to be effective in improving the nucleation processes and enabling the formation of an interconnected open-cell structure, with tailored structures being obtained depending on applying the ultrasonication at the beginning or after the nucleation. Much research will be needed to apply this technique to the many available biodegradable polymers to improve the cell density, cell structure uniformity, and expansion ratio of the foams and ultimately extend the potential applications of the foams. Another feature that would be interesting to investigate would be to relate the properties of biopolymers to their responses to ultrasound, so that it could be possible to modify the polymer structure regarding its desired response to ultrasound and obtain foams with customized micellar structures.

Regarding the different biodegradable polymers, as this review shows, many efforts have been made to address the many limitations that these materials present during the foaming process. Foaming processes are complex and vary greatly from polymer to polymer, as foaming processes depend greatly on the rheological and thermal properties of polymers and these properties are strongly dependent on the structure of the material.

Melt strength is the main factor that limits the use of biodegradable polymers in foaming. To circumvent this issue, a great variety of chemicals has been investigated with the aim of increasing the molecular weight and producing modifications in the polymer structure, such as branching or crosslinking to improve the rheological behaviour to enable foaming. Depending on the biodegradable polymers, several strategies to improve the foam morphology were reported and reviewed. Changes in the properties of biodegradable polymers and in their crystallization behaviour make it possible to expand the number of techniques that can be applied to each biodegradable polymer and the range of foam morphologies that can be obtained, allowing in this way the gradual replacement of petroleum-based polymers by their biodegradable counterparts, and so, this issue should be further researched.

The use of supercritical CO_2_ has been increasingly used in foaming; however, not all biodegradable polymers are suitable for CO_2_ foaming due to the low melt strength of the polymer or the low solubility of CO_2_ in the polymer. These problems can be overcome once again by changing the structure of the polymer or blending it with other polymers.

The melt strength and nucleation of biodegradable polymers can also be improved by incorporating additives such as nucleating agents. These nucleating agents, by altering the melt strength, the crystallinity, and the solubility of blowing agents, allow for an improvement in the pore structure, such as the formation of bimodal pores, in addition to providing other desirable properties to the foam such as the electromagnetic shielding, which can be achieved by the addition of carbon nanotubes. Each type of additive or filler has specific outcomes depending on its properties and, therefore, it is important to determine the best additive for each polymer, considering the biodegradable polymer and characteristics of the foam to be obtained. In addition, the effect of the additive on the foaming process and in the resulting foam morphology needs to be further explored and understood.

In addition to improving the characteristics of the biodegradable polymers to be foamed, whether by chemical means or by simple blending with other biodegradable polymers, the foaming process can also be improved by adjusting the process conditions, and therefore they also deserve to be studied. This combination of parameters can be studied using theoretical modelling and computer simulation combined with statistical tools such as Response Surface Methodology to optimize the factors that allow for obtaining the best results in terms of foam performance.

The development of biodegradable polymer-based materials with high porosity and adjustable morphology in terms of pore size and density considerably expands the areas of use of these materials. This development has been achieved through the improvement in techniques that allow for the development of these porous structures. One area that has received major attention is the area of medical devices, which generally involve the use of polymers of natural origin and that are preferably biodegradable. Monitoring the morphology of these porous materials based on biodegradable polymers opens new possibilities for application in the biomedical area, such as in scaffolds, or in other areas such as smart packaging, microfiltration and separation of solvents, breathable textile articles, and pollutant sorbent materials, among other applications.

Although foam based on biodegradable polymers already finds some applications, namely TPS in disposable packaging, certain properties of these foamed materials need to be improved so that they can successfully replace foamed polymers of petrochemical origin at a large scale. In addition, although consumers are increasingly aware of environmental problems and gradually develop a positive behaviour towards sustainability and concomitantly prefer sustainable products, the higher price of biodegradable polymers often drives consumers away. To improve the interest of the consumers, it is necessary to increase the scale of production of biodegradable materials and their application in products, so that the price to the consumer can be lowered, combined with consumer awareness campaigns so that even if the price is slightly superior to the corresponding petroleum-based product, the consumers still prefer to buy the sustainable one.

From an environmental point of view, biodegradable foams have advantages over compact biodegradable materials. Not only do they use less material to produce a product of similar dimensions, but biodegradable polymer foams have higher degradation rates than the corresponding non-porous biodegradable materials. Foam morphology also influences foam degradation, as open cells and foams with larger cells facilitate foam degradation. Nevertheless, the issue of the degradation of biodegradable polymer foams has not received much attention in research. Given the importance of sustainability in the global agenda, more research into foam degradation is needed.

Plastic waste, especially that resulting from disposable plastic packaging, is currently considered one of the main environmental problems given the significant increase in plastic waste that accumulates in the oceans. The replacement of materials of petrochemical origin with materials of sustainable origin, as well as their biodegradation, is considered a viable and urgent solution to combat environmental ruin. Research should focus on the objective of ensuring that, in the future, biodegradable polymer-based materials can successfully replace traditional polymers manufactured from fossil resources, preferably aligned with principles of circular economy. Certain problems need to be resolved. For example, TPS is highly sensitive to moisture, which limits its use in packaging. To improve this and other aspects, TPS has been mixed with a variety of additives in order to improve its properties, such as improving the resistance against moisture, microbes, or degradation caused by UV rays, or even to increase the barrier properties against oxygen migration. Despite this, it is necessary to increase consumer awareness so that they are willing to accept a product that may be a little more expensive or of lower quality but that is more sustainable and environmentally friendly. This should not be difficult in the case of packaging or single-use disposable products where quality and appearance are less important as the useful life of the material is very short.

In short, although the foaming process has been known for several years and has been successfully applied to biodegradable polymers, it will remain a vivid area of research in the coming years.

## Figures and Tables

**Table 5:** Representative studies that have been reported for PHA foams.

Objectives	Foaming Conditions	Key Features	Ref.
Study the effects of the rheological behaviour of the polymer, the nucleation agent, the blowing agent, and the processing conditions on the foam density and structure.	Extrusion foaming using sodium bicarbonate and citric acid as CBAs and calcium carbonate as a nucleation agent.	Thermal and hydrolytic degradations observed when using a water-generating blowing agent. Extruded foams showed mostly closed-cell morphology with cell sizes ranging between 50 and 200 mm. Lower screw speed resulted in higher expansion of the foams due to higher residence time for the decomposition of the CBA. Addition of calcium carbonate yielded a much finer cell structure, although at the cost of an increase in the density of the foams.	[354]
Prepare PHBV/organo-clays nano-biocomposite foams by extrusion foaming.	Extrusion foaming using supercritical CO_2_.	Previous preparation of a masterbatch by twin-screw extrusion and its dilution during the foaming process improved clay dispersion without widespread thermal degradation of PHBV. Good clay dispersion favoured homogeneous nucleation while restraining pore coalescence, yielding more homogeneous nano-biocomposite foams and higher porosity up to 50%.	[360]
Study the crystallization and foaming behaviour, thermal stability, and degradation of PHBV/nanofibrillated cellulose (NFC) biodegradable nanocomposites.	Batch foaming experiments using CO_2_ as the PBA. Foaming of CO_2_-saturated samples was triggered by a rapid pressure drop and a rapid temperature increase (in a hot oil bath).	NFC served as a nucleating agent, enabling the early onset of crystallization. Higher amounts of NFC led to extended thermal degradation of the PHBV matrix and to lower solubility and increased desorption diffusivity of CO_2_ in the PHBV/NFC nanocomposites. Generally, addition of NFC showed an inhibiting behaviour of the foaming due to lower CO_2_ sorption, faster CO_2_ loss, and a higher degree of crystallinity.	[361]
Development of PHBV/graphene nanoplates (GNPs) with electromagnetic shielding effect.	Batch foaming in a high-pressure autoclave using supercritical CO_2_ as the PBA. Samples were saturated at 160 °C and 20 MPa. Foaming triggered by pressure drop at 140 °C.	Increasing GNP content from 0 to 6 wt.% resulted in a decreased pore size and volume expansion ratio of PHBV foams. GNPs improved the electrical conductivity by 12 orders of magnitude. EMI shielding effectiveness reached 27.4 dB cm^3^/g.	[359]
Preparation of Poly(3-hydroxybutyrate-co-3-hydroxyhexanoate) (PHBH) microcellular foams by using melt memory effect.	Foam injection moulding (FIM) using nitrogen as a physical blowing agent. A core-back operation was used to promote the foamability and control the cell size and density of the foams.	Cell density, uniformity of the cellular structure, and higher ductility of PHBH microcellular foam, obtained at a melting temperature of 150 °C. The polymer melt memory effect was maintained for the melting temperature in the range 150–160 °C, providing cell nucleation sites through the crystals in foams.	[126]

**Table 6:** Representative studies that have been reported for starch foams.

Objectives	Foaming Conditions	Key Features	Ref.
Study the biodegradability and application of starch-based foams incorporated with grape stalks for packaging foods with low moisture content.	Grape stalks, guar gum, magnesium stearate, and water (55 wt%) were mixed with cassava starch (32%wt) and glycerol (5% wt). The resulting mixture was poured in a Teflon-coated metal mould and compression moulded in a heated hydraulic press at 70 bar and 180 °C for 7 min.	Loss of crystallinity of starch after expansion (an amorphous material was formed). Foams were completely biodegraded after 7 weeks. Foams of starch/grape stalks showed good properties in the applicability test.	[406]
Improve the expansion capability of starch foam by chemical modification of starch via oxidation.	The paste containing starch, water, and other additives was placed in a mould coated with Teflon and compression moulded at 190 °C for 40 min.	The intrinsic viscosity of the starch solutions decreased as the degree of oxidation increased, indicating a molecular weight reduction in the starch due to chain scission caused by oxidation. Number of C-H and C-O bonds decrease due to the conversion of CH_2_OH-6 into carboxyl groups. Foams from the modified starches showed decreased density (142 kg/m^3^) compared to foams from regular starch (308 kg/m^3^).	[391]
Evaluate the effect of thyme (TEO) or oregano (OEO) essential oil on the physical and antimicrobial properties of foams based on native sweet potato starch.	Thermocompression. Sweet potato starch, water, TEO or OEO, plasticizer (glycerol), and the release agent (magnesium stearate) were mixed for 10 min. The batter was subsequently thermocompressed at 160 °C and 60 bar for 10 min.	Observed an antimicrobial effect caused by small essential oil drops trapped in the first layers of the foams. The foams with OEO showed a higher antimicrobial effect than the foams with TEO. The presence of the essential oils reduced the solubility and water absorption of the starch matrix.	[422]
Investigate the feasibility of starch-based composite foam (SCF) as fresh chicken meat packaging during refrigerated storage.	Starch pellets were prepared by melt mixing potato starch, maleic anhydride, and glycerol at 130 °C in a twin-screw extruder. Afterwards, starch pellets, PLA, PVA, nanoclay, and azodicarbonamide (CBA) were melt-blended and foamed in a twin-screw extruder at 160 °C. Foams with 2% nisin as an antimicrobial agent were also prepared.	The starch-based foam showed higher liquid retention capacity than expanded polystyrene, with no impairment of chicken meat quality during refrigeration storage. The incorporation of 2% of nisin into the starch-based composite foam resulted in a lower microbiological growth in the chicken meat during storage.	[423]
Fabrication of starch-based composite foams (with natural reinforcements, such as barley straw fibres, grape wastes, and cardoon wastes) by microwave radiation.	In the first step, starch was plasticized with water by extrusion; in the second step, pellets were thermoformed into sheets; in the third step, the starch sheets were foamed by microwave radiation.	The natural reinforcements increased the rigidity, strength, and the toughness of the foams. During foaming, the flexible and solid thermoplastic starch sheet turned into a rigid foam. The use of barley straw fibres and cardoon waste showed a decrease in the average cell size while the addition of grape particles did not change the average cell size of foams.	[163]

## Abbreviations

ACNC—Acetylated cellulose nanocrystals; AKD—Alkyl ketene dimer; AM—Additive manufacturing; CAGR—Compound annual growth rate; CB—Carbon black; CBA—Chemical blowing agent; ChNC—Chitin nanocrystals; CNC—cellulose nanocrystals; CNF—Cellulose nanofibers; CNTs—Carbon nanotubes; EMI—Electromagnetic interference; EPP—Expandable polypropylene; EPS—Expandable polystyrene; FDM—Fused deposition modelling; FIM—Foam injection moulding; GNPs—Graphene nanoplatelets; MJF—Multi-jet fusion; MWCNTs—multi-walled carbon nanotubes; NFC—Nanofibrillated cellulose; NTP—Novatein^®^ Thermoplastic Protein; OMMT—Organic montmorillonite; P(3HB-co-4HB)—Poly(3-hydroxybutyrate-co-4-hydroxybutyrate); P4HB—Poly 4-hydroxybutyrate; PBA—Physical blowing agent; PBAT—Polybutylene adipate-co-terephthalate; PBS—Poly (butylene succinate); PBSA—Poly (butylene succinate-co-butylene adipate); PBST—Poly (butylene succinate-butylene terephthalate); PCL—Poly (caprolactone); PDLA—Poly (D-lactic acid); PE—Polyethylene; PEG—Poly (ethylene glycol); PET—Polyethylene terephthalate; PHA—Poly-hydroxialkanoates; PHBV—Poly 3-hydroxybutyrate-co-3-hydroxyvalerate; PHBHHx—Poly (3-hydroxybutyrate-co-hydroxyhexanoate); PHV—Polyhydroxyvalerate; PLA—Poly (lactic acid); PLCL—Poly (L-lactide-co-ε-caprolactone); PLGA—Poly (lactic-co-glycolic acid); PLLA—Poly (L-lactic acid); PEO—Poly (ethylene oxide); PP—Polypropylene; PPC—Poly (propylene carbonate); PTFE—Polytetrafluoroethylene; PVA—Poly (vinyl alcohol) PVA; SAG—Styrene-acrylonitrile-glycidyl methacrylate terpolymer; SEM—Scanning electron microscope; SLS—Selective laser sintering; Tg—Glass transition temperature; TPS—Thermoplastic starch; VBGE—4-vinylbenzyl glycidyl ether.

## References

[1] Chauvet M., Sauceau M., Fages J. (2017). Extrusion assisted by supercritical CO_2_: A review on its application to biopolymers. J. Supercrit. Fluids.

[2] Yan Z., Liao X., He G., Li S., Guo F., Zou F., Li G. (2020). Green and High-Expansion PLLA/PDLA Foams with Excellent Thermal Insulation and Enhanced Compressive Properties. Ind. Eng. Chem. Res..

[3] Wang G., Zhao G., Wang S., Zhang L., Park C.B. (2018). Injection-molded microcellular PLA/graphite nanocomposites with dramatically enhanced mechanical and electrical properties for ultra-efficient EMI shielding applications. J. Mater. Chem. C.

[4] Yin D., Mi J., Zhou H., Wang X., Tian H. (2020). Fabrication of branching poly (butylene succinate)/cellulose nanocrystal foams with exceptional thermal insulation. Carbohydr. Polym..

[5] Forest C., Chaumont P., Cassagnau P., Swoboda B., Sonntag P. (2015). Polymer nano-foams for insulating applications prepared from CO_2_ foaming. Prog. Polym. Sci..

[6] Mort R., Vorst K., Curtzwiler G., Jiang S. (2021). Biobased foams for thermal insulation: Material selection, processing, modelling, and performance. RSC Adv..

[7] Tapia-Blácido D.R., Aguilar G.J., de Andrade M.T., Rodrigues-Júnior M.F., Guareschi-Martins F.C. (2022). Trends and challenges of starch-based foams for use as food packaging and food container. Trends Food Sci. Technol..

[8] Faba S., Arrieta M.P., Agüero Á., Torres A., Romero J., Rojas A., Galotto M.J. (2022). Processing Compostable PLA/Organoclay Bionanocomposite Foams by Supercritical CO_2_ Foaming for Sustainable Food Packaging. Polymers.

[9] Wang Y., Guo F., Liao X., Li S., Yan Z., Zou F., Peng Q., Li G. (2023). High-expansion-ratio PLLA/PDLA/HNT composite foams with good thermally insulating property and enhanced compression performance via supercritical CO_2_. Int. J. Biol. Macromol..

[10] Vorawongsagul S., Pratumpong P., Pechyen C. (2021). Preparation and foaming behavior of poly (lactic acid)/poly (butylene succinate)/cellulose fiber composite for hot cups packaging application. Food Packag. Shelf Life.

[11] Shaikh S., Yaqoob M., Aggarwal P. (2021). An overview of biodegradable packaging in food industry. Curr. Res. Food Sci..

[12] Wang J., Zhang Y., Sun J., Jiao Z. (2022). Controllable fabrication of multi-modal porous PLGA scaffolds with different sizes of SPIONs using supercritical CO_2_ foaming. J. Appl. Polym. Sci..

[13] Silva S.S., Rodrigues L.C., Fernandes E.M., Reis R.L., de Moraes M.A., da Silva C.F., Vieira R.S. (2020). Chapter 1—Fundamentals on biopolymers and global demand. Biopolymer Membranes and Films.

[14] Duarte R.M., Correia-Pinto J., Reis R.L., Duarte A.R.C. (2018). Subcritical carbon dioxide foaming of polycaprolactone for bone tissue regeneration. J. Supercrit. Fluids.

[15] Ju J., Gu Z., Liu X., Zhang S., Peng X., Kuang T. (2020). Fabrication of bimodal open-porous poly (butylene succinate)/cellulose nanocrystals composite scaffolds for tissue engineering application. Int. J. Biol. Macromol..

[16] Santos-Rosales V., Gallo M., Jaeger P., Alvarez-Lorenzo C., Gómez-Amoza J.L., García-González C.A. (2020). New insights in the morphological characterization and modelling of poly(ε-caprolactone) bone scaffolds obtained by supercritical CO_2_ foaming. J. Supercrit. Fluids.

[17] Duarte R.M., Correia-Pinto J., Reis R.L., Duarte A.R.C. (2020). Advancing spinal fusion: Interbody stabilization by in situ foaming of a chemically modified polycaprolactone. J. Tissue Eng. Regen. Med..

[18] Silva S.S., Fernandes E.M., Pina S., Silva-Correia J., Vieira S., Oliveira J.M., Reis R.L., Ducheyne P. (2017). 2.11 Polymers of Biological Origin. Comprehensive Biomaterials II.

[19] Rokkonen T., Peltola H., Sandquist D. (2019). Foamability and viscosity behavior of extrusion foamed PLA–pulp fiber biocomposites. J. Appl. Polym. Sci..

[20] Standau T., Castellón S.M., Delavoie A., Bonten C., Altstädt V. (2019). Effects of chemical modifications on the rheological and the expansion behavior of polylactide (PLA) in foam extrusion. E-Polymers.

[21] Kim D., Hikima Y., Ohshima M. (2022). Millefeuille-like cellular structures of biopolymer blend foams prepared by the foam injection molding technique. J. Appl. Polym. Sci..

[22] Zhang X., Ding W., Chang E., Chen X., Chen J., Park C.B., Shen C. (2020). Foaming Behaviors and Mechanical Properties of Injection-Molded Polylactide/Cotton-Fiber Composites. Ind. Eng. Chem. Res..

[23] Zhao S., Malfait W.J., Guerrero-Alburquerque N., Koebel M.M., Nyström G. (2018). Biopolymer Aerogels and Foams: Chemistry, Properties, and Applications. Angew. Chem.-Int. Ed..

[24] Gama N., Ferreira A., Barros-Timmons A. (2018). Polyurethane Foams: Past, Present, and Future. Materials.

[25] Kumari S.V.G., Pakshirajan K., Pugazhenthi G. (2022). Recent advances and future prospects of cellulose, starch, chitosan, polylactic acid and polyhydroxyalkanoates for sustainable food packaging applications. Int. J. Biol. Macromol..

[26] Awasthi S.K., Kumar M., Kumar V., Sarsaiya S., Anerao P., Ghosh P., Singh L., Liu H., Zhang Z., Awasthi M.K. (2022). A comprehensive review on recent advancements in biodegradation and sustainable management of biopolymers. Environ. Pollut..

[27] Iwata T. (2015). Biodegradable and Bio-Based Polymers: Future Prospects of Eco-Friendly Plastics. Angew. Chem. Int. Ed..

[28] Rosenboom J.-G., Langer R., Traverso G. (2022). Bioplastics for a circular economy. Nat. Rev. Mater..

[29] Di Bartolo A., Infurna G., Dintcheva N.T. (2021). A Review of Bioplastics and Their Adoption in the Circular Economy. Polymers.

[30] Pellis A., Malinconico M., Guarneri A., Gardossi L. (2021). Renewable polymers and plastics: Performance beyond the green. New Biotechnol..

[31] Lambert S., Wagner M. (2017). Environmental performance of bio-based and biodegradable plastics: The road ahead. Chem. Soc. Rev..

[32] Haider T.P., Völker C., Kramm J., Landfester K., Wurm F.R. (2019). Plastics of the Future? The Impact of Biodegradable Polymers on the Environment and on Society. Angew. Chem.-Int. Ed..

[33] de França J.O.C., da Silva Valadares D., Paiva M.F., Dias S.C.L., Dias J.A. (2022). Polymers Based on PLA from Synthesis Using D,L-Lactic Acid (or Racemic Lactide) and Some Biomedical Applications: A Short Review. Polymers.

[34] Labet M., Thielemans W. (2009). Synthesis of polycaprolactone: A review. Chem. Soc. Rev..

[35] Abdullah Z.W., Dong Y., Davies I.J., Barbhuiya S. (2017). PVA, PVA Blends, and Their Nanocomposites for Biodegradable Packaging Application. Polym.-Plast. Technol. Eng..

[36] Bangar S.P., Whiteside W.S., Ashogbon A.O., Kumar M. (2021). Recent advances in thermoplastic starches for food packaging: A review. Food Packag. Shelf Life.

[37] European Bioplastics (2023). Bioplastics Market. https://www.european-bioplastics.org/bioplastics-market-development-update-2023-2/.

[38] Standau T., Zhao C., Murillo Castellón S., Bonten C., Altstädt V. (2019). Chemical Modification and Foam Processing of Polylactide (PLA). Polymers.

[39] Farhanmoghaddam F., Javadi A. (2020). Fabrication of poly (lactic acid) foams using supercritical nitrogen. Cell. Polym..

[40] Kmetty Á., Litauszki K. (2020). Development of Poly (Lactide Acid) Foams with Thermally Expandable Microspheres. Polymers.

[41] Nofar M., Utz J., Geis N., Altstädt V., Ruckdäschel H. (2022). Foam 3D Printing of Thermoplastics: A Symbiosis of Additive Manufacturing and Foaming Technology. Adv. Sci..

[42] Di Maio E., Kiran E. (2018). Foaming of polymers with supercritical fluids and perspectives on the current knowledge gaps and challenges. J. Supercrit. Fluids.

[43] Xue K., Chen P., Yang C., Xu Z., Zhao L., Hu D. (2022). Low-shrinkage biodegradable PBST/PBS foams fabricated by microcellular foaming using CO_2_ & N_2_ as co-blowing agents. Polym. Degrad. Stab..

[44] Boonprasertpoh A., Pentrakoon D., Junkasem J. (2017). Effect of crosslinking agent and branching agent on morphological and physical properties of poly(butylene succinate) foams. Cell. Polym..

[45] Xia B., Wang Y., Jiang J., Zhang X., Li T., Ma P., Chen M., Dong W. (2022). Effects of dicumyl peroxide on cross-linking pure poly(butylene succinate) foaming materials for high expansion and high mechanical strength. Polym. Adv. Technol..

[46] Tsui A., Wright Z., Frank C.W. (2014). Prediction of gas solubility in poly(3-hydroxybutyrate-*co*-3-hydroxyvalerate) melt to inform process design and resulting foam microstructure. Polym. Eng. Sci..

[47] Osman M.A., Virgilio N., Rouabhia M., Lorenzo L.E., Mighri F. (2023). A novel foaming technique to develop functional open-cell polylactic acid scaffolds for bone tissue engineering. J. Appl. Polym. Sci..

[48] Guan L.T., Du F.G., Wang G.Z., Chen Y.K., Xiao M., Wang S.J., Meng Y.Z. (2007). Foaming and chain extension of completely biodegradable poly(propylene carbonate) using DPT as blowing agent. J. Polym. Res..

[49] Guan L.T., Xiao M., Meng Y.Z., Li R.K.Y. (2006). Chemically foaming of biodegradable poly(propylene carbonate) derived from carbon dioxide and propylene oxide. Polym. Eng. Sci..

[50] Villamil Jiménez J.A., Le Moigne N., Bénézet J.-C., Sauceau M., Sescousse R., Fages J. (2020). Foaming of PLA Composites by Supercritical Fluid-Assisted Processes: A Review. Molecules.

[51] Dugad R., Radhakrishna G., Gandhi A. (2020). Recent advancements in manufacturing technologies of microcellular polymers: A review. J. Polym. Res..

[52] Nofar M., Park C.B. (2014). Poly (lactic acid) foaming. Prog. Polym. Sci..

[53] Li G., Li H., Turng L.S., Gong S., Zhang C. (2006). Measurement of gas solubility and diffusivity in polylactide. Fluid Phase Equilibria.

[54] Sato Y., Takikawa T., Takishima S., Masuoka H. (2001). Solubilities and diffusion coefficients of carbon dioxide in poly(vinyl acetate) and polystyrene. J. Supercrit. Fluids.

[55] Zhang Q., Xanthos M., Dey S.K. (2001). Parameters affecting the in-line measurement of gas solubility in thermoplastic melts during foam extrusion. J. Cell. Plast..

[56] Colton J.S., Suh N.P. (1987). Nucleation of microcellular foam: Theory and practice. Polym. Eng. Sci..

[57] Pang Y., Cao Y., Zheng W., Park C.B. (2022). A comprehensive review of cell structure variation and general rules for polymer microcellular foams. Chem. Eng. J..

[58] Raps D., Hossieny N., Park C.B., Altstädt V. (2015). Past and present developments in polymer bead foams and bead foaming technology. Polymer.

[59] Leung S.N., Wong A., Wang L.C., Park C.B. (2012). Mechanism of extensional stress-induced cell formation in polymeric foaming processes with the presence of nucleating agents. J. Supercrit. Fluids.

[60] Spitael P., Macosko C.W. (2004). Strain hardening in polypropylenes and its role in extrusion foaming. Polym. Eng. Sci..

[61] Henriques I.R., Rouleau L., Castello D.A., Borges L.A., Deü J.F. (2020). Viscoelastic behavior of polymeric foams: Experiments and modeling. Mech. Mater..

[62] Chen X., Feng J.J., Bertelo C.A. (2006). Plasticization effects on bubble growth during polymer foaming. Polym. Eng. Sci..

[63] Gabriel C., Münstedt H. (2003). Strain hardening of various polyolefins in uniaxial elongational flow. J. Rheol..

[64] Stadler F.J., Nishioka A., Stange J., Koyama K., Münstedt H. (2007). Comparison of the elongational behavior of various polyolefins in uniaxial and equibiaxial flows. Rheol. Acta.

[65] Münstedt H. (2011). Rheological properties and molecular structure of polymer melts. Soft Matter.

[66] Tsuchiya A., Tateyama H., Kikuchi T., Takahashi T., Koyama K. (2007). Influence of filler types and contents on foaming structures in ABS microcellular foams. Polym. J..

[67] Tammaro D., Villone M.M., D’Avino G., Maffettone P.L. (2022). An Experimental and Numerical Investigation on Bubble Growth in Polymeric Foams. Entropy.

[68] Kiran E. (2010). Foaming strategies for bioabsorbable polymers in supercritical fluid mixtures. Part II. Foaming of poly(ε-caprolactone-co-lactide) in carbon dioxide and carbon dioxide + acetone fluid mixtures and formation of tubular foams via solution extrusion. J. Supercrit. Fluids.

[69] Khademi S.M.H., Hemmati F., Aroon M.A. (2020). An insight into different phenomena involved in continuous extrusion foaming of biodegradable poly(lactic acid)/expanded graphite nanocomposites. Int. J. Biol. Macromol..

[70] Kosowska K., Krzysztoforski J., Henczka M. (2022). Foaming of PCL-Based Composites Using scCO_2_—Biocompatibility and Evaluation for Biomedical Applications. Materials.

[71] Santos-Rosales V., Ardao I., Goimil L., Gomez-Amoza J.L., García-González C.A. (2021). Solvent-Free Processing of Drug-Loaded Poly(ε-Caprolactone) Scaffolds with Tunable Macroporosity by Combination of Supercritical Foaming and Thermal Porogen Leaching. Polymers.

[72] Borkotoky S.S., Chakraborty G., Katiyar V. (2018). Thermal degradation behaviour and crystallization kinetics of poly (lactic acid) and cellulose nanocrystals (CNC) based microcellular composite foams. Int. J. Biol. Macromol..

[73] Sawicka K., Kosowska K., Henczka M. (2019). Application of porogenes in production of porous polymers by supercritical foaming. Chem. Process Eng.-Inz. Chem. Proces..

[74] Jing X., Mi H.-Y., Cordie T., Salick M., Peng X.-F., Turng L.-S. (2014). Fabrication of Porous Poly(ε-caprolactone) Scaffolds Containing Chitosan Nanofibers by Combining Extrusion Foaming, Leaching, and Freeze-Drying Methods. Ind. Eng. Chem. Res..

[75] Zhang W., Chen B., Zhao H., Yu P., Fu D., Wen J., Peng X. (2013). Processing and characterization of supercritical CO_2_ batch foamed poly(lactic acid)/poly(ethylene glycol) scaffold for tissue engineering application. J. Appl. Polym. Sci..

[76] Nofar M., Tabatabaei A., Ameli A., Park C.B. (2013). Comparison of melting and crystallization behaviors of polylactide under high-pressure CO_2_, N_2_, and He. Polymer.

[77] Frerich S.C. (2015). Biopolymer foaming with supercritical CO_2_—Thermodynamics, foaming behaviour and mechanical characteristics. J. Supercrit. Fluids.

[78] Longo A., Di Maio E., Di Lorenzo M.L. (2022). Heterogeneous Bubble Nucleation by Homogeneous Crystal Nuclei in Poly(L-Lactic Acid) Foaming. Macromol. Chem. Phys..

[79] Reignier J., Gendron R., Champagne M.F. (2007). Extrusion foaming of poly(lactic acid) blown with CO_2_: Toward 100% green material. Cell. Polym..

[80] Santos-Rosales V., Magariños B., Starbird R., Suárez-González J., Fariña J.B., Alvarez-Lorenzo C., García-González C.A. (2021). Supercritical CO_2_ technology for one-pot foaming and sterilization of polymeric scaffolds for bone regeneration. Int. J. Pharm..

[81] Lee L.J., Zeng C., Cao X., Han X., Shen J., Xu G. (2005). Polymer nanocomposite foams. Compos. Sci. Technol..

[82] Antunes M., Velasco J.I. (2014). Multifunctional polymer foams with carbon nanoparticles. Prog. Polym. Sci..

[83] Notario B., Pinto J., Rodriguez-Perez M.A. (2016). Nanoporous polymeric materials: A new class of materials with enhanced properties. Prog. Mater. Sci..

[84] Rizvi A., Chu R.K.M., Park C.B. (2018). Scalable Fabrication of Thermally Insulating Mechanically Resilient Hierarchically Porous Polymer Foams. ACS Appl. Mater. Interfaces.

[85] Buahom P., Wang C., Alshrah M., Wang G., Gong P., Tran M.P., Park C.B. (2020). Wrong expectation of superinsulation behavior from largely-expanded nanocellular foams. Nanoscale.

[86] Tiwary P., Park C.B., Kontopoulou M. (2017). Transition from microcellular to nanocellular PLA foams by controlling viscosity, branching and crystallization. Eur. Polym. J..

[87] Wang L., Lee R.E., Wang G., Chu R.K.M., Zhao J., Park C.B. (2017). Use of stereocomplex crystallites for fully-biobased microcellular low-density poly(lactic acid) foams for green packaging. Chem. Eng. J..

[88] Yu K., Ni J., Zhou H., Wang X., Mi J. (2020). Effects of in-situ crystallization on poly (lactic acid) microcellular foaming: Density functional theory and experiment. Polymer.

[89] Yu L., Toikka G., Dean K., Bateman S., Yuan Q., Filippou C., Nguyen T. (2013). Foaming behaviour and cell structure of poly(lactic acid) after various modifications. Polym. Int..

[90] Najafi N., Heuzey M.C., Carreau P.J., Therriault D., Park C.B. (2014). Rheological and foaming behavior of linear and branched polylactides. Rheol. Acta.

[91] Li Y., Mi J., Fu H., Zhou H., Wang X. (2019). Nanocellular Foaming Behaviors of Chain-Extended Poly(lactic acid) Induced by Isothermal Crystallization. ACS Omega.

[92] Nofar M. (2016). Effects of nano-/micro-sized additives and the corresponding induced crystallinity on the extrusion foaming behavior of PLA using supercritical CO_2_. Mater. Des..

[93] Ema Y., Ikeya M., Okamoto M. (2006). Foam processing and cellular structure of polylactide-based nanocomposites. Polymer.

[94] Keshtkar M., Nofar M., Park C.B., Carreau P.J. (2014). Extruded PLA/clay nanocomposite foams blown with supercritical CO_2_. Polymer.

[95] Sauceau M., Fages J., Common A., Nikitine C., Rodier E. (2011). New challenges in polymer foaming: A review of extrusion processes assisted by supercritical carbon dioxide. Prog. Polym. Sci..

[96] Kuhnigk J., Standau T., Dörr D., Brütting C., Altstädt V., Ruckdäschel H. (2022). Progress in the development of bead foams—A review. J. Cell. Plast..

[97] Park C.B., Baldwin D.F., Suh N.P. (1995). Effect of the pressure drop rate on cell nucleation in continuous processing of microcellular polymers. Polym. Eng. Sci..

[98] Duborper C., Samuel C., Akue-Asseko A.C., Loux C., Lacrampe M.F., Krawczak P. (2018). Design of biobased poly(butylene succinate) foams by single-screw extrusion: Identification of relevant rheological parameters controlling foam morphologies. Polym. Eng. Sci..

[99] Okolieocha C., Raps D., Subramaniam K., Altstädt V. (2015). Microcellular to nanocellular polymer foams: Progress (2004-2015) and future directions—A review. Eur. Polym. J..

[100] Lee R.E., Guo Y., Tamber H., Planeta M., Leung S.N.S. (2016). Thermoforming of Polylactic Acid Foam Sheets: Crystallization Behaviors and Thermal Stability. Ind. Eng. Chem. Res..

[101] Tabatabaei A., Park C.B. (2017). In-situ visualization of PLA crystallization and crystal effects on foaming in extrusion. Eur. Polym. J..

[102] Wang J., Zhu W., Zhang H., Park C.B. (2012). Continuous processing of low-density, microcellular poly(lactic acid) foams with controlled cell morphology and crystallinity. Chem. Eng. Sci..

[103] Tor-Świątek A., Garbacz T., Sedlarik V., Stloukal P., Kucharczyk P. (2017). Influence of Polylactide Modification with Blowing Agents on Selected Mechanical Properties. Adv. Sci. Technol. Res. J..

[104] Ludwiczak J., Kozlowski M. (2015). Foaming of Polylactide in the Presence of Chain Extender. J. Polym. Environ..

[105] Zhang R., Cai C., Liu Q., Hu S. (2017). Enhancing the Melt Strength of Poly(Lactic Acid) via Micro-Crosslinking and Blending with Poly(Butylene Adipate-co-Butylene Terephthalate)for the Preparation of Foams. J. Polym. Environ..

[106] Ludwiczak J., Kozlowski M. (2015). Dynamic mechanical properties of foamed polylactide and polylactide/wood flour composites. J. Biobased Mater. Bioenergy.

[107] Julien J.M., Quantin J.C., Bénézet J.C., Bergeret A., Lacrampe M.F., Krawczak P. (2015). Chemical foaming extrusion of poly(lactic acid) with chain-extenders: Physical and morphological characterizations. Eur. Polym. J..

[108] Larsen Å., Neldin C. (2013). Physical extruder foaming of poly(lactic acid)-processing and foam properties. Polym. Eng. Sci..

[109] Matuana L.M., Diaz C.a. (2010). Study of Cell Nucleation in Microcellular Poly(lactic acid) Foamed with Supercritical CO_2_ through a Continuous-Extrusion Process. Ind. Eng. Chem. Res..

[110] Matuana L.M. (2008). Solid state microcellular foamed poly(lactic acid): Morphology and property characterization. Bioresour. Technol..

[111] Mihai M., Huneault M.A., Favis B.D. (2010). Rheology and extrusion foaming of chain-branched poly(lactic acid). Polym. Eng. Sci..

[112] Pilla S., Kim S.G., Auer G.K., Gong S., Park C.B. (2010). Microcellular extrusion foaming of poly(lactide)/poly(butylene adipate-co-terephthalate) blends. Mater. Sci. Eng. C.

[113] Lee J.W.S., Lee R.E., Wang J., Jung P.U., Park C.B. (2017). Study of the foaming mechanisms associated with gas counter pressure and mold opening using the pressure profiles. Chem. Eng. Sci..

[114] Xie P., Wu G., Cao Z., Han Z., Zhang Y., An Y., Yang W. (2018). Effect of Mold Opening Process on Microporous Structure and Properties of Microcellular Polylactide–Polylactide Nanocomposites. Polymers.

[115] Volpe V., De Filitto M., Klofacova V., De Santis F., Pantani R. (2018). Effect of mold opening on the properties of PLA samples obtained by foam injection molding. Polym. Eng. Sci..

[116] Ameli A., Jahani D., Nofar M., Jung P.U., Park C.B. (2014). Development of high void fraction polylactide composite foams using injection molding: Mechanical and thermal insulation properties. Compos. Sci. Technol..

[117] Ameli A., Nofar M., Jahani D., Rizvi G., Park C.B. (2015). Development of high void fraction polylactide composite foams using injection molding: Crystallization and foaming behaviors. Chem. Eng. J..

[118] Hou J., Zhao G., Wang G., Dong G., Xu J. (2017). A novel gas-assisted microcellular injection molding method for preparing lightweight foams with superior surface appearance and enhanced mechanical performance. Mater. Des..

[119] Seo J.H., Han J., Lee K.S., Cha S.W. (2012). Combined Effects of Chemical and Microcellular Foaming on Foaming Characteristics of PLA (Poly Lactic Acid) in Injection Molding Process. Polym.-Plast. Technol. Eng..

[120] Najafi N., Heuzey M.C., Carreau P.J., Therriault D., Park C.B. (2015). Mechanical and morphological properties of injection molded linear and branched-polylactide (PLA) nanocomposite foams. Eur. Polym. J..

[121] Hwang S.-S., Hsu P.P., Yeh J.-M., Chang K.-C., Lai Y.-Z. (2009). The mechanical/thermal properties of microcellular injection-molded poly-lactic-acid nanocomposites. Polym. Compos..

[122] Kramschuster A., Turng L.S. (2010). An injection molding process for manufacturing highly porous and interconnected biodegradable polymer matrices for use as tissue engineering scaffolds. J. Biomed. Mater. Res.-Part B Appl. Biomater..

[123] Ameli A., Jahani D., Nofar M., Jung P.U., Park C.B. (2013). Processing and characterization of solid and foamed injection-molded polylactide with talc. J. Cell. Plast..

[124] Pantani R., Sorrentino A., Volpe V., Titomanlio G. (2014). Foam injection molding of poly(lactic acid) with physical blowing agents. Proceedings of the 29th International Conference of the Polymer Processing Society—Conference Papers.

[125] Volpe V., De Filitto M., Klofacova V., De Santis F., Pantani R. (2017). Effect of processing conditions on the cell morphology distribution in foamed injection molded PLA samples. Proceedings of the 32nd International Conference of the Polymer Processing Society—Conference Papers.

[126] Lee J., Moriyama K., Hikima Y., Ohshima M. (2023). Poly(3-hydroxybutyrate-co-3-hydroxyhexanoate) microcellular foams using a melt memory effect as bubble nucleation sites. J. Appl. Polym. Sci..

[127] Pradeep S.A., Kharbas H., Turng L.-S.S., Avalos A., Lawrence J.G., Pilla S. (2017). Investigation of thermal and thermomechanical properties of biodegradable PLA/PBSA composites processed via supercritical fluid-assisted foam injection molding. Polymers.

[128] Sun X., Kharbas H., Peng J., Turng L.-S. (2015). A novel method of producing lightweight microcellular injection molded parts with improved ductility and toughness. Polymer.

[129] Ding Y.F., Hassan M.H., Bakker O., Hinduja S., Bártolo P. (2021). A Review on Microcellular Injection Moulding. Materials.

[130] Martini-Vvedensky J.E., Suh N.P., Church F., Waldman F.A. (1984). Microcellular Closed Cell Foams and Their Method of Manufacture. U.S. Patent.

[131] Pantani R., Volpe V., Titomanlio G. (2014). Foam injection molding of poly(lactic acid) with environmentally friendly physical blowing agents. J. Mater. Process. Technol..

[132] Kramschuster A., Gong S., Turng L.-S., Li T., Li T. (2007). Injection-Molded Solid and Microcellular Polylactide and Polylactide Nanocomposites. J. Biobased Mater. Bioenergy.

[133] Zhao H., Zhao G. (2016). Mechanical and thermal properties of conventional and microcellular injection molded poly (lactic acid)/poly (ε-caprolactone) blends. J. Mech. Behav. Biomed. Mater..

[134] Sun H., Sur G.S., Mark J.E. (2002). Microcellular foams from polyethersulfone and polyphenylsulfone: Preparation and mechanical properties. Eur. Polym. J..

[135] Goswami J., Bhatnagar N., Mohanty S., Ghosh A.K. (2013). Processing and characterization of poly(lactic acid) based bioactive composites for biomedical scaffold application. Express Polym. Lett..

[136] Ludwiczak J., Frackowiak S., Łuzny R. (2018). Effect of recycling on the cellular structure of polylactide in a batch process. Cell. Polym..

[137] Shi X., Wang L., Kang Y., Qin J., Li J., Zhang H., Fan X., Liu Y., Zhang G. (2018). Effect of poly(butylenes succinate) on the microcellular foaming of polylactide using supercritical carbon dioxide. J. Polym. Res..

[138] Wang J., Zhai W., Ling J., Shen B., Zheng W., Park C.B. (2011). Ultrasonic irradiation enhanced cell nucleation in microcellular poly(lactic acid): A novel approach to reduce cell size distribution and increase foam expansion. Ind. Eng. Chem. Res..

[139] Brütting C., Dreier J., Bonten C., Altstädt V., Ruckdäschel H. (2021). Amorphous polylactide bead foam–effect of talc and chain extension on foaming behavior and compression properties. J. Renew. Mater..

[140] Bao D., Liao X., He T., Yang Q., Li G. (2013). Preparation of nanocellular foams from polycarbonate/poly(lactic acid) blend by using supercritical carbon dioxide. J. Polym. Res..

[141] Zimnyakov D., Zdrajevsky R., Minaev N., Epifanov E., Popov V., Ushakova O. (2020). Extreme Foaming Modes for SCF-Plasticized Polylactides: Quasi-Adiabatic and Quasi-Isothermal Foam Expansion. Polymers.

[142] Dippold M., Ruckdäschel H. (2022). Influence of pressure-induced temperature drop on the foaming behavior of amorphous polylactide (PLA) during autoclave foaming with supercritical CO_2_. J. Supercrit. Fluids.

[143] Standau T., Long H., Murillo Castellón S., Brütting C., Bonten C., Altstädt V. (2020). Evaluation of the Zero Shear Viscosity, the D-Content and Processing Conditions as Foam Relevant Parameters for Autoclave Foaming of Standard Polylactide (PLA). Materials.

[144] Chen J., Yang L., Chen D., Mai Q., Wang M., Wu L., Kong P. (2021). Cell structure and mechanical properties of microcellular PLA foams prepared via autoclave constrained foaming. Cell. Polym..

[145] Athanasoulia I.G., Louli V., Schinas P., Rinotas V., Douni E., Tarantili P., Magoulas K. (2022). The effect of foaming process with supercritical CO_2_ on the morphology and properties of 3D porous polylactic acid scaffolds. Polym. Eng. Sci..

[146] Tammaro D., Loianno V., Errichiello F., Di Maio E. (2022). Matricial foaming. Polym. Test..

[147] Dreier J., Brütting C., Ruckdäschel H., Altstädt V., Bonten C. (2021). Investigation of the Thermal and Hydrolytic Degradation of Polylactide during Autoclave Foaming. Polymers.

[148] Li P., Zhu X., Kong M., Lv Y., Huang Y., Yang Q., Li G. (2021). Fully biodegradable polylactide foams with ultrahigh expansion ratio and heat resistance for green packaging. Int. J. Biol. Macromol..

[149] Banerjee R., Ray S.S. (2020). Foamability and Special Applications of Microcellular Thermoplastic Polymers: A Review on Recent Advances and Future Direction. Macromol. Mater. Eng..

[150] Zhang Y., Lu B., Lv F., Guo W., Ji J., Chu P.K., Zhang C. (2012). Effect of processing conditions on poly(butylene succinate) foam materials. J. Appl. Polym. Sci..

[151] Li G., Qi R., Lu J., Hu X., Luo Y., Jiang P. (2013). Rheological properties and foam preparation of biodegradable poly(butylene succinate). J. Appl. Polym. Sci..

[152] Yue J.-F.F., Gan L., Liu C.-H.H., Ma X.-Z.Z., Wang D., Huang J. (2018). Heat-counteracted strategy for tailoring the cell structure and properties of sustainable poly(butylene succinate) foams. Polymer.

[153] Soykeabkaew N., Thanomsilp C., Suwantong O. (2015). A review: Starch-based composite foams. Compos. Part A Appl. Sci. Manuf..

[154] Bergel B.F., Leite Araujo L., dos Santos da Silva A.L., Campomanes Santana R.M. (2020). Effects of silylated starch structure on hydrophobization and mechanical properties of thermoplastic starch foams made from potato starch. Carbohydr. Polym..

[155] Glenn G.M., Orts W.J., Nobes G.A.R. (2001). Starch, fiber and CaCo3 effects on the physical properties of foams made by a baking process. Ind. Crops Prod..

[156] Vercelheze A.E.S., Fakhouri F.M., Dall’Antônia L.H., Urbano A., Youssef E.Y., Yamashita F., Mali S. (2012). Properties of baked foams based on cassava starch, sugarcane bagasse fibers and montmorillonite. Carbohydr. Polym..

[157] Perez-Puyana V., Jiménez-Rosado M., Romero A., Guerrero A. (2020). Polymer-Based Scaffolds for Soft-Tissue Engineering. Polymers.

[158] Soleymani Eil Bakhtiari S., Karbasi S., Toloue E.B. (2021). Modified poly(3-hydroxybutyrate)-based scaffolds in tissue engineering applications: A review. Int. J. Biol. Macromol..

[159] Guo G., Ma Q., Zhao B., Zhang D. (2013). Ultrasound-assisted permeability improvement and acoustic characterization for solid-state fabricated PLA foams. Ultrason. Sonochem..

[160] Gandhi A., Asija N., Chauhan H., Bhatnagar N. (2014). Ultrasound-induced nucleation in microcellular polymers. J. Appl. Polym. Sci..

[161] Wang X., Li W., Kumar V. (2006). A method for solvent-free fabrication of porous polymer using solid-state foaming and ultrasound for tissue engineering applications. Biomaterials.

[162] Wang X., Li W., Kumar V. (2009). Creating Open-celled Solid-state Foams Using Ultrasound. J. Cell. Plast..

[163] Lopez-Gil A., Silva-Bellucci F., Velasco D., Ardanuy M., Rodriguez-Perez M.A.A. (2015). Cellular structure and mechanical properties of starch-based foamed blocks reinforced with natural fibers and produced by microwave heating. Ind. Crops Prod..

[164] Zhao J., Zheng S., Xu Z.-b., Huang X.J.J.o.M.S. (2021). Microwave foaming of polymers. J. Mater. Sci..

[165] Srinivas Sundarram S., Ibekwe N., Prado S., Rotonto C., Feeney S.J.P.E. (2022). Science. Microwave foaming of carbon dioxide saturated poly lactic acid. Polym. Eng. Sci..

[166] Ligon S.C., Liska R., Stampfl J., Gurr M., Mülhaupt R. (2017). Polymers for 3D Printing and Customized Additive Manufacturing. Chem. Rev..

[167] Gibson I., Rosen D.W., Stucker B. (2010). Additive Manufacturing Technologies.

[168] Cano-Vicent A., Tambuwala M.M., Hassan S.S., Barh D., Aljabali A.A.A., Birkett M., Arjunan A., Serrano-Aroca Á. (2021). Fused deposition modelling: Current status, methodology, applications and future prospects. Addit. Manuf..

[169] Pérez M., Medina-Sánchez G., García-Collado A., Gupta M., Carou D. (2018). Surface Quality Enhancement of Fused Deposition Modeling (FDM) Printed Samples Based on the Selection of Critical Printing Parameters. Materials.

[170] Zhou C., Yang K., Wang K., Pei X., Dong Z., Hong Y., Zhang X. (2016). Combination of fused deposition modeling and gas foaming technique to fabricated hierarchical macro/microporous polymer scaffolds. Mater. Des..

[171] Alduais A., Özerinç S. (2023). Tunable mechanical properties of thermoplastic foams produced by additive manufacturing. Express Polym. Lett..

[172] de Freitas F., Pegado H. (2023). Impact of nozzle temperature on dimensional and mechanical characteristics of low-density PLA. Int. J. Adv. Manuf. Technol..

[173] Sarikhani K., Jeddi K., Thompson R.B., Park C.B., Chen P. (2015). Effect of pressure and temperature on interfacial tension of poly lactic acid melt in supercritical carbon dioxide. Thermochim. Acta.

[174] Mahmood S.H., Ameli A., Hossieny N., Park C.B. (2014). The interfacial tension of molten polylactide in supercritical carbon dioxide. J. Chem. Thermodyn..

[175] Nofar M., Salehiyan R., Sinha Ray S. (2019). Rheology of poly (lactic acid)-based systems. Polym. Rev..

[176] Marascio M.G.M., Antons J., Pioletti D.P., Bourban P.-E. (2017). 3D Printing of Polymers with Hierarchical Continuous Porosity. Adv. Mater. Technol..

[177] Choi W.J., Hwang K.S., Kwon H.J., Lee C., Kim C.H., Kim T.H., Heo S.W., Kim J.-H., Lee J.-Y. (2020). Rapid development of dual porous poly(lactic acid) foam using fused deposition modeling (FDM) 3D printing for medical scaffold application. Mater. Sci. Eng. C.

[178] Damanpack A.R., Sousa A., Bodaghi M. (2021). Porous PLAs with Controllable Density by FDM 3D Printing and Chemical Foaming Agent. Micromachines.

[179] Hu B., Li M., Jiang J., Zhai W. (2021). Development of microcellular thermoplastic polyurethane honeycombs with tailored elasticity and energy absorption via CO_2_ foaming. Int. J. Mech. Sci..

[180] Kakumanu V., Srinivas Sundarram S. (2018). Dual pore network polymer foams for biomedical applications via combined solid state foaming and additive manufacturing. Mater. Lett..

[181] Azdast T., Hasanzadeh R. (2021). Polylactide scaffold fabrication using a novel combination technique of fused deposition modeling and batch foaming: Dimensional accuracy and structural properties. Int. J. Adv. Manuf. Technol..

[182] Park B., Hwang D., Kwon D., Yoon T., Lee Y.-W. (2018). Fabrication and Characterization of Multiscale PLA Structures Using Integrated Rapid Prototyping and Gas Foaming Technologies. Nanomaterials.

[183] Song P., Zhou C., Fan H., Zhang B., Pei X., Fan Y., Jiang Q., Bao R., Yang Q., Dong Z. (2018). Novel 3D porous biocomposite scaffolds fabricated by fused deposition modeling and gas foaming combined technology. Compos. Part B Eng..

[184] Loffredo F., Villani F., Choy Buentello D., Trujillo-de Santiago G., Alvarez M.M., Miscioscia R., Di Maio E. (2022). Bubble-Patterned Films by Inkjet Printing and Gas Foaming. Coatings.

[185] Sanz-Horta R., Elvira C., Gallardo A., Reinecke H., Rodríguez-Hernández J. (2020). Fabrication of 3D-Printed Biodegradable Porous Scaffolds Combining Multi-Material Fused Deposition Modeling and Supercritical CO_2_ Techniques. Nanomaterials.

[186] Backes E.H., Fernandes E.M., Diogo G.S., Marques C.F., Silva T.H., Costa L.C., Passador F.R., Reis R.L., Pessan L.A. (2021). Engineering 3D printed bioactive composite scaffolds based on the combination of aliphatic polyester and calcium phosphates for bone tissue regeneration. Mater. Sci. Eng. C.

[187] Ribeiro J.F.M., Oliveira S.M., Alves J.L., Pedro A.J., Reis R.L., Fernandes E.M., Mano J.F. (2017). Structural monitoring and modeling of the mechanical deformation of three-dimensional printed poly(ε-caprolactone) scaffolds. Biofabrication.

[188] Lepcio P., Svatík J., Režnáková E., Zicha D., Lesser A.J., Ondreáš F. (2022). Anisotropic solid-state PLA foaming templated by crystal phase pre-oriented with 3D printing: Cell supporting structures with directional capillary transfer function. J. Mater. Chem. B.

[189] Simpson A., Rattigan I.G., Kalavsky E., Parr G. (2020). Thermal conductivity and conditioning of grey expanded polystyrene foams. Cell. Polym..

[190] Huang P., Su Y., Luo H., Lan X., Chong Y., Wu F., Zheng W. (2022). Facile one-step method to manufacture polypropylene bead foams with outstanding thermal insulation and mechanical properties via supercritical CO_2_ extrusion foaming. J. CO_2_ Util..

[191] Parker K., Garancher J.P., Shah S., Fernyhough A. (2011). Expanded polylactic acid—An eco-friendly alternative to polystyrene foam. J. Cell. Plast..

[192] Doroudiani S., Park C.B., Kortschot M.T. (1996). Effect of the crystallinity and morphology on the microcellular foam structure of semicrystalline polymers. Polym. Eng. Sci..

[193] Kenji H., Hajime O. (2011). Expandable Polylactic Acid Resin Particles, Expanded Polylactic Acid Resin Beads and Molded Article Obtained from Expanded Polylactic Acid Resin Beads. U.S. Patent.

[194] Britton R.N., Van Doormalen F.A.H.C., Noordegraaf J., Molenveld K., Schennink G.G.J. (2012). Coated Particulate Expandable Polylactic Acid. U.S. Patent.

[195] Lohmann J., Sampath B.D.S., Gutmann P., Künkel A., Hahn K., Füßl A. (2013). Process for Producing Expandable Pelletized Material Which Comprises Polylactic Acid. U.S. Patent.

[196] Rossacci J., Shivkumar S. (2003). Bead fusion in polystyrene foams. J. Mater. Sci..

[197] Höhne C.C., Schmidt R., Berner V., Metzsch-Zilligen E., Westphal E., Pfaendner R., Mack C. (2021). Intrinsic flame retardancy of poly(lactic acid) bead foams. J. Appl. Polym. Sci..

[198] Nofar M., Ameli A., Park C.B. (2015). Development of polylactide bead foams with double crystal melting peaks. Polymer.

[199] Nofar M., Ameli A., Park C.B. (2015). A novel technology to manufacture biodegradable polylactide bead foam products. Mater. Des..

[200] Nofar M., Tabatabaei A., Sojoudiasli H., Park C.B., Carreau P.J., Heuzey M.C., Kamal M.R. (2017). Mechanical and bead foaming behavior of PLA-PBAT and PLA-PBSA blends with different morphologies. Eur. Polym. J..

[201] Xu D., Liu P., Wang Q. (2021). An ultrafast and clean method to manufacture poly(vinyl alcohol) bead foam products. Polym. Adv. Technol..

[202] Gumede T.P., Luyt A.S., Müller A.J. (2018). Review on PCL, PBS, AND PCL/PBS blends containing carbon nanotubes. Express Polym. Lett..

[203] Gurunathan T., Mohanty S., Nayak S.K. (2015). A review of the recent developments in biocomposites based on natural fibres and their application perspectives. Compos. Part A Appl. Sci. Manuf..

[204] Rajeshkumar G., Arvindh Seshadri S., Devnani G.L., Sanjay M.R., Siengchin S., Prakash Maran J., Al-Dhabi N.A., Karuppiah P., Mariadhas V.A., Sivarajasekar N. (2021). Environment friendly, renewable and sustainable poly lactic acid (PLA) based natural fiber reinforced composites—A comprehensive review. J. Clean. Prod..

[205] Nakajima H., Dijkstra P., Loos K. (2017). The Recent Developments in Biobased Polymers toward General and Engineering Applications: Polymers that are Upgraded from Biodegradable Polymers, Analogous to Petroleum-Derived Polymers, and Newly Developed. Polymers.

[206] Yan Z., Liao X., He G., Li S., Guo F., Li G. (2019). Green Method to Widen the Foaming Processing Window of PLA by Introducing Stereocomplex Crystallites. Ind. Eng. Chem. Res..

[207] Li B., Zhao G., Wang G., Zhang L., Hou J., Gong J. (2019). A green strategy to regulate cellular structure and crystallization of poly(lactic acid) foams based on pre-isothermal cold crystallization and CO_2_ foaming. Int. J. Biol. Macromol..

[208] Wang J., Chai J., Wang G., Zhao J., Zhang D., Li B., Zhao H., Zhao G. (2019). Strong and thermally insulating polylactic acid/glass fiber composite foam fabricated by supercritical carbon dioxide foaming. Int. J. Biol. Macromol..

[209] Yang Y., Li X., Zhang Q., Xia C., Chen C., Chen X., Yu P. (2019). Foaming of poly(lactic acid) with supercritical CO_2_: The combined effect of crystallinity and crystalline morphology on cellular structure. J. Supercrit. Fluids.

[210] Kuska R., Milovanovic S., Frerich S., Ivanovic J. (2019). Thermal analysis of polylactic acid under high CO_2_ pressure applied in supercritical impregnation and foaming process design. J. Supercrit. Fluids.

[211] Milovanovic S., Markovic D., Mrakovic A., Kuska R., Zizovic I., Frerich S., Ivanovic J. (2019). Supercritical CO_2_-assisted production of PLA and PLGA foams for controlled thymol release. Mater. Sci. Eng. C.

[212] Yin D., Xiang A., Li Y., Qi H., Tian H., Fan G. (2019). Effect of Plasticizer on the Morphology and Foaming Properties of Poly(vinyl alcohol) Foams by Supercritical CO_2_ Foaming Agents. J. Polym. Environ..

[213] Liu P., Chen W., Liu C., Tian M., Liu P. (2019). A novel poly (vinyl alcohol)/poly (ethylene glycol) scaffold for tissue engineering with a unique bimodal open-celled structure fabricated using supercritical fluid foaming. Sci. Rep..

[214] Manavitehrani I., Le T.Y.L., Daly S., Wang Y., Maitz P.K., Schindeler A., Dehghani F. (2019). Formation of porous biodegradable scaffolds based on poly(propylene carbonate) using gas foaming technology. Mater. Sci. Eng. C.

[215] Liu Z., Hu J., Gao F., Cao H., Zhou Q., Wang X. (2019). Biodegradable and resilient poly (propylene carbonate)based foam from high pressure CO_2_ foaming. Polym. Degrad. Stab..

[216] Zhou H., Song J., Ding X., Qu Z., Wang X., Mi J., Wang J. (2019). Cellular morphology evolution of chain extended poly(butylene succinate)/organic montmorillonite nanocomposite foam. J. Appl. Polym. Sci..

[217] Chen Z., Hu J., Ju J., Kuang T. (2019). Fabrication of Poly(butylene succinate)/Carbon Black Nanocomposite Foams with Good Electrical Conductivity and High Strength by a Supercritical CO_2_ Foaming Process. Polymers.

[218] García-Casas I., Montes A., Valor D., Pereyra C., Martínez de la Ossa E.J. (2019). Foaming of Polycaprolactone and Its Impregnation with Quercetin Using Supercritical CO_2_. Polymers.

[219] Chen C.X., Peng H.H., Guan Y.X., Yao S.J. (2019). Morphological study on the pore growth profile of poly(ε-caprolactone) bi-modal porous foams using a modified supercritical CO_2_ foaming process. J. Supercrit. Fluids.

[220] Salerno A., Domingo C. (2019). Polycaprolactone foams prepared by supercritical CO_2_ batch foaming of polymer/organic solvent solutions. J. Supercrit. Fluids.

[221] Song C., Li S., Zhang J., Xi Z., Lu E., Zhao L., Cen L. (2019). Controllable fabrication of porous PLGA/PCL bilayer membrane for GTR using supercritical carbon dioxide foaming. Appl. Surf. Sci..

[222] Zhang K., Wang Y., Jiang J., Wang X., Hou J., Sun S., Li Q. (2019). Fabrication of highly interconnected porous poly(ɛ-caprolactone) scaffolds with supercritical CO_2_ foaming and polymer leaching. J. Mater. Sci..

[223] Wang Z., Zhao J., Wang G., Xu Z., Zhang A., Dong G., Zhao G. (2022). Lightweight, low-shrinkage and high elastic poly(butylene adipate-co-terephthalate) foams achieved by microcellular foaming using N_2_ & CO_2_ as co-blowing agents. J. CO_2_ Util..

[224] Wu R., Wang S., Leng Y., Li Q. (2020). Preparation, structure, and properties of poly(ethyleneoxide)/lignin composites used for UV absorption. J. Appl. Polym. Sci..

[225] Ventura H., Laguna-Gutiérrez E., Rodriguez-Perez M.A., Ardanuy M. (2016). Effect of chain extender and water-quenching on the properties of poly(3-hydroxybutyrate-co-4-hydroxybutyrate) foams for its production by extrusion foaming. Eur. Polym. J..

[226] Panaitescu D.M., Trusca R., Gabor A.R., Nicolae C.A., Casarica A. (2020). Biocomposite foams based on polyhydroxyalkanoate and nanocellulose: Morphological and thermo-mechanical characterization. Int. J. Biol. Macromol..

[227] Asikainen S., Paakinaho K., Kyhkynen A.K., Hannula M., Malin M., Ahola N., Kellomäki M., Seppälä J. (2019). Hydrolysis and drug release from poly(ethylene glycol)-modified lactone polymers with open porosity. Eur. Polym. J..

[228] Van Wouwe P., Dusselier M., Vanleeuw E., Sels B. (2016). Lactide Synthesis and Chirality Control for Polylactic acid Production. ChemSusChem.

[229] Lim L.T., Auras R., Rubino M. (2008). Processing technologies for poly(lactic acid). Prog. Polym. Sci..

[230] Auras R., Harte B., Selke S. (2004). An overview of polylactides as packaging materials. Macromol. Biosci..

[231] Nofar M., Sacligil D., Carreau P.J., Kamal M.R., Heuzey M.-C. (2019). Poly (lactic acid) blends: Processing, properties and applications. Int. J. Biol. Macromol..

[232] Litauszki K., Gere D., Czigany T., Kmetty Á. (2023). Environmentally friendly packaging foams: Investigation of the compostability of poly(lactic acid)-based syntactic foams. Sustain. Mater. Technol..

[233] Wang K., Wang J., Zhao D., Zhai W. (2017). Preparation of microcellular poly(lactic acid) composites foams with improved flame retardancy. Cell. Plast..

[234] Wang J., Ren Q., Zheng W., Zhai W. (2014). Improved flame-retardant properties of poly(lactic acid) foams using starch as a natural charring agent. Ind. Eng. Chem. Res..

[235] Suparanon T., Phetwarotai W. (2020). Fire-extinguishing characteristics and flame retardant mechanism of polylactide foams: Influence of tricresyl phosphate combined with natural flame retardant. Int. J. Biol. Macromol..

[236] Jia L., Huang W., Zhao Y., Wen S., Yu Z., Zhang Z. (2022). Ultra-light polylactic acid/combination composite foam: A fully biodegradable flame retardant material. Int. J. Biol. Macromol..

[237] Milovanovic S., Lukic I., Horvat G., Novak Z., Frerich S., Petermann M., García-González C.A. (2023). Green Processing of Neat Poly(lactic acid) Using Carbon Dioxide under Elevated Pressure for Preparation of Advanced Materials: A Review (2012–2022). Polymers.

[238] Garancher J.P.J.P., Fernyhough A. (2014). Expansion and dimensional stability of semi-crystalline polylactic acid foams. Polym. Degrad. Stab..

[239] Li B., Ma X., Zhao G., Wang G., Zhang L., Gong J. (2020). Green fabrication method of layered and open-cell polylactide foams for oil-sorption via pre-crystallization and supercritical CO_2_-induced melting. J. Supercrit. Fluids.

[240] Peng Q., Li S., Liu F., Liao X., Li G. (2023). Effect of CO_2_ on the crystallization of poly(lactic acid) homo-crystallites via influencing the crystal structure of stereocomplex crystallites. CrystEngComm.

[241] Vatansever E., Arslan D., Nofar M. (2019). Polylactide cellulose-based nanocomposites. Int. J. Biol. Macromol..

[242] Wu Y., Zhang S., Han S., Yu K., Wang L. (2022). Regulating cell morphology of poly (lactic acid) foams from microcellular to nanocellular by crystal nucleating agent. Polym. Degrad. Stab..

[243] Li S., He G., Liao X., Park C.B., Yang Q., Li G. (2017). Introduction of a long-chain branching structure by ultraviolet-induced reactive extrusion to improve cell morphology and processing properties of polylactide foam. RSC Adv..

[244] Zhou M., Zhou P., Xiong P., Qian X., Zheng H. (2015). Crystallization, rheology and foam morphology of branched PLA prepared by novel type of chain extender. Macromol. Res..

[245] Wang X., Zhou H., Liu B., Du Z., Li H. (2014). Chain Extension and Foaming Behavior of Poly(lactic acid) by Functionalized Multiwalled Carbon Nanotubes and Chain Extender. Adv. Polym. Technol..

[246] Ni J., Yu K., Zhou H., Mi J., Chen S., Wang X. (2020). Morphological evolution of PLA foam from microcellular to nanocellular induced by cold crystallization assisted by supercritical CO_2_. J. Supercrit. Fluids.

[247] Narmon A.S., Dewaele A., Bruyninckx K., Sels B.F., Van Puyvelde P., Dusselier M. (2021). Boosting PLA melt strength by controlling the chirality of co-monomer incorporation. Chem. Sci..

[248] Chen J., Yang L., Mai Q., Li M., Wu L., Kong P. (2021). Foaming behavior of poly(lactic acid) with different D-isomer content based on supercritical CO_2_-induced crystallization. J. Cell. Plast..

[249] Yu K., Wu Y., Zhang X., Hou J., Chen J. (2022). Microcellular open-cell poly(l-lactic acid)/poly(d-lactic acid) foams for oil-water separation prepared via supercritical CO_2_ foaming. J. CO_2_ Util..

[250] Zhao H., Yan X., Zhao G., Guo Z. (2016). Microcellular injection molded polylactic acid/poly (ε-caprolactone) blends with supercritical CO_2_: Correlation between rheological properties and their foaming behavior. Polym. Eng. Sci..

[251] Zhao H., Cui Z., Sun X., Turng L.S., Peng X. (2013). Morphology and properties of injection molded solid and microcellular polylactic acid/polyhydroxybutyrate-valerate (PLA/PHBV) blends. Ind. Eng. Chem. Res..

[252] Matuana L.M., Diaz C.A. (2013). Strategy to produce microcellular foamed poly(lactic acid)/wood-flour composites in a continuous extrusion process. Ind. Eng. Chem. Res..

[253] Liu W., Wang X., Li H., Du Z., Zhang C. (2013). Study on rheological and extrusion foaming behaviors of chain-extended poly (lactic acid)/clay nanocomposites. J. Cell. Plast..

[254] Hou Y., Pan Y., Zhou Z., Liu C., Shen C., Liu X. (2023). Review on Cell Structure Regulation and Performances Improvement of Porous Poly(Lactic Acid). Macromol. Rapid Commun..

[255] Xu L.Q., Huang H.X. (2014). Foaming of poly(lactic acid) using supercritical carbon dioxide as foaming agent: Influence of crystallinity and spherulite size on cell structure and expansion ratio. Ind. Eng. Chem. Res..

[256] Zhang X., Ding W., Zhao N., Chen J., Park C.B. (2018). Effects of Compressed CO_2_ and Cotton Fibers on the Crystallization and Foaming Behaviors of Polylactide. Ind. Eng. Chem. Res..

[257] Xue S., Jia P., Ren Q., Liu X., Lee R.E., Zhai W. (2018). Improved expansion ratio and heat resistance of microcellular poly(L-lactide) foam via in-situ formation of stereocomplex crystallites. J. Cell. Plast..

[258] Xiang P., Gou L., Zou Y., Chen B., Bi S., Chen X., Yu P. (2022). A facile strategy for preparation of strong tough poly(lactic acid) foam with a unique microfibrillated bimodal micro/nano cellular structure. Int. J. Biol. Macromol..

[259] Nofar M., Tabatabaei A., Park C.B. (2013). Effects of nano-/micro-sized additives on the crystallization behaviors of PLA and PLA/CO_2_ mixtures. Polymer.

[260] Chen P., Wang W., Wang Y., Yu K., Zhou H., Wang X., Mi J. (2017). Crystallization-induced microcellular foaming of poly (lactic acid) with high volume expansion ratio. Polym. Degrad. Stab..

[261] Zhou G., Liu W., Yin H., Zhang Y., Huang C. (2023). Effect of nano-sized zinc citrate on the supercritical carbon dioxide-assisted extrusion foaming behavior of poly(lactic acid). J. Appl. Polym. Sci..

[262] Li W., Ren Q., Zhu X., Wu M., Weng Z., Wang L., Zheng W. (2022). Enhanced heat resistance and compression strength of microcellular poly (lactic acid) foam by promoted stereocomplex crystallization with added D-Mannitol. J. CO_2_ Util..

[263] Cho S.Y., Park H.H., Yun Y.S., Jin H.J. (2013). Influence of cellulose nanofibers on the morphology and physical properties of poly(lactic acid) foaming by supercritical carbon dioxide. Macromol. Res..

[264] Qiu Y., Lv Q., Wu D., Xie W., Peng S., Lan R., Xie H. (2018). Cyclic tensile properties of the polylactide nanocomposite foams containing cellulose nanocrystals. Cellulose.

[265] Ren Q., Li W., Cui S., Ma W., Zhu X., Wu M., Wang L., Zheng W., Semba T., Ohshima M. (2023). Improved thermal insulation and compressive property of bimodal poly (lactic acid)/cellulose nanocomposite foams. Carbohydr. Polym..

[266] Wang B., Qi Z., Chen X., Sun C., Yao W., Zheng H., Liu M., Li W., Qin A., Tan H. (2022). Preparation and mechanism of lightweight wood fiber/poly(lactic acid) composites. Int. J. Biol. Macromol..

[267] Li J., Wang Y.X., Lin J., Liu Y., Wang G.L., Quan D., Guan Y.J., Zhao G.Q., Ji S.C. (2023). Environmentally-friendly, sustainable ScCO_2_-assisted fabrication of poly (lactic acid)/ramie fiber composite foams. J. Clean. Prod..

[268] Moutinho L.G., Soares E., Oliveira M. (2023). Development of bio-based expanded cork polymer composites (eCPC) with poly(lactic acid) (PLA). Mater. Sci. Eng. B.

[269] Sun J., Zhao Z., Pang Y., Liu J., Zhang W., Wang B., Xu L., Guo H., Liu Y. (2023). The Facile and Efficient Fabrication of Rice Husk/poly (lactic acid) Foam Composites by Coordinated the Interface Combination and Bubble Hole Structure. Int. J. Biol. Macromol..

[270] Haham H., Riscoe A., Frank C.W., Billington S.L. (2021). Effect of bubble nucleating agents derived from biochar on the foaming mechanism of poly lactic acid foams. Appl. Surf. Sci. Adv..

[271] Zhao H., Zhao G., Turng L.S., Peng X. (2015). Enhancing Nanofiller Dispersion Through Prefoaming and Its Effect on the Microstructure of Microcellular Injection Molded Polylactic Acid/Clay Nanocomposites. Ind. Eng. Chem. Res..

[272] Kuang T.R., Mi H.Y., Fu D.J., Jing X., Chen B.Y., Mou W.J., Peng X.F. (2015). Fabrication of poly(lactic acid)/graphene oxide foams with highly oriented and elongated cell structure via unidirectional foaming using supercritical carbon dioxide. Ind. Eng. Chem. Res..

[273] Soleimanpour A., Khonakdar H., Mousavi S.R., Banaei N., Hemmati F., Arjmand M., Ruckdäschel H., Khonakdar H.A. (2023). Continuous extrusion foaming process of biodegradable nanocomposites based on poly(lactic acid)/carbonaceous nanoparticles with different geometric shapes: An insight into involved physical, chemical and rheological phenomena. J. Appl. Polym. Sci..

[274] Tang Y., Wang Y., Chen S., Wang X. (2022). Fabrication of low-density poly(lactic acid) microcellular foam by self-assembly crystallization nucleating agent. Polym. Degrad. Stab..

[275] Mort R., Peters E., Curtzwiler G., Jiang S., Vorst K. (2022). Biofillers Improved Compression Modulus of Extruded PLA Foams. Sustainability.

[276] Abu Hassan N.A., Ahmad S., Chen R.S., Shahdan D. (2020). Cells analyses, mechanical and thermal stability of extruded polylactic acid/kenaf bio-composite foams. Constr. Build. Mater..

[277] Rojas A., Torres A., López de Dicastillo C., Velásquez E., Villegas C., Faba S., Rivera P., Guarda A., Romero J., Galotto M. (2022). Foaming with scCO_2_ and Impregnation with Cinnamaldehyde of PLA Nanocomposites for Food Packaging. Processes.

[278] Wu Y., Yu K., Zhang X., Hou J., Chen J. (2022). Lightweight electromagnetic interference shielding poly(L-lactic acid)/poly(D-lactic acid)/carbon nanotubes composite foams prepared by supercritical CO_2_ foaming. Int. J. Biol. Macromol..

[279] Kuang T., Ju J., Chen F., Liu X., Zhang S., Liu T., Peng X. (2022). Coupled effect of self-assembled nucleating agent, Ni-CNTs and pressure-driven flow on the electrical, electromagnetic interference shielding and thermal conductive properties of poly (lactic acid) composite foams. Compos. Sci. Technol..

[280] Standau T., Goettermann S., Weinmann S., Bonten C., Altstädt V. (2017). Autoclave foaming of chemically modified polylactide. J. Cell. Plast..

[281] Fang F., Niu D., Xu P., Liu T., Yang W., Wang Z., Li X., Ma P. (2023). A Quantitative Study on Branching Density Dependent Behavior of Polylactide Melt Strength. Macromol. Rapid Commun..

[282] Li Y., Zhou H., Wen B., Chen Y., Wang X. (2020). A Facile and Efficient Method for Preparing Chain Extended Poly(lactic acid) Foams with High Volume Expansion Ratio. J. Polym. Environ..

[283] Venkatesan K.B., Karkhanis S.S., Matuana L.M. (2021). Microcellular foaming of poly(lactic acid) branched with food-grade chain extenders. J. Appl. Polym. Sci..

[284] Li P., Zhang W., Zhu X., Kong M., Lv Y., Huang Y., Gong P., Li G. (2020). Simultaneous Improvement of the Foaming Property and Heat Resistance in Polylactide via One-step Branching Reaction Initiated by Cyclic Organic Peroxide. Ind. Eng. Chem. Res..

[285] Li D., Zhang S., Zhao Z., Miao Z., Zhang G., Shi X. (2023). High-Expansion Open-Cell Polylactide Foams Prepared by Microcellular Foaming Based on Stereocomplexation Mechanism with Outstanding Oil–Water Separation. Polymers.

[286] Li P., Zhang W., Kong M., Lv Y., Huang Y., Yang Q., Li G. (2021). Ultrahigh performance polylactide achieved by the design of molecular structure. Mater. Des..

[287] Ren Q., Zhu X., Li W., Wu M., Cui S., Ling Y., Ma X., Wang G., Wang L., Zheng W. (2022). Fabrication of super-hydrophilic and highly open-porous poly (lactic acid) scaffolds using supercritical carbon dioxide foaming. Int. J. Biol. Macromol..

[288] Chen Y., Yang W., Hu Z., Gao X., Ye J., Song X., Chen B., Li Z. (2022). Preparation and properties of oriented microcellular Poly(L-lactic acid) foaming material. Int. J. Biol. Macromol..

[289] Péter T., Litauszki K., Kmetty Á. (2021). Improving the heat deflection temperature of poly(lactic acid) foams by annealing. Polym. Degrad. Stab..

[290] Li S., Chen T., Liao X., Han W., Yan Z., Li J., Li G. (2020). Effect of Macromolecular Chain Movement and the Interchain Interaction on Crystalline Nucleation and Spherulite Growth of Polylactic Acid under High-Pressure CO_2_. Macromolecules.

[291] Volpe V., Pantani R. (2015). Foam injection molding of poly(lactic) acid: Effect of back pressure on morphology and mechanical properties. J. Appl. Polym. Sci..

[292] Chauvet M., Sauceau M., Baillon F., Fages J. (2017). Mastering the structure of PLA foams made with extrusion assisted by supercritical CO_2_. J. Appl. Polym. Sci..

[293] Wu D., Lv Q., Feng S., Chen J., Chen Y., Qiu Y., Yao X. (2015). Polylactide composite foams containing carbon nanotubes and carbon black: Synergistic effect of filler on electrical conductivity. Carbon.

[294] Wang Y., Song Y., Du J., Xi Z., Wang Q. (2017). Preparation of Desirable Porous Cell Structure Polylactide/Wood Flour Composite Foams Assisted by Chain Extender. Materials.

[295] Ding W., Jahani D., Chang E., Alemdar A., Park C.B., Sain M. (2016). Development of PLA/cellulosic fiber composite foams using injection molding: Crystallization and foaming behaviors. Compos. Part A Appl. Sci. Manuf..

[296] Li Y., Yin D., Liu W., Zhou H., Zhang Y., Wang X. (2020). Fabrication of biodegradable poly (lactic acid)/carbon nanotube nanocomposite foams: Significant improvement on rheological property and foamability. Int. J. Biol. Macromol..

[297] Shah Mohammadi M., Rezabeigi E., Bertram J., Marelli B., Gendron R., Nazhat S.N., Bureau M.N. (2020). Poly(d,l-Lactic acid) Composite Foams Containing Phosphate Glass Particles Produced via Solid-State Foaming Using CO_2_ for Bone Tissue Engineering Applications. Polymers.

[298] Sadeghi B., Sadeghi P., Marfavi Y., Kowsari E., Zareiyazd A.A., Ramakrishna S. (2022). Impacts of cellulose nanofibers on the morphological behavior and dynamic mechanical thermal properties of extruded polylactic acid/cellulose nanofibril nanocomposite foam. J. Appl. Polym. Sci..

[299] Jian J., Xiangbin Z., Xianbo H. (2020). An overview on synthesis, properties and applications of poly(butylene-adipate-co-terephthalate)–PBAT. Adv. Ind. Eng. Polym. Res..

[300] Houbben M., Thomassin J.M., Jérôme C. (2022). Supercritical CO_2_ blown poly(ε-caprolactone) covalent adaptable networks towards unprecedented low density shape memory foams. Mater. Adv..

[301] Huang A., Jiang Y., Napiwocki B., Mi H., Peng X., Turng L.S. (2017). Fabrication of poly(ε-caprolactone) tissue engineering scaffolds with fibrillated and interconnected pores utilizing microcellular injection molding and polymer leaching. RSC Adv..

[302] Hou J., Jiang J., Guo H., Guo X., Wang X., Shen Y., Li Q. (2020). Fabrication of fibrillated and interconnected porous poly(ε-caprolactone) vascular tissue engineering scaffolds by microcellular foaming and polymer leaching. RSC Adv..

[303] Li J., Wang H., Zhou H., Jiang J., Wang X., Li Q. (2021). Fabrication of Highly Interconnected Poly(ε-caprolactone)/cellulose Nanofiber Composite Foams by Microcellular Foaming and Leaching Processes. ACS Omega.

[304] Goimil L., Jaeger P., Ardao I., Gómez-Amoza J.L., Concheiro A., Alvarez-Lorenzo C., García-González C.A. (2018). Preparation and stability of dexamethasone-loaded polymeric scaffolds for bone regeneration processed by compressed CO_2_ foaming. J. CO_2_ Util..

[305] Wang L., Zhou H., Wang X., Mi J. (2015). Mechanism of bubble nucleation in poly(ε-caprolactone) foaming at low temperature. Polymer.

[306] Zhong X., Dehghani F. (2012). Fabrication of biomimetic poly(propylene carbonate) scaffolds by using carbon dioxide as a solvent, monomer and foaming agent. Green Chem..

[307] Hatami T., Johner J.C.F., de Castro K.C., Mei L.H.I., Vieira M.G.A., Angela M. (2020). New insight into a step-by-step modeling of supercritical CO_2_ foaming to fabricate poly(ε-caprolactone) scaffold. Ind. Eng. Chem. Res..

[308] Salerno A., Diéguez S., Diaz-Gomez L., Gómez-Amoza J.L., Magariños B., Concheiro A., Domingo C., Alvarez-Lorenzo C., García-González C.A. (2017). Synthetic scaffolds with full pore interconnectivity for bone regeneration prepared by supercritical foaming using advanced biofunctional plasticizers. Biofabrication.

[309] Song C., Luo Y., Liu Y., Li S., Xi Z., Zhao L., Cen L., Lu E. (2020). Fabrication of PCL Scaffolds by Supercritical CO_2_ Foaming Based on the Combined Effects of Rheological and Crystallization Properties. Polymers.

[310] Castano M., Martinez-Campos E., Pintado-Sierra M., García C., Reinecke H., Gallardo A., Rodriguez-Hernandez J., Elvira C. (2018). Combining Breath Figures and Supercritical Fluids to Obtain Porous Polymer Scaffolds. ACS Omega.

[311] Campardelli R., Franco P., Reverchon E., De Marco I. (2019). Polycaprolactone/nimesulide patches obtained by a one-step supercritical foaming + impregnation process. J. Supercrit. Fluids.

[312] Kravanja G., Primožič M., Knez Ž., Leitgeb M. (2020). Transglutaminase release and activity from novel poly(ε-caprolactone)-based composites prepared by foaming with supercritical CO_2_. J. Supercrit. Fluids.

[313] Franco P., Belvedere R., Pessolano E., Liparoti S., Pantani R., Petrella A., De Marco I. (2019). PCL/Mesoglycan Devices Obtained by Supercritical Foaming and Impregnation. Pharmaceutics.

[314] Ivanovic J., Knauer S., Fanovich A., Milovanovic S., Stamenic M., Jaeger P., Zizovic I., Eggers R. (2016). Supercritical CO_2_ sorption kinetics and thymol impregnation of PCL and PCL-HA. J. Supercrit. Fluids.

[315] Salerno A., Saurina J., Domingo C. (2015). Supercritical CO_2_ foamed polycaprolactone scaffolds for controlled delivery of 5-fluorouracil, nicotinamide and triflusal. Int. J. Pharm..

[316] De Matos M.B.C., Puga A.M., Alvarez-Lorenzo C., Concheiro A., Braga M.E.M., De Sousa H.C. (2015). Osteogenic poly(ε-caprolactone)/poloxamine homogeneous blends prepared by supercritical foaming. Int. J. Pharm..

[317] Fanovich M.A., Ivanovic J., Misic D., Alvarez M.V., Jaeger P., Zizovic I., Eggers R. (2013). Development of polycaprolactone scaffold with antibacterial activity by an integrated supercritical extraction and impregnation process. J. Supercrit. Fluids.

[318] Burin G.R.M., Formiga F.R., Pires V.C., Miranda J.C., Barral A., Cabral-Albuquerque E.C.M., Vieira de Melo S.A.B., Braga M.E.M., de Sousa H.C. (2022). Innovative formulations of PCL: Pluronic monoliths with copaiba oleoresin using supercritical CO_2_ foaming/mixing to control Aedes aegypti. J. Supercrit. Fluids.

[319] Fanovich M.A., Ivanovic J., Zizovic I., Misic D., Jaeger P. (2016). Functionalization of polycaprolactone/hydroxyapatite scaffolds with Usnea lethariiformis extract by using supercritical CO_2_. Mater. Sci. Eng. C.

[320] Diaz-Gomez L., García-González C.A., Wang J., Yang F., Aznar-Cervantes S., Cenis J.L., Reyes R., Delgado A., Évora C., Concheiro A. (2017). Biodegradable PCL/fibroin/hydroxyapatite porous scaffolds prepared by supercritical foaming for bone regeneration. Int. J. Pharm..

[321] Chen C.-X., Liu Q.-Q., Xin X., Guan Y.-X., Yao S.-J. (2016). Pore formation of poly(ε-caprolactone) scaffolds with melting point reduction in supercritical CO_2_ foaming. J. Supercrit. Fluids.

[322] Moghadam M.Z., Hassanajili S., Esmaeilzadeh F., Ayatollahi M., Ahmadi M. (2017). Formation of porous HPCL/LPCL/HA scaffolds with supercritical CO_2_ gas foaming method. J. Mech. Behav. Biomed. Mater..

[323] Jing X., Mi H.Y., Turng L.S. (2017). Comparison between PCL/hydroxyapatite (HA) and PCL/halloysite nanotube (HNT) composite scaffolds prepared by co-extrusion and gas foaming. Mater. Sci. Eng. C.

[324] Wang X., Salick M.R., Gao Y., Jiang J., Li X., Liu F., Cordie T., Li Q., Turng L.S. (2018). Interconnected porous poly(ɛ-caprolactone) tissue engineering scaffolds fabricated by microcellular injection molding. J. Cell. Plast..

[325] Moeini A., Germann N., Malinconico M., Santagata G. (2021). Formulation of secondary compounds as additives of biopolymer-based food packaging: A review. Trends Food Sci. Technol..

[326] Zhou H., Hu D., Zhu M., Xue K., Wei X., Park C.B., Wang X., Zhao L. (2023). Review on poly (butylene succinate) foams: Modifications, foaming behaviors and applications. Sustain. Mater. Technol..

[327] Wang X., Zhou J., Li L. (2007). Multiple melting behavior of poly(butylene succinate). Eur. Polym. J..

[328] Sarver J.A., Kiran E. (2021). Foaming of polymers with carbon dioxide—The year-in-review—2019. J. Supercrit. Fluids.

[329] Feng Z., Luo Y., Hong Y., Wu J., Zhu J., Li H., Qi R., Jiang P. (2016). Preparation of Enhanced Poly(butylene succinate) Foams. Polym. Eng. Sci..

[330] Ru K., Zhang S., Peng X., Wang J., Peng H. (2019). Fabrication of Poly(butylene succinate) phosphorus-containing ionomers microcellular foams with significantly improved thermal conductivity and compressive strength. Polymer.

[331] Zhang S., Xu Y., Wang P., Peng X., Zeng J. (2018). Fabrication-controlled morphology of poly(butylene succinate) nano-microcellular foams by supercritical CO_2_. Polym. Adv. Technol..

[332] Zhou X.M., Liu Y.F. (2022). Preparation and properties of high melt strength PBS and its environmentally friendly foaming materials. Cell. Polym..

[333] Ykhlef N., Lafranche E. (2019). Development of bio-based poly(butylene succinate) formulations for microcellular injection foaming. Int. J. Mater. Form..

[334] Zhou H., Wang X., Du Z., Li H., Yu K. (2015). Preparation and characterization of chain extended Poly(butylene succinate) foams. Polym. Eng. Sci..

[335] Chen P., Zhao L., Gao X., Xu Z., Liu Z., Hu D. (2021). Engineering of polybutylene succinate with long-chain branching toward high foamability and degradation. Polym. Degrad. Stab..

[336] Yin D., Mi J., Zhou H., Wang X., Yu K. (2020). Simple and feasible strategy to fabricate microcellular poly(butylene succinate) foams by chain extension and isothermal crystallization induction. J. Appl. Polym. Sci..

[337] Wu W., Cao X., Lin H., He G., Wang M. (2015). Preparation of biodegradable poly(butylene succinate)/halloysite nanotube nanocomposite foams using supercritical CO_2_ as blowing agent. J. Polym. Res..

[338] Huang A., Song X., Liu F., Wang H., Geng L., Wang H., Yi Q., Peng X. (2022). Supercritical Fluids-Assisted Processing Using CO_2_ Foaming to Enhance the Dispersion of Nanofillers in Poly(butylene succinate)-Based Nanocomposites and the Conductivity. J. Polym. Environ..

[339] Kuang T., Ju J., Yang Z., Geng L., Peng X. (2018). A facile approach towards fabrication of lightweight biodegradable poly (butylene succinate)/carbon fiber composite foams with high electrical conductivity and strength. Compos. Sci. Technol..

[340] Fu H., Yin D., Wang T., Gong W., Zhou H. (2022). Open Pore Morphology Evolution in Poly(butylene succinate)/Chitin Nanocrystal Nanocomposite Foams. J. Polym. Environ..

[341] Oliviero M., Sorrentino L., Cafiero L., Galzerano B., Sorrentino A., Iannace S. (2015). Foaming behavior of bio-based blends based on thermoplastic gelatin and poly(butylene succinate). J. Appl. Polym. Sci..

[342] Huang A., Lin J., Tian G., Tan B., Wu F., Geng L., Peng X., Fang H. (2022). Facial Preparation of Segregated Poly(butylene succinate)/Carbon Nanotubes Composite Foams with Superior Conductive Properties via Synergistic Effect of High Pressure Solid Phase Molding and Supercritical Fluid Foaming. Macromol. Mater. Eng..

[343] Thomas D., Cebe P. (2017). Self-nucleation and crystallization of polyvinyl alcohol. J. Therm. Anal. Calorim..

[344] Li Y., Tian H., Jia Q., Niu P., Xiang A., Liu D., Qin Y. (2015). Development of polyvinyl alcohol/intercalated MMT composite foams fabricated by melt extrusion. J. Appl. Polym. Sci..

[345] Xiang A., Yin D., He Y., Li Y., Tian H. (2021). Multifunctional nucleating agents with simultaneous plasticizing, solubilizing, nucleating and their effect on polyvinyl alcohol foams. J. Supercrit. Fluids.

[346] Song T., Tanpichai S., Oksman K. (2016). Cross-linked polyvinyl alcohol (PVA) foams reinforced with cellulose nanocrystals (CNCs). Cellulose.

[347] Azimi H., Jahani D., Aghamohammadi S., Nofar M. (2022). Experimental and numerical investigation of bubble nucleation and growth in supercritical CO_2_-blown poly(vinyl alcohol). Korean J. Chem. Eng..

[348] Popa M.S., Frone A.N., Panaitescu D.M. (2022). Polyhydroxybutyrate blends: A solution for biodegradable packaging?. Int. J. Biol. Macromol..

[349] Horue M., Rivero Berti I., Cacicedo M.L., Castro G.R. (2021). Microbial production and recovery of hybrid biopolymers from wastes for industrial applications- a review. Bioresour. Technol..

[350] Tebaldi M.L., Maia A.L.C., Poletto F., de Andrade F.V., Soares D.C.F. (2019). Poly(-3-hydroxybutyrate-co-3-hydroxyvalerate) (PHBV): Current advances in synthesis methodologies, antitumor applications and biocompatibility. J. Drug Deliv. Sci. Technol..

[351] Bossu J., Le Moigne N., Dieudonné-George P., Dumazert L., Guillard V., Angellier-Coussy H. (2021). Impact of the processing temperature on the crystallization behavior and mechanical properties of poly[R-3-hydroxybutyrate-co-(R-3-hydroxyvalerate)]. Polymer.

[352] Oluwabunmi K.E., Zhao W., D’Souza N.A. (2021). Carbon Capture Utilization for Biopolymer Foam Manufacture: Thermal, Mechanical and Acoustic Performance of PCL/PHBV CO_2_ Foams. Polymers.

[353] Wright Z.C., Frank C.W. (2014). Increasing cell homogeneity of semicrystalline, biodegradable polymer foams with a narrow processing window via rapid quenching. Polym. Eng. Sci..

[354] Szegda D., Duangphet S., Song J., Tarverdi K. (2014). Extrusion foaming of PHBV. J. Cell. Plast..

[355] Xu J.K., Zhang L., Li D.L., Bao J.B., Wang Z.B. (2020). Foaming of Poly(3-hydroxybutyrate- co-3-hydroxyvalerate) with Supercritical Carbon Dioxide: Foaming Performance and Crystallization Behavior. ACS Omega.

[356] Oprică M.G., Uşurelu C.D., Frone A.N., Gabor A.R., Nicolae C.-A., Vasile V., Panaitescu D.M. (2022). Opposite Roles of Bacterial Cellulose Nanofibers and Foaming Agent in Polyhydroxyalkanoate-Based Materials. Polymers.

[357] Tsui A., Frank C.W. (2014). Impact of Processing Temperature and Composition on Foaming of Biodegradable Poly(hydroxyalkanoate) Blends. Ind. Eng. Chem. Res..

[358] Zhang T., Jang Y., Lee E., Shin S., Kang H.-j. (2022). Supercritical CO_2_ Foaming of Poly(3-hydroxybutyrate-co-4-hydroxybutyrate). Polymers.

[359] Luo J., Zhu M., Wang L., Zhou H., Wen B., Wang X., Zhang Y. (2022). CO_2_-based fabrication of biobased and biodegradable poly (3-hydroxybutyrate-co-3-hydroxyvalerate)/graphene nanoplates nanocomposite foams: Toward EMI shielding application. Polymer.

[360] Le Moigne N., Sauceau M., Benyakhlef M., Jemai R., Benezet J.C., Rodier E., Lopez-Cuesta J.M., Fages J. (2014). Foaming of poly(3-hydroxybutyrate-co-3-hydroxyvalerate)/organo-clays nano-biocomposites by a continuous supercritical CO_2_ assisted extrusion process. Eur. Polym. J..

[361] Srithep Y., Ellingham T., Peng J., Sabo R., Clemons C., Turng L.S., Pilla S. (2013). Melt compounding of poly (3-hydroxybutyrate-co-3-hydroxyvalerate)/ nanofibrillated cellulose nanocomposites. Polym. Degrad. Stab..

[362] Dedieu I., Peyron S., Gontard N., Aouf C. (2022). The thermo-mechanical recyclability potential of biodegradable biopolyesters: Perspectives and limits for food packaging application. Polym. Test..

[363] Lee S.H., Lim S.W., Lee K.H. (1999). Properties of potentially biodegradable copolyesters of (succinic acid-1,4-butanediol)/(dimethyl terephthalate-1,4-butanediol). Polym. Int..

[364] Herrera R., Franco L., Rodríguez-Galán A., Puiggalí J. (2002). Characterization and degradation behavior of poly(butylene adipate-co-terephthalate)s. J. Polym. Sci. Part A Polym. Chem..

[365] Aversa C., Barletta M., Cappiello G., Gisario A. (2022). Compatibilization strategies and analysis of morphological features of poly(butylene adipate-co-terephthalate) (PBAT)/poly(lactic acid) PLA blends: A state-of-art review. Eur. Polym. J..

[366] Song J., Zhou H., Wang X., Zhang Y., Mi J. (2019). Role of chain extension in the rheological properties, crystallization behaviors, and microcellular foaming performances of poly (butylene adipate-co-terephthalate). J. Appl. Polym. Sci..

[367] Song J., Mi J., Zhou H., Wang X., Zhang Y. (2018). Chain extension of poly (butylene adipate-co-terephthalate) and its microcellular foaming behaviors. Polym. Degrad. Stab..

[368] Cui Y., Luo J., Deng Y., Wang X., Zhou H. (2021). Effect of acetylated cellulose nanocrystals on solid-state foaming behaviors of chain-extended poly(butylene adipate-co-terephthalate). J. Vinyl Addit. Technol..

[369] Cui Y., Zhou H., Yin D., Zhou H., Wang X. (2021). An innovative strategy to regulate bimodal cellular structure in chain extended poly(butylene adipate-co-terephthalate) foams. J. Vinyl Addit. Technol..

[370] Malinowski R., Stepczyńska M., Raszkowska-Kaczor A., Żuk T. (2018). Some effects of foaming of the poly(butylene adipate-co-terephthalate) modified by electron radiation. Polym. Adv. Technol..

[371] Cai W., Liu P., Bai S., Li S. (2021). A one-step method to manufacture biodegradable poly (butylene adipate-co-terephthalate) bead foam parts. Polym. Adv. Technol..

[372] Wei X., Cui W., Zheng K., Wang J., Hu J., Zhou H. (2022). Bimodal Cellular Structure Evolution in PBAT Foams Incorporated by Carbon Nanotubes and Graphene Nanosheets. J. Polym. Environ..

[373] Pereira da Silva J.S., Farias da Silva J.M., Soares B.G., Livi S. (2017). Fully biodegradable composites based on poly(butylene adipate-co-terephthalate)/peach palm trees fiber. Compos. Part B Eng..

[374] Hong S.H., Hwang S.H. (2022). Construction and Characterization of Biodegradable Foam from High-Content Lignin-Reinforced Poly(Butylene Adipate-co-Terephthalate) Biocomposites. ACS Appl. Polym. Mater..

[375] Tian H.L., Yu J.S., Zhao Y., Pan H.W., Li Y., Xiao Y., Han L.J., Bian J.J., Hao Y.P., Zhang H.L. (2023). Environmentally friendly poly(butylene adipate-co-terephthalate) and CO_2_-based poly(propylene carbonate) biodegradable foams modified with short basalt fiber. J. Therm. Anal. Calorim..

[376] Javadi A., Srithep Y., Clemons C.C., Turng L.S., Gong S. (2012). Processing of poly(hydroxybutyrate-co-hydroxyvalerate)-based bionanocomposite foams using supercritical fluids. J. Mater. Res..

[377] Fernandes E.M., Pires R.A., Mano J.F., Reis R.L. (2013). Bionanocomposites from lignocellulosic resources: Properties, applications and future trends for their use in the biomedical field. Prog. Polym. Sci..

[378] Lobo F.C.M., Franco A.R., Fernandes E.M., Reis R.L. (2021). An Overview of the Antimicrobial Properties of Lignocellulosic Materials. Molecules.

[379] Le Corre D., Bras J., Dufresne A. (2010). Starch nanoparticles: A review. Biomacromolecules.

[380] Figueiró C.d.S., Calcagno C.I.W., Santana R.M.C. (2023). Starch Foams and Their Additives: A Brief Review. Starch-Stärke.

[381] Cruz-Tirado J.P., Vejarano R., Tapia-Blácido D.R., Barraza-Jáuregui G., Siche R. (2019). Biodegradable foam tray based on starches isolated from different Peruvian species. Int. J. Biol. Macromol..

[382] Han J.H., Lee J., Kim S.K., Kang D.H., Park H.B., Shim J.K. (2023). Impact of the Amylose/Amylopectin Ratio of Starch-Based Foams on Foaming Behavior, Mechanical Properties, and Thermal Insulation Performance. ACS Sustain. Chem. Eng..

[383] Cazón P., Velazquez G., Ramírez J.A., Vázquez M. (2017). Polysaccharide-based films and coatings for food packaging: A review. Food Hydrocoll..

[384] Meng L., Liu H., Yu L., Duan Q., Chen L., Liu F., Shao Z., Shi K., Lin X. (2019). How water acting as both blowing agent and plasticizer affect on starch-based foam. Ind. Crops Prod..

[385] Vieira M.G.A., Da Silva M.A., Dos Santos L.O., Beppu M.M. (2011). Natural-based plasticizers and biopolymer films: A review. Eur. Polym. J..

[386] Sun G., Zeng G., Hu C., Wang M. (2022). Research progress on the application of tristate water in preparation of starch-based foaming materials. Polym. Eng. Sci..

[387] Tabasum S., Younas M., Zaeem M.A., Majeed I., Majeed M., Noreen A., Iqbal M.N., Zia K.M. (2019). A review on blending of corn starch with natural and synthetic polymers, and inorganic nanoparticles with mathematical modeling. Int. J. Biol. Macromol..

[388] Wiercigroch E., Szafraniec E., Czamara K., Pacia M.Z., Majzner K., Kochan K., Kaczor A., Baranska M., Malek K. (2017). Raman and infrared spectroscopy of carbohydrates: A review. Spectrochim. Acta-Part A Mol. Biomol. Spectrosc..

[389] Dircio-Morales M.A., Fonseca-Florido H.A., Velazquez G., Ávila-Orta C.A., Ramos-De Valle L.F., Hernández-Gámez F., Rivera-Salinas J.E., Soriano-Corral F. (2023). Relationship among Extrusion Conditions, Cell Morphology, and Properties of Starch-Based Foams—A Review. Starch-Stärke.

[390] Aguilar G.J., Tapia-Blácido D.R. (2023). Evaluating how avocado residue addition affects the properties of cassava starch-based foam trays. Int. J. Biol. Macromol..

[391] Barbosa J.V., Martins J., Carvalho L., Bastos M.M.S.M., Magalhães F.D. (2019). Effect of peroxide oxidation on the expansion of potato starch foam. Ind. Crops Prod..

[392] Bergel B.F., Araujo L.L., Santana R.M.C. (2022). Evaluation of toxicity and biodegradation of thermoplastic starch foams with modified starch. Food Packag. Shelf Life.

[393] Pornsuksomboon K., Holló B.B., Szécsényi K.M., Kaewtatip K. (2016). Properties of baked foams from citric acid modified cassava starch and native cassava starch blends. Carbohydr. Polym..

[394] Hassan M.M., Tucker N., Le Guen M.J. (2020). Thermal, mechanical and viscoelastic properties of citric acid-crosslinked starch/cellulose composite foams. Carbohydr. Polym..

[395] Iriani E.S., Wahyuningsih K., Oktavia E. (2020). The Effect of Surface Modification by Sizing Agent on the Water Absorption Capacity of Cassava Starch-based Biofoam Packaging. Macromol. Symp..

[396] Rostamabadi H., Bajer D., Demirkesen I., Kumar Y., Su C., Wang Y., Nowacka M., Singha P., Falsafi S.R. (2023). Starch modification through its combination with other molecules: Gums, mucilages, polyphenols and salts. Carbohydr. Polym..

[397] Bergel B.F., da Luz L.M., Santana R.M.C. (2018). Effect of poly(lactic acid) coating on mechanical and physical properties of thermoplastic starch foams from potato starch. Prog. Org. Coat..

[398] Reis M.O., Olivato J.B., Bilck A.P., Zanela J., Grossmann M.V.E., Yamashita F. (2018). Biodegradable trays of thermoplastic starch/poly (lactic acid) coated with beeswax. Ind. Crops Prod..

[399] Chiarathanakrit C., Riyajan S.A., Kaewtatip K. (2018). Transforming fish scale waste into an efficient filler for starch foam. Carbohydr. Polym..

[400] Nansu W., Ross S., Ross G., Mahasaranon S. (2021). Coconut residue fiber and modified coconut residue fiber on biodegradable composite foam properties. Mater. Today Proc..

[401] Ghanbari A., Tabarsa T., Ashori A., Shakeri A., Mashkour M. (2018). Thermoplastic starch foamed composites reinforced with cellulose nanofibers: Thermal and mechanical properties. Carbohydr. Polym..

[402] Moo-Tun N.M., Iñiguez-Covarrubias G., Valadez-Gonzalez A. (2020). Assessing the effect of PLA, cellulose microfibers and CaCO_3_ on the properties of starch-based foams using a factorial design. Polym. Test..

[403] Machado C.M., Benelli P., Tessaro I.C. (2017). Sesame cake incorporation on cassava starch foams for packaging use. Ind. Crops Prod..

[404] Ketkaew S., Kasemsiri P., Hiziroglu S., Mongkolthanaruk W., Wannasutta R., Pongsa U., Chindaprasirt P. (2018). Effect of Oregano Essential Oil Content on Properties of Green Biocomposites Based on Cassava Starch and Sugarcane Bagasse for Bioactive Packaging. J. Polym. Environ..

[405] Mahmud M.A., Belal S.A., Gafur M.A. (2023). Development of a biocomposite material using sugarcane bagasse and modified starch for packaging purposes. J. Mater. Res. Technol..

[406] Engel J.B., Ambrosi A., Tessaro I.C. (2019). Development of biodegradable starch-based foams incorporated with grape stalks for food packaging. Carbohydr. Polym..

[407] Engel J.B., Ambrosi A., Tessaro I.C. (2019). Development of a Cassava Starch-Based Foam Incorporated with Grape Stalks Using an Experimental Design. J. Polym. Environ..

[408] Machado C.M., Benelli P., Tessaro I.C. (2020). Study of interactions between cassava starch and peanut skin on biodegradable foams. Int. J. Biol. Macromol..

[409] Zhang C., Zhang P., Li Y., Sandeep S.N., Li J., Ji M., Peng S., Yan N., Li F. (2023). Fully biodegradable, hydrophobic, enhanced barrier starch bio-composites with sandwich structure by simulating wood. Ind. Crops Prod..

[410] Cheng X.-H., Wang K., Cheng N.-Q., Mi S.-Y., Sun L.-S., Yeh J.-T. (2021). The control of expansion ratios and cellular structure of supercritical CO_2_-aid thermoplastic starch foams using crosslinking agents and nano-silica particles. J. Polym. Res..

[411] Figueiró C.D., Trojaner M.R., Calcagno C.I.W., Santana R.M.C. (2023). Effect of Silica Content on Cellular Structural and Hygroscopicity of Modified Cassava Starch Foam. Starch-Starke.

[412] Guo A., Tao X., Kong H., Zhou X., Wang H., Li J., Li F., Hu Y. (2023). Effects of aluminum hydroxide on mechanical, water resistance, and thermal properties of starch-based fiber-reinforced composites with foam structures. J. Mater. Res. Technol..

[413] Chiarathanakrit C., Mayakun J., Prathep A., Kaewtatip K. (2019). Comparison of the effects of calcified green macroalga (Halimeda macroloba Decaisne) and commercial CaCO3 on the properties of composite starch foam trays. Int. J. Biol. Macromol..

[414] Kaewtatip K., Chiarathanakrit C., Riyajan S.A. (2018). The effects of egg shell and shrimp shell on the properties of baked starch foam. Powder Technol..

[415] Rodrigues N.H.P., de Souza J.T., Rodrigues R.L., Canteri M.H.G., Tramontin S.M.K., de Francisco A.C. (2020). Starch-Based Foam Packaging Developed from a By-Product of Potato Industrialization (*Solanum tuberosum* L.). Appl. Sci..

[416] Versino F., López O.V., García M.A. (2021). Sunflower Oil Industry By-product as Natural Filler of Biocomposite Foams for Packaging Applications. J. Polym. Environ..

[417] Nugroho A., Maharani D.M., Legowo A.C., Hadi S., Purba F. (2022). Enhanced mechanical and physical properties of starch foam from the combination of water hyacinth fiber (*Eichhornia crassipes*) and polyvinyl alcohol. Ind. Crops Prod..

[418] Kaewtatip K., Saepoo T., Sarak S., Mayakun J., Chaibundit C. (2023). Preparation and characterization of biodegradable starch foam composite with treated Khlum fiber for food packaging. J. Appl. Polym. Sci..

[419] Sanhawong W., Banhalee P., Boonsang S., Kaewpirom S. (2017). Effect of concentrated natural rubber latex on the properties and degradation behavior of cotton-fiber-reinforced cassava starch biofoam. Ind. Crops Prod..

[420] Joshi P., Gupta K., Uniyal P., Jana A., Banerjee A., Kumar N., Ghosh D., Srivastava M., Ray A., Khatri O.P. (2023). Cassava starch-derived aerogels as biodegradable packaging materials. Mater. Chem. Phys..

[421] Kahvand F., Fasihi M. (2020). Microstructure and physical properties of thermoplastic corn starch foams as influenced by polyvinyl alcohol and plasticizer contents. Int. J. Biol. Macromol..

[422] Cruz-Tirado J.P., Barros Ferreira R.S., Lizárraga E., Tapia-Blácido D.R., Silva N.C.C., Angelats-Silva L., Siche R. (2020). Bioactive Andean sweet potato starch-based foam incorporated with oregano or thyme essential oil. Food Packag. Shelf Life.

[423] Velasco V., Sepúlveda E., Williams P., Rodríguez-Llamazares S., Gutiérrez C., Valderrama N. (2022). Starch-based composite foam for chicken meat packaging. J. Food Sci. Technol..

[424] Bahramian B., Fathi A., Dehghani F. (2016). A renewable and compostable polymer for reducing consumption of non-degradable plastics. Polym. Degrad. Stab..

[425] Cvek M., Paul U.C., Zia J., Mancini G., Sedlarik V., Athanassiou A. (2022). Biodegradable Films of PLA/PPC and Curcumin as Packaging Materials and Smart Indicators of Food Spoilage. ACS Appl. Mater. Interfaces.

[426] Ling Y., Li X., Gao P., Wu M., Wang L., Zheng W. (2023). Lightweight biodegradable porous poly(propylene carbonate)/carbon nanostructures nano/microcellular structures with enhanced foamability, good electromagnetic interference shielding, and low permanent strain. Compos. Commun..

[427] Muthuraj R., Mekonnen T. (2018). Carbon Dioxide–Derived Poly(propylene carbonate) as a Matrix for Composites and Nanocomposites: Performances and Applications. Macromol. Mater. Eng..

[428] Kuang T., Li K., Chen B., Peng X. (2017). Poly (propylene carbonate)-based in situ nanofibrillar biocomposites with enhanced miscibility, dynamic mechanical properties, rheological behavior and extrusion foaming ability. Compos. Part B Eng..

[429] Yang G., Su J., Gao J., Hu X., Geng C., Fu Q. (2013). Fabrication of well-controlled porous foams of graphene oxide modified poly(propylene-carbonate) using supercritical carbon dioxide and its potential tissue engineering applications. J. Supercrit. Fluids.

[430] Cui X., Chen J., Zhu Y., Jiang W. (2018). Lightweight and conductive carbon black/chlorinated poly(propylene carbonate) foams with a remarkable negative temperature coefficient effect of resistance for temperature sensor applications. J. Mater. Chem. C.

[431] Jiao J., Xiao M., Shu D., Li L., Meng Y.Z. (2006). Preparation and characterization of biodegradable foams from calcium carbonate reinforced poly(propylene carbonate) composites. J. Appl. Polym. Sci..

[432] Yu P., Mi H.-Y., Huang A., Liu X., Chen B.-Y., Zhang S.-D., Peng X.-F. (2015). Preparation of poly(propylene carbonate)/nano calcium carbonate composites and their supercritical carbon dioxide foaming behavior. J. Appl. Polym. Sci..

[433] Sartore L., Inverardi N., Pandini S., Bignotti F., Chiellini F. (2019). PLA/PCL-based foams as scaffolds for tissue engineering applications. Mater. Today Proc..

[434] Sun S., Li Q., Zhao N., Jiang J., Zhang K., Hou J., Wang X., Liu G. (2018). Preparation of highly interconnected porous poly(ε-caprolactone)/poly(lactic acid) scaffolds via supercritical foaming. Polym. Adv. Technol..

[435] Zhao N., Lv Z., Ma J., Zhu C., Li Q. (2019). Fabrication of hydrophilic small diameter vascular foam scaffolds of poly(ε-caprolactone)/polylactic blend by sodium hydroxide solution. Eur. Polym. J..

[436] Wang L., Wang D., Zhou Y., Zhang Y., Li Q., Shen C. (2019). Fabrication of open-porous PCL/PLA tissue engineering scaffolds and the relationship of foaming process, morphology, and mechanical behavior. Polym. Adv. Technol..

[437] Lv Z., Zhao N., Wu Z., Zhu C., Li Q. (2018). Fabrication of Novel Open-Cell Foams of Poly(ε-caprolactone)/Poly(lactic acid) Blends for Tissue-Engineering Scaffolds. Ind. Eng. Chem. Res..

[438] Qiao Y., Li Q., Jalali A., Yang J., Wang X., Zhao N., Jiang Y., Wang S., Hou J., Jiang J. (2021). In-situ microfibrillated Poly(ε-caprolactone)/Poly(lactic acid) composites with enhanced rheological properties, crystallization kinetics and foaming ability. Compos. Part B Eng..

[439] Cao Y., Jiang J., Jiang Y., Li Z., Hou J., Li Q. (2022). Biodegradable highly porous interconnected poly(ε-caprolactone)/poly(L-lactide-co-ε-caprolactone) scaffolds by supercritical foaming for small-diameter vascular tissue engineering. Polym. Adv. Technol..

[440] Kong W., Li R., Zhao X., Ye L. (2023). Construction of a Highly Oriented Poly(lactic acid)-Based Block Polymer Foam and Its Self-Reinforcing Mechanism. ACS Sustain. Chem. Eng..

[441] Geissler B., Feuchter M., Laske S., Walluch M., Holzer C., Langecker G.R. (2014). Tailor-Made High Density PLA Foam Sheets—Strategies to Improve the Mechanical Properties. Cell. Polym..

[442] Li B., Zhao G., Wang G., Zhang L., Gong J., Shi Z. (2021). Biodegradable PLA/PBS open-cell foam fabricated by supercritical CO_2_ foaming for selective oil-adsorption. Sep. Purif. Technol..

[443] Sun Z., Wang L., Zhou J., Fan X., Xie H., Zhang H., Zhang G., Shi X. (2020). Influence of Polylactide (PLA) Stereocomplexation on the Microstructure of PLA/PBS Blends and the Cell Morphology of Their Microcellular Foams. Polymers.

[444] Yu P., Mi H.Y., Huang A., Geng L.H., Chen B.Y., Kuang T.R., Mou W.J., Peng X.F. (2015). Effect of Poly(butylenes succinate) on Poly(lactic acid) Foaming Behavior: Formation of Open Cell Structure. Ind. Eng. Chem. Res..

[445] Tian G., He H., Xu M., Liu Y., Gao Q., Zhu Z. (2023). Ultralow percolation threshold biodegradable PLA/PBS/MWCNTs with segregated conductive networks for high-performance electromagnetic interference shielding applications. J. Appl. Polym. Sci..

[446] Campuzano J.F., López I.D. (2020). Study of the effect of dicumyl peroxide on morphological and physical properties of foam injection molded poly(Lactic acid)/poly(butylene succinate) blends. Express Polym. Lett..

[447] Chen J., Yang L., Chen D., Wang M., Wu L. (2023). Facile fabrication of highly interconnected poly(lactic acid)-based scaffolds with good hydrophilicity using supercritical carbon dioxide. J. Appl. Polym. Sci..

[448] Chen P., Gao X., Zhao L., Xu Z., Li N., Pan X., Dai J., Hu D. (2022). Preparation of biodegradable PBST/PLA microcellular foams under supercritical CO_2_: Heterogeneous nucleation and anti-shrinkage effect of PLA. Polym. Degrad. Stab..

[449] Long H., Xu H., Shaoyu J., Jiang T., Zhuang W., Li M., Jin J., Ji L., Ying H., Zhu C. (2023). High-Strength Bio-Degradable Polymer Foams with Stable High Volume-Expansion Ratio Using Chain Extension and Green Supercritical Mixed-Gas Foaming. Polymers.

[450] Liu W., He S., Zhou H. (2018). Preparation and properties of flexible poly(lactic acid) blend foams. Cell. Polym..

[451] Liu W., Chen P., Wang X., Wang F., Wu Y. (2017). Effects of poly(butyleneadipate-co-terephthalate) as a macromolecular nucleating agent on the crystallization and foaming behavior of biodegradable poly(lactic acid). Cell. Polym..

[452] Shi X., Qin J., Wang L., Ren L., Rong F., Li D., Wang R., Zhang G. (2018). Introduction of stereocomplex crystallites of PLA for the solid and microcellular poly(lactide)/poly(butylene adipate-*co*-terephthalate) blends. RSC Adv..

[453] Jenkins M.J., Cao Y., Howell L., Leeke G.A. (2007). Miscibility in blends of poly(3-hydroxybutyrate-*co*-3-hydroxyvalerate) and poly(ε-caprolactone) induced by melt blending in the presence of supercritical CO_2_. Polymer.

[454] Brütting C., Dreier J., Bonten C., Altstädt V., Ruckdäschel H. (2023). Sustainable Immiscible Polylactic Acid (PLA) and Poly(3-hydroxybutyrate-*co*-3-hydroxyvalerate) (PHBV) Blends: Crystallization and Foaming Behavior. ACS Sustain. Chem. Eng..

[455] Walallavita A., Verbeek C.J.R., Lay M. (2016). Blending Novatein^®^ thermoplastic protein with PLA for carbon dioxide assisted batch foaming. Proceedings of the 31st International Conference of the Polymer Processing Society—Conference Papers.

[456] Walallavita A.S., Verbeek C.J.R., Lay M.C. (2017). Biopolymer foams from Novatein thermoplastic protein and poly(lactic acid). J. Appl. Polym. Sci..

[457] Hu D., Xue K., Liu Z., Xu Z., Zhao L. (2022). The essential role of PBS on PBAT foaming under supercritical CO_2_ toward green engineering. J. CO_2_ Util..

[458] Li Y., Zhang Z., Wang W., Gong P., Yang Q., Park C.B., Li G. (2022). Ultra-fast degradable PBAT/PBS foams of high performance in compression and thermal insulation made from environment-friendly supercritical foaming. J. Supercrit. Fluids.

[459] Peng J., Zhang C., Mi H., Peng X.F., Turng L.S. (2014). Study of solid and microcellular injection-molded poly(butylenes adipate- co -terephthalate)/poly(vinyl alcohol) biodegradable parts. Ind. Eng. Chem. Res..

[460] Tian H.-l., Wang Z.-p., Jia S.-l., Pan H.-w., Han L.-j., Bian J.-j., Li Y., Yang H.-L., Zhang H.-L. (2022). Biodegradable Foaming Material of Poly(butylene adipate-co-terephthalate) (PBAT)/Poly(propylene carbonate) (PPC). Chin. J. Polym. Sci..

[461] Boonprasertpoh A., Pentrakoon D., Junkasem J. (2020). Effect of PBAT on physical, morphological, and mechanical properties of PBS/PBAT foam. Cell. Polym..

[462] Xu C., Sun C., Wan H., Tan H., Zhao J., Zhang Y. (2022). Microstructure and physical properties of poly(lactic acid)/polycaprolactone/rice straw lightweight bio-composite foams for wall insulation. Constr. Build. Mater..

[463] Guo H., Jiang J., Li Z., Jin Z., Hou J., Wang X., Li Q. (2020). Solid-State Supercritical CO_2_ Foaming of PCL/PLGA Blends: Cell Opening and Compression Behavior. J. Polym. Environ..

[464] Hassan M.M., Le Guen M.J., Tucker N., Parker K. (2019). Thermo-mechanical, morphological and water absorption properties of thermoplastic starch/cellulose composite foams reinforced with PLA. Cellulose.

[465] Chauvet M., Sauceau M., Baillon F., Fages J. (2021). Blending and foaming thermoplastic starch with poly (lactic acid) by CO_2_-aided hot melt extrusion. J. Appl. Polym. Sci..

[466] Trinh B.M., Chang C.C., Mekonnen T.H. (2021). Facile fabrication of thermoplastic starch/poly (lactic acid) multilayer films with superior gas and moisture barrier properties. Polymer.

[467] Chang C.-J., Venkatesan M., Cho C.-J., Chung P.-Y., Chandrasekar J., Lee C.-H., Wang H.-T., Wong C.-M., Kuo C.-C. (2022). Thermoplastic Starch with Poly(butylene adipate-co-terephthalate) Blends Foamed by Supercritical Carbon Dioxide. Polymers.

[468] Ogunsona E., D’Souza N.A. (2015). Characterization and mechanical properties of foamed poly(ε-caprolactone) and Mater-Bi blends using CO_2_ as blowing agent. J. Cell. Plast..

[469] Martin Torrejon V., Song H., Wu B., Luo G., Song J. (2023). Effect of Starch Type and Pre-Treatment on the Properties of Gelatin–Starch Foams Produced by Mechanical Foaming. Polymers.

[470] Vidal F., van der Marel E.R., Kerr R.W.F., McElroy C., Schroeder N., Mitchell C., Rosetto G., Chen T.T.D., Bailey R.M., Hepburn C. (2024). Designing a circular carbon and plastics economy for a sustainable future. Nature.

[471] Rahman M.H., Bhoi P.R. (2021). An overview of non-biodegradable bioplastics. J. Clean. Prod..

[472] Transforming our World: The 2030 Agenda for Sustainable Development. https://sdgs.un.org/publications/transforming-our-world-2030-agenda-sustainable-development-17981.

[473] Yang M., Chen L., Wang J., Msigwa G., Osman A.I., Fawzy S., Rooney D.W., Yap P.-S. (2023). Circular economy strategies for combating climate change and other environmental issues. Environ. Chem. Lett..

[474] The European Green Deal. https://commission.europa.eu/strategy-and-policy/priorities-2019-2024/story-von-der-leyen-commission/european-green-deal_en.

[475] U.S. Plastics Pact Roadmap to 2025. https://usplasticspact.org/roadmap/.

[476] European Commission A New Circular Economy Action Plan for a Cleaner and More Competitive Europe. https://eur-lex.europa.eu/legal-content/EN/TXT/?qid=1583933814386&uri=COM:2020:98:FIN.

[477] Terzopoulou Z., Bikiaris D.N. (2024). Biobased plastics for the transition to a circular economy. Mater. Lett..

[478] Biodegradable Foam Market Analysis. https://www.coherentmarketinsights.com/market-insight/biodegradable-foam-market-5970.

[479] European Commission: Biobased, Biodegradable and Compostable Plastics. https://environment.ec.europa.eu/topics/plastics/biobased-biodegradable-and-compostable-plastics_en.

[480] Wang X., Jang J., Su Y., Liu J., Zhang H., He Z., Ni Y. (2024). Starting materials, processes and characteristics of bio-based foams: A review. J. Bioresour. Bioprod..

